# Systematic review of observational coding systems to assess patient-healthcare professional interactions

**DOI:** 10.1016/j.pec.2025.108718

**Published:** 2025-02-27

**Authors:** Marij A. Hillen, Kerri-Anne R. Mitchell, Barbara C. Schouten, John W. Cyrus, Richard F. Brown, Arwen H. Pieterse

**Affiliations:** a Department of Medical Psychology, Amsterdam Public Health, Amsterdam University Medical Centers, the Netherlands; b Public Health Program, Austin College, Sherman, TX, USA; c University of Amsterdam, Department of Communication, Amsterdam School of Communication Research/ASCoR, Center for Urban Mental Health, the Netherlands; d Research and Education Librarian, Health Sciences Library, Virginia Commonwealth University, 509 N. 12th Street, Richmond, VA 23298, USA; e Department of Health Behavior and Policy, Virginia Commonwealth University School of Medicine, Richmond, VA, USA; f Biomedical Data Sciences, Leiden University Medical Center, the Netherlands; g Institute of Clinical Medicine, University of Oslo, Norway

**Keywords:** Patient-provider interaction, Clinical consultation, Interaction analysis, Observational coding system, Systematic review, Psychometric evaluation

## Abstract

**Objectives:**

Systematic coding is used to study interactions between patients and healthcare professionals from an independent observer perspective. Many coding systems are available, but an up-to-date overview is lacking. We aimed to: (1) provide a comprehensive overview of systems for systematic coding of patient-healthcare professional interactions; and describe their 2) general characteristics and development, and 3) validation and adaptation.

**Methods:**

We systematically searched peer-reviewed empirical articles in five databases (Cochrane, Embase, PubMed/Medline, CINAHL, PsychINFO) using variations of the following keywords: (i) patient and/or other stakeholder, (ii) healthcare professional (iii), interactions in healthcare, (iv) coding tool, and (v) development and/or validation. All titles/abstracts and full-texts were screened independently and in duplicate. Additionally, coding systems were identified through an earlier review, an open-access research database, and a forward-reference search of all included coding systems up to that point. For all eligible systems, we extracted characteristics and psychometric properties.

**Results:**

From a total of 6950 identified articles from literature databases, 188 full-text articles were screened. Thirty-five articles were included from additional sources. In total, we included 124 articles describing 98 coding systems. Systems were highly variable in terms of topic (e.g., patient-centered communication, shared decision making, behavior change counseling), clinical context (e.g., oncology, pediatrics, generic), rigor of development and reporting, coding complexity, and extent of psychometric testing. Inter-rater reliability was reported for most coding systems; only few were tested for other types of reliability or for validity.

**Conclusions:**

A plethora of coding systems are available, but more systematic reporting and psychometric testing are urgently needed to enhance evidence of validity. Testing may initially focus on the most relevant and broadly applicable coding systems.

**Practice implications:**

These results can aid researchers in selecting the most suitable coding system for their purposes. Researchers may consider using or adapting existing systems, before developing new coding systems.

## 1. Introduction

Effective communication between patients and healthcare professionals is universally considered an essential component of patient centered health care [1]. Research efforts to elucidate how communication occurs during healthcare interactions have proliferated over the past decades [2]. The increasing interest in communication in healthcare was initially ideologically driven by the shift away from paternalistic patient care –characterized by physician-directed communication– towards patient-centered care –characterized by patient empowerment in communication and decision making [3,4]. Patient-centered care is now broadly considered the gold standard of care in many countries. Over time, the call to empirically substantiate the benefits of patient-centered communication practices has grown [3–5]. This has led to a proliferation of research to further disentangle the nature and impact of patient-healthcare professional communication.

A variety of conceptual models have been developed that clarify the various functions of effective patient-healthcare professional communication. While there are slight variations between these models, six analogous functions can be identified, i.e., fostering the patient-health professional relationship, gathering and providing information, (sharing) decision making, enabling disease- and treatment-related patient behavior, and responding to patient emotions [6,7]. Healthcare professionals’ communicative actions in interactions with patients will invariably contribute to the success or failure in serving one or several of these functions, ultimately affecting the outcomes of care. In parallel, consensus statements and guidelines have been developed to inform clinical communication skills training and assessment that guide trainers in how to foster effective clinical communication [8,9]. The emergence of conceptual models and clinical communication skills training have helped scaffold the assessment of patient-professional communication and its association with other important aspects of high-quality healthcare.

A mounting body of research evidence suggests that effective patient-healthcare professional interactions are associated with patients’ improved comprehension of information, sense of well-being, health-related behavior (e.g., following treatment advice), and quality of life [10–13]. Indeed, patient-professional interaction is thought to affect patient long-term outcomes through indirect pathways. Healthcare professionals’ specific interaction behaviors (immediate outcomes; e.g., structuring information, checking patients’ understanding) are expected to affect intermediate outcomes (e.g., patient understanding, recall, and following treatment advice), which in turn exert an influence on long-term outcomes (e.g., patient/health professional psychological well-being, or patient physical health) [13]. Understanding the pathways between aspects of healthcare interaction and immediate, intermediate and long-term outcomes is critical to improving healthcare delivery, patients’ experience of healthcare, and patient outcomes. Elucidating such pathways demands that researchers specify and reliably assess actual interaction behaviors. Both qualitative and quantitative approaches may be used to yield systematic insights into the processes and quality of patient-healthcare professional interactions, and how these may be supported and improved through interventions.

Research on patient-health professional interactions has often relied on self-report evaluations of the interaction, be it qualitatively through interviews or quantitatively using surveys [2]. Such evaluations provide valuable insights into patient care experiences. However, self-report evaluations are limited in their capacity to capture the specifics of interactions and are susceptible to memory, recall, and social desirability biases, which limit their validity. Systematic coding of observed human behavior (SCOBe), using direct researcher observation or (audio or video) recording, allows for a more accurate (or less biased) assessment of what happens in patient-health professional interactions than self-report evaluations [14]. Furthermore, SCOBe enables measuring the efficacy of communication skills training programs to enhance the quality of healthcare professionals’ interactions, through the assessment of actual versus reported interaction behavior [15–17].

Over time, a plethora of observational coding systems have been developed and used in research to assess various interactional aspects, and reflecting the multi-faceted and complex nature of patient-professional interactions [18]. In spite of the proliferation of observer-based behavioral coding, no recent comprehensive overviews of available coding systems exist in the literature. In 2014, Zill and Scholl [19] created an overview of systems to assess patient-physician interaction. This review was limited in its focus on generic physician-patient interaction systems intended for use across medical contexts. We sought to conduct an updated review that additionally considers coding systems developed for use in specific clinical contexts, to assess specific aspects of interactions, and/or focus on healthcare professionals who are not physicians. This review has several purposes. First, to provide guidance to researchers intending to systematically assess healthcare interactions by identifying available systems, describing their development and contents, and the extent to which their psychometric properties have been assessed. Second, to support the expansion of the body of existing research and systems, thereby fostering efficient use of research resources. Third, to stimulate the improvement and cross-validation of existing systems, resulting in more rigorous research of healthcare interaction.

In sum, the aim of this systematic review was to provide an overview of observer-based coding systems of patient-healthcare professional interaction to meet the three objectives outlined above. Specifically, we inventoried the systems’ focus, development process, adaptation, and evaluation of their psychometric properties.

## 2. Methods

### 2.1. Study design

We conducted a systematic review in accordance with the PRISMA statement [23] to update and expand on a previous systematic review of coding systems for patient-professional interaction [19]. The current review was registered with PROSPERO (Registration no. CRD42021253544).

### 2.2. Search strategy

Five electronic databases (Cochrane (Wiley), Embase (Ovid), Medline (Ovid), CINAHL (EBSCO), and PsycINFO (EBSCO)) were systematically searched for peer-reviewed articles from their inception up to April 30, 2021. An update search was run on December 12, 2023. A health sciences librarian experienced in systematic reviews (JC) assisted with the search strategy development, search process, and documentation. The final search strategy consisted of controlled vocabulary and free-text terms for the following five domains: (i) patient and/or other stakeholder (ii) - provider (iii) interactions in healthcare, (iv) coding tool, and (v) development and/or evaluation. Each of the search domains was combined by the Boolean operator AND. Additionally, a sixth search group with specific terms for publication types was added to the search strategy with the Boolean operator NOT, to exclude non-English publications, non-peer-reviewed publications such as editorials and comments, and papers about animals. The terms observation, observational, observer were considered for inclusion in the initial coding system concept of the search, but were found to introduce considerable noise to the search results. The full search strategy for each database can be found in Appendix A. Note that controlled vocabulary terms, such as MeSH in PubMed/Medline, were tested across databases. Lack of consistency in assignment of controlled vocabulary to known relevant articles limited their usefulness. Comprehensive keyword searches were implemented instead with the exception of the validation search strategy.

EndNote 8 (Clarivate Analytics, 2017) was used to manage citations and identify duplicates. Citations were then uploaded to Covidence systematic review software for screening [24]. Additionally, articles were searched through: (1) a previously published systematic review of measures to assess shared decision making [25]; and (2) an existing online database of systems for systematic coding of healthcare interaction (https://each.international/resources/reach/). Once all eligible articles had been included based on the search up to this point, we performed (3) a forward-reference search of these articles to identify studies performing further validation of the identified systems. If newly identified coding systems fit the eligibility criteria they were included.

### 2.3. Eligibility criteria

We included peer-reviewed and published qualitative and quantitative empirical articles that described the development, validation, and/or adaption of systems to systematically code observed behavior in healthcare interaction. Included coding systems focused on interactions between patient and/or patients’ informal caregivers, and healthcare professional. Papers were excluded if they: 1) were not published in English; 2) were non-empirical, such as editorials and comments; 3) focused on the use of an existing coding system only; 4) described a system developed for use in a specific study without reporting details about its development; 5) involved the use of self-report measures; 6) focused on inter-professional interaction; or 7) focused on therapeutic communication (e.g., psychotherapy), with the exception of behavior change counseling/motivational interviewing within the medical setting.

### 2.4. Study review and selection

All retrieved articles were evaluated for eligibility in two steps; first by assessing title and abstract, and second based on the full-text. Both rounds of eligibility assessment were performed by a team of five reviewers (BS, RB, KM, AP, MH). All articles were independently assessed by two reviewers in Covidence. Discrepancies were resolved among reviewer duos via discussion and, if necessary, during full team meetings.

### 2.5. Data extraction

All team members extracted the following data from each article using an abstraction form designed collaboratively: coding system name and acronym, year of publication, first author, country, construct to be assessed and its definition, target population, development/validation sample (N, age, gender, medical context), target actor (whose behavior was assessed), development process, behaviors assessed, response type, coders, and coding process. Additionally, we extracted information about reliability and validity using a relevant selection from the measurement properties as proposed by the Consensus-based Standards for the selection of health Measurement Instruments (COSMIN) [26]. This included content validity, structural validity, internal consistency, intra-rater reliability, inter-rater reliability, criterion validity, convergent validity, discriminative validity, known-groups validity, cross-cultural adaptation, and cross-cultural validity. Alignment between all team members was enhanced through independently extracting and subsequently discussing the first two articles within the team, holding frequent team meetings to discuss any discrepancies, and randomly dividing articles over varying pairs of researchers to avoid evaluator drift. Throughout extraction, discrepancies were resolved among the pairs and discussed within the research team if needed, until consensus was reached.

### 2.6. Data synthesis

Spreadsheet tables were used to aggregate study data and measurement properties from multiple papers addressing the same system, and to summarize the available evidence for each coding system using frequencies and percentages. Information was analyzed according to coding system characteristics (i.e., general characteristics, development process, choice and definition of construct, target population and context, and number of items & response type), and psychometric characteristics (i.e., sample match and coding procedures, validity testing, and reliability testing).

## 3. Results

### 3.1. Search and selection results

**Fig. 1.**
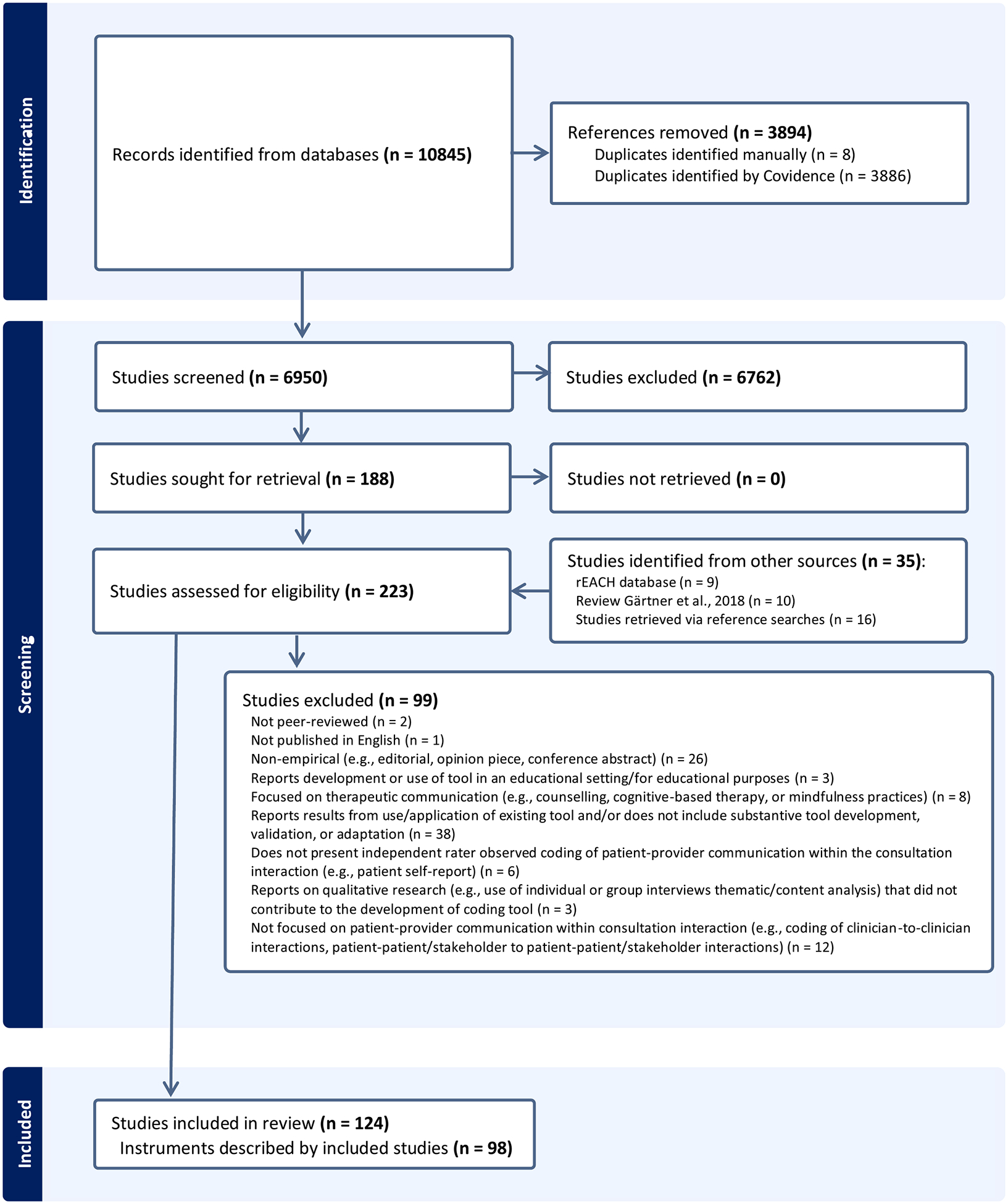
PRISMA-diagram of article search and selection.

Fig. 1 shows the flow chart of the search and selection results. Our database search yielded 6950 unique articles, of which 188 were screened in full-text. An additional 10 articles were included from a previous review [25], and nine from the rEACH online database of coding systems (see Methods, Section 2.2). The forward reference search of these 108 systems yielded 16 additional relevant articles. Eventually, we included 124 articles describing 98 unique coding systems (Table 1). For 84 systems, only one paper describing development, validation, translation and/or adaptation was identified. For the other 14 systems, between two and nine papers were identified. For example, five papers describing additional validity and reliability testing of the OPTION^12item^ [27] have been published.

### 3.2. Coding system characteristics

Characteristics of the coding systems (N = 98) are displayed in Table 1. Supplementary Table 1b (Appendix B) includes further details for all 124 included articles, and Supplementary Table 1c (Appendix C) displays characteristics of the 98 coding systems ordered by interactional focus.

#### 3.2.1. General characteristics

**Fig. 2.**
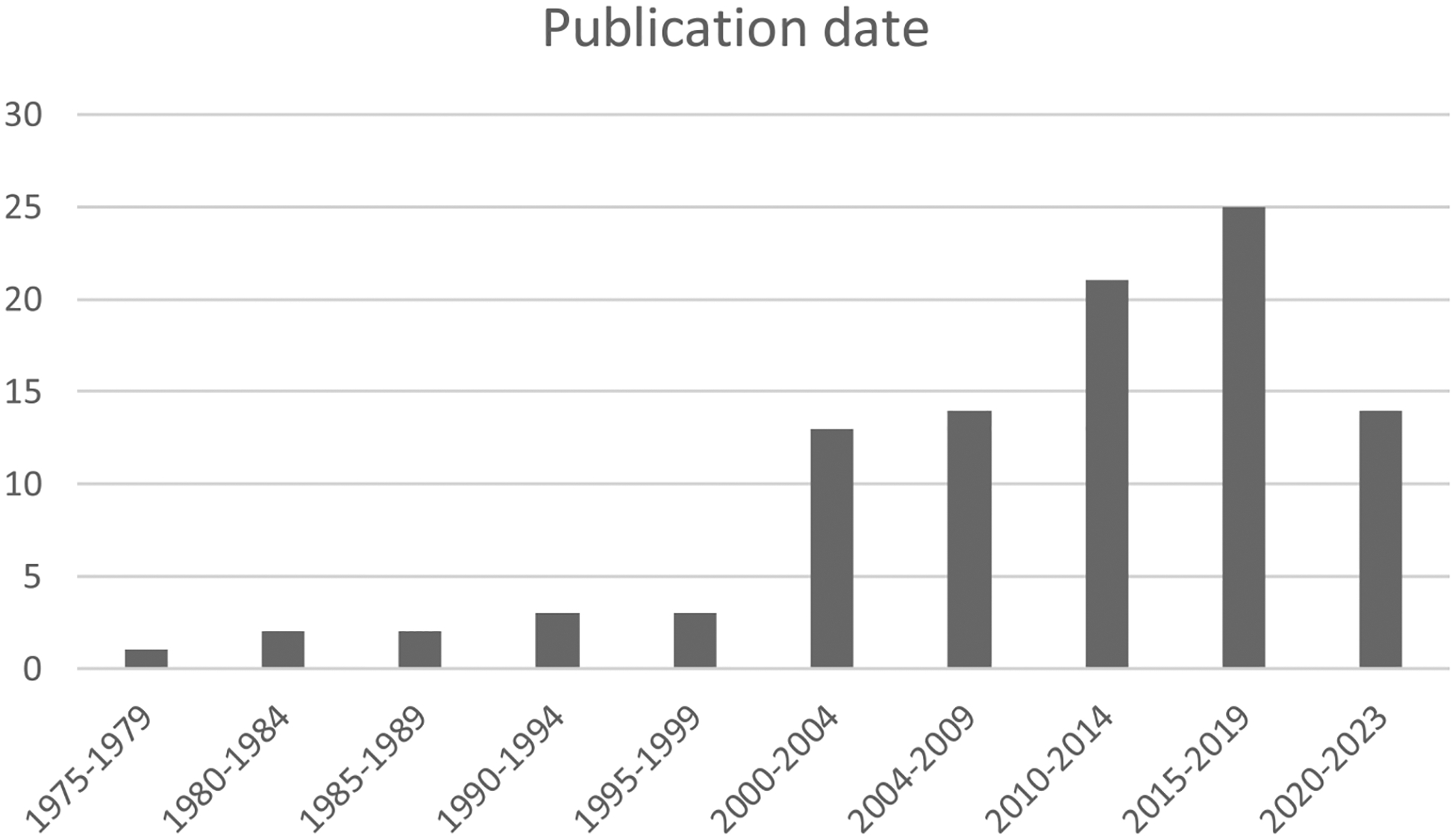
Publication date for the N = 98 included coding systems.

The oldest system identified was published in 1979. The number of available systems has sharply increased, particularly over the last two decades. Almost half (n = 44) were published in the last 10 years (2013–2023; see Fig. 2).

Nearly all systems have been developed in North America (n = 58), Europe or the UK (n = 28), and Australia (n = 4), or a combination of these (n = 3). Two systems were developed, respectively, in Israel and South Africa. For three systems, country of development was not reported or unclear. Articles that described validation or cultural/language adaptation of existing systems reported on research conducted in North America (n = 5), Europe and the UK (n = 17), Australia (n = 2), or a combination of these (n = 3), and five in other countries (n = 4 from China, n = 1 from Brazil).

#### 3.2.2. Development process

Extensiveness of reporting about the development process varied widely between coding systems. For some, the development was based on the authors’ knowledge and experience and/or the literature. Other systems were the subject of an extensive development process that sometimes involved multiple rounds of refinement, and included the retrieval of information from existing frameworks, expert panels, clinical guidelines, existing systems, and/or content coding of recorded interactions (Table 1, e.g., VR-CoDES [28]). No information about development or pilot testing was provided for nine coding systems. Of the 89 systems that included reporting about development, 45 did not report pilot testing prior to finalizing the system. The majority of articles (n = 86) reported the development and/or validation of the coding system as the main objective of the study. In others (n = 12), the development was conducted in order to directly apply it for specific study purposes, e.g., testing hypotheses.

#### 3.2.3. Choice of construct and construct definition

**Fig. 3.**
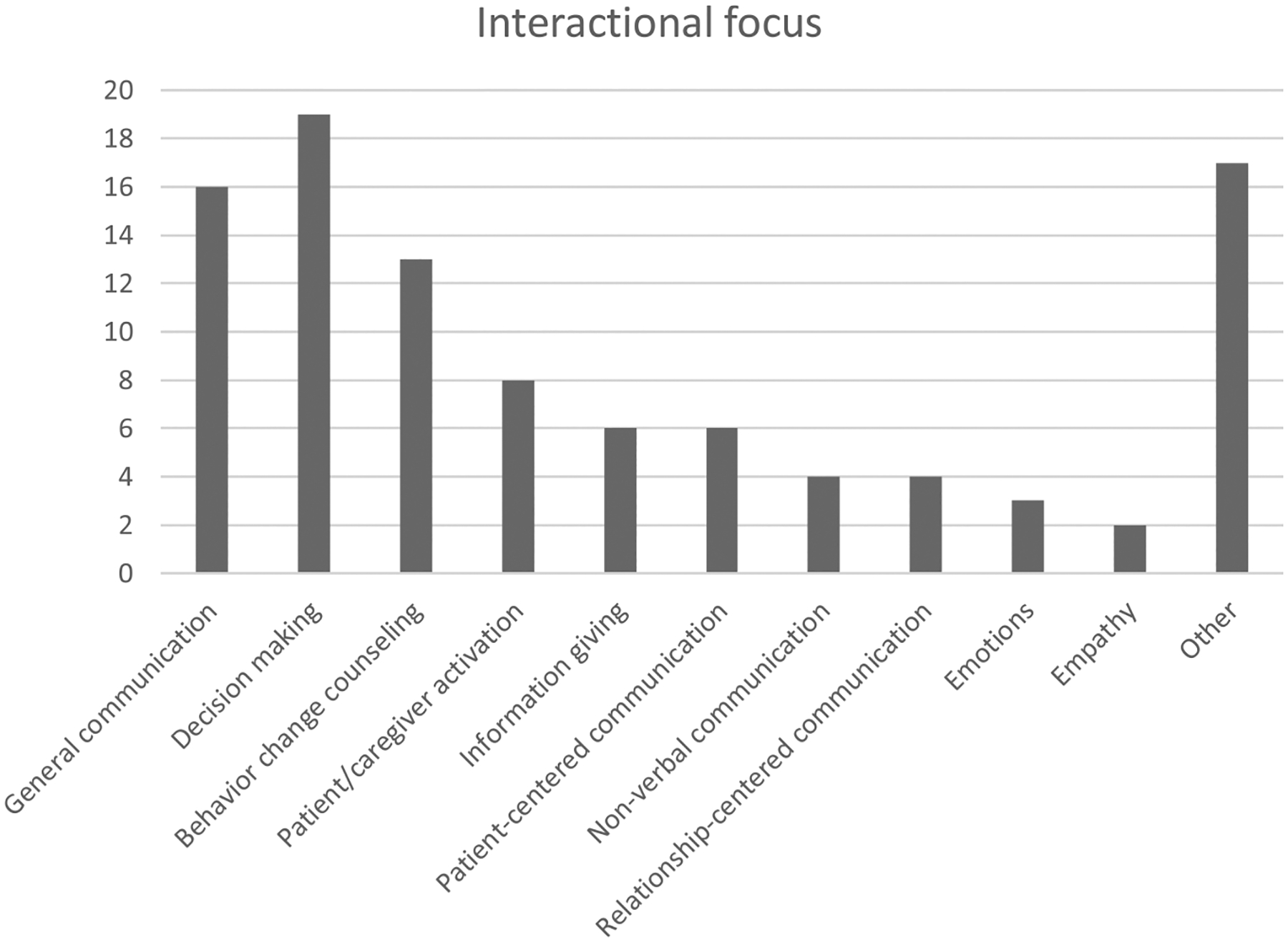
Interactional focus of the included coding systems.

Fig. 3 displays frequencies of coding system foci, and supplementary Table 1c (Appendix C) displays coding systems ordered by interactional focus. Sixteen systems assess interaction behaviors broadly. Overall, the most prevalent specific constructs that other systems assess are decision making (n = 19), behavior change counseling (n = 13), and patient-centered communication (n = 8). The 24 remaining systems focus on specific interaction elements, for example metaphor use [29], advance care planning [30], relational style [31], triadic interactions [32], and interaction style with electronic health records during consultations [33]. Various systems included some non-verbal aspects as part of a broader range of behaviors, and four focused exclusively on non-verbal communication, such as touch or eye contact [34–37]. Sixty-four articles provided information about the construct definition –with varying degrees of specificity– while 24 articles did not.

#### 3.2.4. Target population and context

**Fig. 4.**
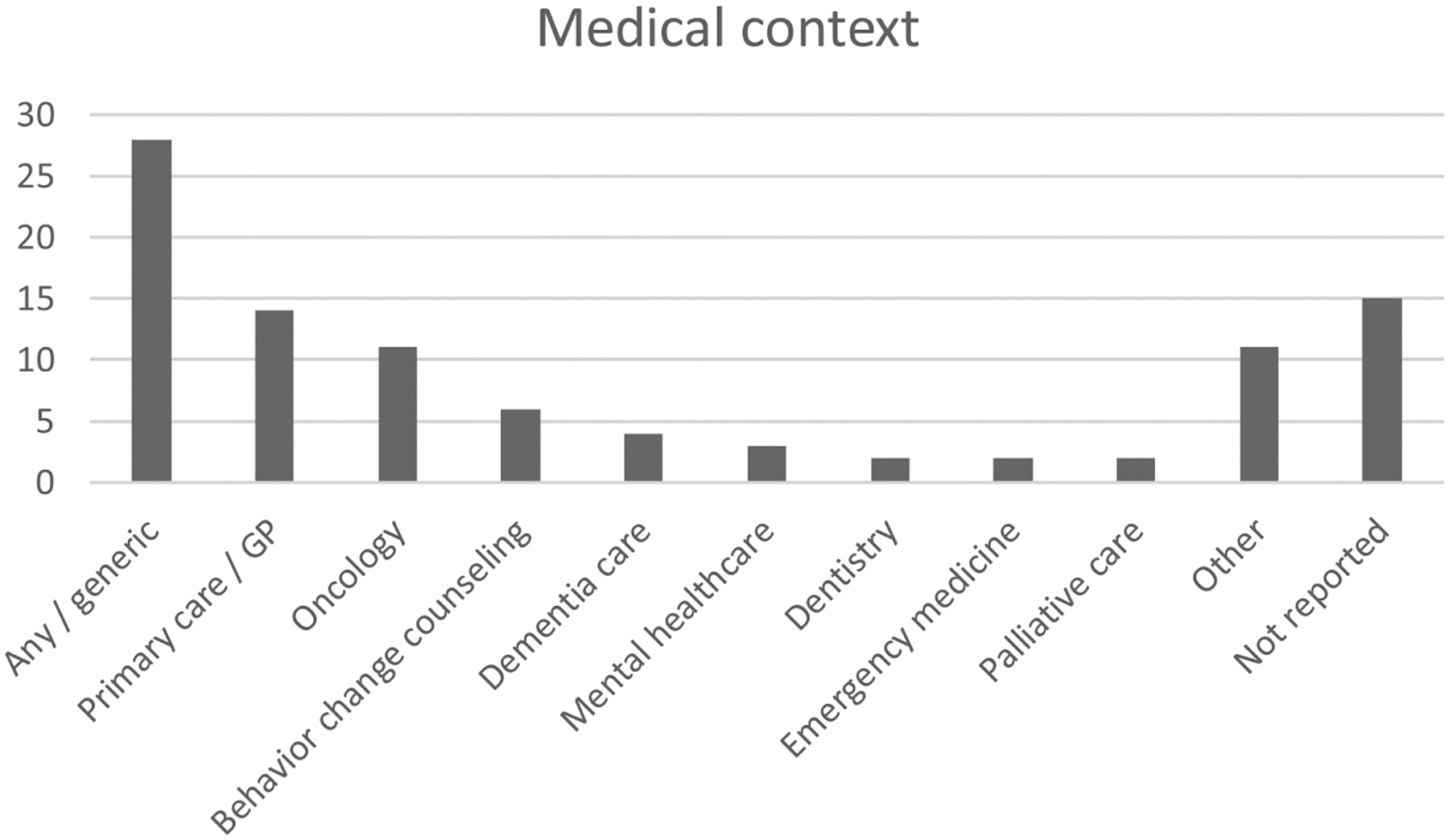
Clinical focus of the included coding systems.

The coding systems vary in the specificity of their target population, with 28 intended for generic use, i.e., any population of patients and/or healthcare professionals (see Fig. 4). Others are intended for use in specific medical contexts, most frequently primary care/general practice (n = 14), oncology (n = 11), behavioral change counseling (n = 6), dementia care (n = 4), and mental healthcare (n = 3). For 15 systems, the authors did not specify the target context. Some systems were developed specifically for application in interactions with children and/or their caregivers (n = 10) or elderly people (n = 4).

Fifty-one systems aim exclusively to assess healthcare professional behavior during interactions with patients and/or informal caregivers. Forty systems assess both patient/caregiver and health professional behavior, and six systems are intended to exclusively assess the behavior of patients and/or their informal caregivers (e.g., parents). For one coding system, behavioral focus was unclear.

#### 3.2.5. Number of items and response type

The number of items contained in the systems ranges between one and 95. Items have most often been grouped into subscales or dimensions. For 21 systems, the number of items and/or subscales was not reported or was unclear. Most frequently, the systems entail micro-level coding to yield frequencies (n = 46), i.e., aim to *assess whether, how often, at what point in the interaction and/or in what order* behaviors occur. For example, the Decision Support Analysis Tool (DSAT) codes the frequency of six categories of health professional decision support behaviors [38]. Only three coding systems explicitly assess interactional sequences, such as patient expressions and clinicians’ subsequent responses [39–41]. Fourteen systems entail macro-level evaluative assessments, i.e., to assess *the extent or quality* of observed behaviors. For example, the Behaviour Change Counselling Index (BECCI) assesses the extent and quality of professionals’ behavior change counseling behaviors using 11 items scored on a five-point Likert scale [42]. Other systems yield both frequency and evaluation-based assessment (n = 19), frequency and duration (n = 13), or frequency, evaluation and duration (n = 2). For four coding systems, it was not reported what type of data the system produces.

### 3.3. Assessment of psychometric characteristics

An overview of what psychometric properties have been assessed for each of the systems is provided in Table 2; supplementary Table 2a (Appendix D) and 2b (Appendix E) include further details.

#### 3.3.1. Sample match and coding procedures

For 75 systems, there was a clear match between the development/validation sample and the intended population as stated by the authors (Appendix D). For the remaining systems, this was unclear (n = 19), there was no match (n = 2), or this was not applicable because development did not involve an empirical sample (n = 2). Reporting about coder training and coding procedures (e.g., the proportion of double coding, methods used to reach consensus) was often missing or extremely limited. For 31 systems, no information about *coder training* was provided, and for other systems, this information was limited to the mention that coders were ‘trained’. Some articles included elaborate details about coder training. Any reporting about *coding procedures* was lacking for 15 systems. For others, descriptions varied from mentioning ‘independent coding’ through detailed explanations of all steps of the coding process. The reported *number of coders* ranged from 1 to 10, with seven systems lacking any mention about this aspect of their study procedures.

#### 3.3.2. Validity testing

Content validity was assessed for 13 out of 98 systems (unclear for 1), and structural validity for 9 systems (unclear for 1, n/a for 1). Internal consistency was assessed in 14 coding systems (not applicable for 10 systems, e.g., because it consisted of 1 item only). Other types of validity were reported for a minority of coding systems, i.e., criterion validity (n = 11), convergent validity (n = 24), discriminative validity (n = 6), and known-groups validity (n = 16). Cross-cultural adaptation to a different language, culture, or medical context was performed for nine systems, and at least some aspects of cross-cultural validity were tested for four of these.

#### 3.3.3. Reliability testing

Intra-rater reliability (i.e., consistency in coding of the same data at different time points, by the same person) was reported for 24 systems. Inter-rater reliability (i.e., consistency in coding of the same data by two or more persons) was reported by most: 89 out of 98. Note that a wide variation in methods was used to establish intra- and inter-rater reliability, for example, Kappa versus percentage agreement, and overall agreement or for specific dimensions (see Appendix D / Supplementary Table 2). Reporting about the actual method and procedures used to assess inter-rater reliability was often minimal, leaving readers in the dark about the number of consultations and the subset of items for which, and the way in which agreement had been calculated.

## 4. Discussion and conclusion

### 4.1. Discussion

In this systematic review we identified multiple coding systems that assess various aspects of the interaction between healthcare professionals and patients and/or patients’ caregivers across medical contexts. Existing coding systems vary greatly by clinical and interactional focus, coding methods and complexity, and thoroughness of development description and psychometric testing. Oftentimes the descriptions of the selected constructs, the development process, and the coding procedures were limited. Inter-rater reliability was the most consistently reported psychometric property. Most systems did not undergo further validation after initial development, and only a few were formally translated and/or cross-culturally validated. The few coding systems that have been more elaborately tested tend to be generically applicable, in terms of healthcare professionals and clinical settings.

Our finding that many coding systems lack solid evidence for their validity testing can be explained in several ways. First, interactional data collection is highly labor- and resource-intensive [43], and these efforts are difficult to justify if the only intent is coding system validation. Moreover, obtaining consent to audio- or videorecord during healthcare interactions can be difficult, as patients can feel vulnerable and emotionally distressed when asked for consent [44,45]. Healthcare professionals may decline to participate as they are concerned about their communication behavior being subjected to (negative) evaluation. Data collection usually requires researchers to make sure the recordings with consenting participants are made, collected, and the data safely filed. Therefore, the effort of collecting video or audio recordings of healthcare interactions solely for the purpose of coding system validation is often not practically feasible and difficult to justify. Second, applying observer-based coding systems is highly labor- and time-intensive [46,47]. Most systems require training, which is more or less extensive depending on coding system complexity and specificity [14]. At a minimum two coders are needed for at least part of the data to ensure reliability. Validity testing will usually require performing additional rounds of coding on the same data, and researchers may lack budget or personnel for this, especially as this research aim may not be a priority for funding bodies. Instead, authors may include the development and initial validation of a coding system as a sub-study of a funded research project that is tangential to the original research question. Third, while face validity may be fairly easy to demonstrate, assessing convergent, discriminative and/or criterion validity can be challenging. Particularly when systems focus on specific aspects of interactions, no related systems may be available to compare against the newly-developed system. Patient or professional self-report instruments with a similar focus may exist, but are not necessarily expected to correlate strongly with observer-based measures. Notwithstanding the many practical challenges to establishing validity, efforts to obtain such evidence are still crucial to the credibility and usefulness of coding systems. For systems which development is intended for wider use, particular efforts should be made to demonstrate their validity. Systems developed for one specific study only require at least some validity evidence to ensure credible results. A rigorous development process involving solid conceptualization and operationalization of the construct, embedded in prior literature and validated through expert assessment, can be expected to result in a system with sufficient validity. Subsequently, developers may assess construct validity and inter- and intra-rater reliability when using the newly-developed system. All of these validity measures can be put in place without the need of a separate validation study. Further discussion is needed to establish consensus regarding the minimal requirements for adequate development and validation of coding systems, depending on their particular purpose. Of note, even when validity evidence has been obtained for an *existing* coding system in a particular context, its validity is not ‘established’ per se. Use of such systems in different contexts or with different purposes requires further validity evidence. For example, a system assessing generic communication features and validated within a Canadian general practice setting, should be subjected to further validity testing when applied in a Brazilian oncology context. Establishing evidence for the validity of both existing and newly developed coding systems will ultimately enhance uptake of those with demonstrated validity, which in turn can inform interventions to improve communication, including teaching and training of healthcare professionals [48]. A recently published reporting guideline will facilitate system developers in reporting essential information, and assist users and reviewers in assessing the rigor of methodological reporting [14].

Strengths of this review are, first, the rigor of the search, selection and extraction process by our international team. All steps throughout this process were performed by at least two people, to ensure reliability and exhaustiveness. Second, compared to previous reviews we employed a broader focus by including any system relevant to health interactions, rather than focusing on a particular context or interactionally generic systems [19] (or specific types of (validated) systems [49]. Our broader focus enables researchers interested in specific interactional aspects or clinical contexts to identify, use, and build upon existing coding systems. Limitations of our review are, first, that not all existing coding systems may have been identified in our search. Terminology used varies, and thus not all coding systems are clearly described as such. Moreover, when both development and application of a system are described in one article, the development may not be reported in the article’s title or abstract. Future reviews on this topic might consider how to further account for the variety of language used to describe coding system development and application in their search. Second, we supplemented our search by checking a recent review on SDM measures and an online database for coding systems that we had missed. Since we did not perform a search of other recent systematic reviews focusing on specific interaction aspects, we may have failed to detect even more coding systems. Yet, considering the range of behaviors assessed in the included coding systems, we do not believe that if we had, the overall results of our review would have been different in a meaningful way. A third limitation is that we chose to exclude a system if the aim of the study was not to develop or validate a system, or when few or no details were provided about its development process. Although we made these decisions in consensus, we did not use explicit and specific decision rules, which may have resulted in a lack of systematicity. Fourth, due to imprecise reporting, it was challenging to distinguish between content validity as established during the development process (e.g., consulting experts) and validity testing of finalized coding systems.

### 4.2. Practice implications

This overview of available coding systems can aid researchers in selecting the most suitable coding system for their specific research purposes. Considering the wealth of available systems, researchers are encouraged to first determine what coding systems exist that may fit their purposes and, if appropriate, use and/or adapt existing systems rather than develop new coding systems. This may save them valuable time and research efforts and resources, although establishing validity of coding systems is still important after modification or when applied in a novel medical or geographical context. Researchers can decide on using a coding system, based on various considerations. A first consideration entails deciding whether it is (most) appropriate for their purpose to describe and/or evaluate interactions from an independent observer perspective. Second, researchers could consider whether their research question can best be answered by capturing interactions overall, or specific verbal and/or non-verbal elements within them. A third consideration is the focus of the behavior researchers want to capture, i. e., the healthcare professional, patient, and/or patient’s caregiver. Fourth, researchers could consider the specificity of their clinical focus, which may guide their choice of a context-specific or generic measure. Fifth, practical aspects and resource availability may constrain the researcher’s decision between coding systems. Thus, researchers should consider the time and coding capacity available as well as characteristics of their data (e.g., video, audio, transcripts). Such practical aspects need to be compatible with the selected coding system. For example, if video data are not available, coding many non-verbal aspects will not be feasible. Information about nearly all coding systems identified in this review has now been included in an open access, searchable database, which is openly accessible at https://each.international/projects/research/. Combined with the current review, this resource may help researchers in selecting appropriate coding systems to address their research aims. Finally, we strongly recommend that, in order to facilitate the uptake of existing coding systems, developers make their systems freely available to all researchers and provide easy access to coding manuals and training materials. The aforementioned online database includes an option to add newly developed systems.

The results of this review have the potential to facilitate the identification of future trends in the foci or specific methods used to assess healthcare interactions. For example, nascent computer-automated coding using natural language processing is a technique that has the potential to dramatically reduce the time and resources required to systematically assess interactions. With the rise of large language model-driven artificial intelligence, these techniques may be further refined and become widely available.

### 4.3. Conclusions

This systematic review identified a wide range of available coding systems to systematically assess interactions between patients, patients’ informal caregivers, and healthcare professionals, helping to raise awareness of their existence among potential users. Many systems lack thorough development and evidence of validity, and reporting of these often lacks detail. To enhance the quality of future research on healthcare interaction, more systematic reporting and psychometric testing are urgently needed. Testing may initially focus on the most relevant and broadly applicable coding systems. In the meantime, researchers seeking to quantitatively code healthcare interaction may use our overview to select systems that optimally fit their research aims.

## Supplementary Material

1

2

3

4

5

## Figures and Tables

**Table 1:** General characteristics of included coding systems (N = 98).

Instrument name (acronym)	First author	Year^a^	Validation studies	Construct	Target population	Sample(s) and size, setting	Target ‘actor’	Behaviors assessed: # items, domains (if applicable)	Response type(s)^b^
5 As for Physical Activity	Carroll [50]	2011	n/a^c^	Behavior change counseling; definition provided^d^	Patients with low physical activity and their treating clinicians	135 patients, 28 physicians; Internal/family medicine	Provider, patient	5 items	Frequency
5a-DOC	Lawson [51]	2009	n/a	Behavior change counseling; definition provided	Primary care outpatient visits and primary care clinicians	Development: 186 patients, 5 physicians; evaluation: 739 patients & 28 physicians; Primary care	Provider	5 items	Frequency
Active Patient Participation Coding (APPC) scale	Street [52]	2005	n/a	Patient participation (patients) & partnership building & supportive talk (providers); definitions provided	NR^c^	9 patients, 9 providers; medical setting NR	Provider, patient	5 items, 2 subscales: Patient participation & providers’ patient-centered communication	Frequency
Advance Care Planning Communication Assessment Tool (ACP-CAT)	Yuen [53]	2020	n/a	Advance care planning communication skills; definition provided	Clinicians and trainees conducting or learning to conduct ACP conversations	2 simulated patients, 29 residents; advanced cancer /Internal Medicine	Provider	22 items, 6 subscales (5 for ACP-steps, 1 general communication skills) and 2 summative items	Frequency, evaluation
Analysis System for Self-Efficacy Training (ASSET)	Zinken [54]	2008	n/a	Self-efficacy enhancement techniques; definition provided	Patients with Type 1 or 2 diabetes, and health professionals applying self-management interventions	12 patients, 4 providers; type 1 diabetes	Provider	13 items, 6 domains; Selfefficacy-driven verbal techniques & and patient’s response to those techniques	Frequency, duration
Assessment of Brief Behavioural Change Counselling’ tool (ABC tool)	Fouche [55]	2020	n/a	Brief behavior change counseling; definition provided	Primary care providers	Standardized patients #NR, providers #NR; primary care	Provider	22 items; 5 domains: ask, advise, assess, assist, arrange	Frequency
Behavioral Health Treatment Model for patients with Medically Unexplained Symptoms (BHTM-MUS)	Grayson-Sneed [56]	2018	n/a	Patient-centered skills and BHTM-MUS skills derived from motivational interviewing (MI); definition NR.	Patients with MUS and comorbid depression and/or opioid misuse, and medical clinicians	12 simulated patients, 161 providers; medical setting NR	Provider	33 items, 7 domains (see Supplementary Table 1)	Frequency
Behavioral Test of Interpersonal Skills (BTIS)	Olson [57]	1991	n/a	Active listening; definition provided	NR	Patients #NR, 41 providers; medical setting NR	Provider, patient	3 subscales: Feeling, Content, ‘Don’t feel’	Frequency
Behaviour Change Counselling Index (BECCI)	Lane [42]	2005	n/a	Behavior change counseling; definition provided	Patients undergoing and practitioners delivering/training for behavior change counseling	183 patients, providers #NR; 9 different samples (details NR); medical setting NR	Provider	11 items, 4 domains: agenda setting and permission seeking; the how and why of change in behavior; the consultation as a whole; talk about targets	Evaluation
Brief Decision Support Analysis Tool (DSAT–10)	Stacey [58]	2008	n/a	Decision support; definition provided	Generic	6 standardized patients, 38 providers; various medical settings	Provider	10 items, 5 elements: decision-making status, knowledge, values/preferences, others’ involvement in decision, next steps	Frequency
Cancode interaction analysis system (Cancode)	Dent [59]	2005	n/a	Cancer-specific consultation interaction; definition NR	Cancer patients and oncologists	1 simulated patient, 10 providers; oncology	Provider	#items NR, 4 domains: Source, Content, Function, Emotion & verbal/non-verbal codes	Frequency, duration
Caring Behavior Coding Scheme (CBCS)	Martensson [60]	2021	n/a	Patient and provider caring and non-caring behavior; definition provided	All patients and providers	Patients #NR, providers #NR; nursing	Provider	# items NR; Example items: Maintaining belief, knowing, being with, doing for, enabling	Frequency, duration
Chronic Pain Coding System (CPCS)	Henry [61]	2016	n/a	Patient-centered communication, negotiations; patient assessment of their physical pain and pain treatment; definition NR, operationalization provided	NR	45 patients, 33 providers; chronic pain	Provider, patient	7 major patient/companion utterance themes; 22 subscales. 6 major clinician utterance themes; 21 subscales	Frequency
Coding System for Staff-Patient Interactions (CSSPI)	Kolko [62]	1989	n/a	Adherence to contingency management procedures; definition NR	Pediatric psychiatric patients and staff	24 patients, 31 providers; mental health	Provider, patient	11 items, 3 subscales: Staff antecedent, Patient behavior, Staff consequence	Frequency, evaluation
Content Coding for Contextualization of Care (4 C)	Weiner [63]	2014	n/a	Physician attention and responsiveness to patient context when planning care; definition provided	NR	Patients #NR, providers #NR; internal medicine	Provider	# items NR, 10 contextual domains. Contextual Factors, Contextual Red Flags, Contextual Probes, Contextualized Plans of Care relating to 1 of 10 domains	Frequency
Davis Observation Code (DOC)	Callahan [64]	1991	n/a	Verbal physician behaviors relating to disease prevention, health education, health promotion, compliance checking; definition NR	Primary care patients and physicians	60 patients, 31 residents	Provider, patient	20 items	Frequency
Decision Analysis System for Oncology (DAS-O)	Brown [65]	2011	n/a	Establishing a shared decision-making framework, and providing clear and unbiased information about standard treatments and clinical trials; definition NR	Oncology patients and oncologists	143 patients, 46 providers; early-stage breast cancer	Provider, patient	56 items, 5 domains: Establishing the physician-patient team, Following a consultation pathway, Providing information about standard treatments and clinical trials, Promoting clarity, Avoiding coercion	Frequency, evaluation
Decision Support Analysis Tool (DSAT)	Guimond [38]	2003	Butow 2010 [66]	Providers’ use of decision support and related communication skills during clinical encounters; definition NR	Provider and patient making a decision (generic)	34–55 patients, 20–34 physicians; 2 different samples; various medical settings	Provider	22 items, 6 categories of decision support skills & 4 categories of communication skills	Frequency
Dementia Competent Social Interaction (DSI) and Culturally Competent Social Interaction (CSI) Coding Schemas	Kim [67]	2012	n/a	Dementia competent and culturally competent social interactions; definition NR	Korean-American older adults with dementia and direct-care staff	32 patients, 6 providers; dementia	Provider	27 items (DSI) and 17 items (CSI). For DSI: 12 Competent behaviors, 15 Incompetent behaviors. For CSI: 8 Competent behaviors, 9 Incompetent behaviors	Frequency
Detail of Essential Elements and Participants in Shared Decision Making (DEEP-SDM)	Clayman [22]	2012	n/a	Essential elements of shared decision making; definition provided	Patients presented with therapeutic choice and any health professionals presenting choice	20 patients, providers #NR; advanced breast cancer	Provider, patient	# items NR	Frequency, evaluation
Elements of Informed Decision Making (IDM)	Braddock [68]	1999	Weiss 2008 [69]; Leader 2012 [41]	Informed decision making; definition provided	Providers and patients making decisions in routine care	123–1057 patients, 12–124 providers; 3 different samples; various medical settings	Provider	7 items assessing 7 elements (e.g., Patient’s role in decision making) & 3 items assessing decision complexity (basic, intermediate, complex)	Frequency, evaluation
Empathic and Potential Empathic opportunity (E-PE-O) method	Eide [70]	2004	n/a	Empathic opportunities (EO) and potential empathic opportunities (PEO); definitions provided	Patients NR; oncology providers	39 patients, 4 providers; oncology	Provider, patient	# items NR: Empathic opportunity, Potential empathic opportunity	Frequency
Empathic Communication Coding System (ECCS)	Bylund [71]	2002	n/a	Empathic communication, specifically patients’ empathic opportunities and physicians’ responses to these empathic opportunities; definitions provided	Patients NR, physicians	100 patients, 20 providers; Internal Medicine	Provider, patient	9 items, 2 domains: Patient’s empathic opportunity, Physician empathy rating	Frequency, evaluation
eSEGUE	Assis-Hassid [72]	2015	n/a	Patient-doctor-computer communication; definition provided	Patients and providers in primary care	n/a	Provider	23 items, 5 dimensions: Set the stage, Elicit information, Give information, Understand the patient’s perspective, End the encounter	NR
Evidence-Based Patient-Centered Interviewing Method	Grayson-Sneed [73]	2017	n/a	Basic patient-centered skills, i.e. data-gathering and emotion-handling; definition NR, operationalization provided	NR	12 simulated patients, 127 providers; medical setting NR	Provider	33 items, 6 subscales: Setting the agenda, Physical story, Personal story, Emotional story, Indirect patient-centered skills, General skills	Frequency
Four Habits Coding Scheme (4-HCS)	Krupat [74]	2006	Jensen 2010 [75]; Catani 2018 [76]; Bellier 2020 [77]	Provider communication skills; definition NR	Patients and providers (not further specified)	4–497 (standardized) patients, 50–200 providers; 4 different samples; various medical settings	Provider	23 items, 4 subscales: Invest in the beginning, Elicit patient’s perspective, Demonstrate empathy, Invest in the end	Frequency, evaluation
Global Behavior Scale (GBS)	Lann-Wolcott [78]	2011	n/a	Person-centered caregiving in gerontology; definition provided	Long-term care residents with dementia and their caregivers	20 patients, 52 providers; dementia care	Provider	11 items	Evaluation
Health Coaching Index (HCI)	Sohl [79]	2021	n/a	Health coaching skills; definition provided	Patients visiting health coaches, health coaches (in training)	Standardized patients #NR, 42 providers; health coaching	Provider	18 items, 4 domains: Long-term vision, designing goals & actions, and selfmonitoring, Use a self-discovery process, Match information to client interest and needs, Have a client-centered/mindful approach	Evaluation
Health Professional-Geriatric Patient Interaction Behavior Rating Code	Adelson [31]	1982	n/a	Relational style of patients and providers; definition NR	Geriatric patients, healthcare professionals	Patients #NR, 36 providers; geriatric nursing	Provider	# items NR	Frequency
Indicators of Health Behavior Change (IHBC)	Potempa [80]	2023	n/a	Change talk, insight/pattern recognition, self-efficacy/agency, sustainability, resilience; definitions provided	NR	59 patients, providers NR; health coaching	Patient	# items NR, 5 domains: Change talk, Insight/pattern recognition, Self-efficacy and independent agency, Sustainability, Resilience	Frequency, evaluation
Kin interaction coding system (KINcode)	Laidsaar-Powell [81]	2015	n/a	Communication and decision-making behaviors; definition NR	Oncology patients and family members, oncologists	72 patients, 59 family members, 18 providers; oncology	Provider, patient, patient family	70 items, 4 domains: Family member global role, Family member’ s behaviors, Oncologist behaviors towards family member, Patient behaviors towards family member	Frequency
Liverpool-MAAS	Enzer [82]	2003	n/a	Consultation competence; definition provided	Patients visiting a general practitioner (GP) and GPs	Development: 4 patients, 4 providers; evaluation: 71 patients, 8 providers; general practice	Provider	95 items, 6 subscales: Exploring, History taking, Attitudes, Presenting solutions, Structuring interview, Interpersonal skills, Communicative skills	Evaluation
Manchester Observation Code (MOC) for genetic counseling	Liede [83]	2000	n/a	Communication behavior specific to genetic counseling interviews; definition NR	People visiting for genetic counseling, genetic counselors	38 clients/children, 39 providers; genetic counseling	Provider	20 items, 4 domains. For counselor expressions: grammatical form, Purpose, Subject, Cue source	Frequency
Mappin’SDM	Kasper [84]	2012	Kienlin 2017	Shared decision-making (SDM) behaviors and results (extent of actual involvement achieved); definitions provided except for SDM	NR	32–40 patients, 10–23 providers; 2 different samples; various medical settings	Provider, patient, dyad	15 items, each scored 3 times (for provider, patient, and dyad)	Evaluation
Measure of Patient-Centered Communication (MPCC)	Shields [85]	2005	n/a	Physicians’ use of emotion words; definition NR	NR	2 standardized patients, 50 patients, providers; primary care	Provider	# items n/a. Relative total number of emotion words spoken by the physician	Frequency
Medical Interaction Process System (MIPS)	Ford [40]	2000	Ford, 2004 [86]	Information transfer in terms of content and relationship in oncologist-cancer patient encounters; definition NR	Cancer patients, oncologists	6–20 patients, 5-#NR providers; 2 different samples; oncology/NR	Provider, patient	# items NR. 15 content categories (topic addressed), 16 dependent modes of exchange (process/function of utterance), 14 independent modes, 7 global affective (paralinguistic) categories & 7 non-verbal (kinesic) categories	Frequency, evaluation
MEDICODE	Richard [87]	2006	n/a	Content of medication discussion; definition provided	Patients visiting a general practitioner (GP), GPs	462 patients, 40 physicians; general practice	Provider, patient	40 items, 4 domains: General information, Knowledge of the drug, Discussion of prescription, Effects of the drug	Frequency
Moffitt Accrual Analysis System (MAAS)	Albrecht [88]	1999	n/a	Clinical trial accrual interactions; definition provided	Cancer patients eligible for a clinical trial, oncologists	48 patients, 12 providers; medical oncology	Provider	# items NR. Global judgements, provider responsiveness to patient concerns, MD directed toward signed consent, Conformity to legal consent form, Sharing of floor time, Amount info given, MD information orientation, opinion-based MD information orientation	Frequency, evaluation
Motivational Interviewing Skills Code (MISC)	Moyers [89]	2003	Lord 2015 [90]	Motivational Interviewing (MI) skills; definition provided	Individuals for whom behavior change is desirable, providers counseling behavioral change	Range, 19–50 patients, 4-NR providers; 2 different samples; motivational interviewing	Provider, patient	46 items, 7 domains: Global assessment of provider, Global assessment of patient, Global assessment of interaction, Therapist behaviors, Client behaviors, Therapist adherence to MI method & Talk time ratio	Frequency, evaluation, duration
Motivational Interviewing Treatment Integrity (MITI) rating system	McMaster [91]	2015	Forsberg 2007 [92]; Forsberg 2008 [93]	Motivational interviewing; definition provided	NR	6–60 (standardized) patients, range, #NR–27 providers; 3 different samples; motivational interviewing	Provider	12 items, ‘Global’ scores & Behavior counts	Frequency, evaluation
Multi-Dimensional Analysis of Patient Outcome Predictions (MD.POP)	Ménard [94]	2018	n/a	Verbal discussion of patient outcomes during medical encounters; definition for ‘patient outcomes’ provided	NR	1 simulated patient, 40 providers; oncology	Provider, patient	6 items	Frequency
Nonverbal Accommodation Analysis System (NAAS)	D’Agostino [34]	2011	n/a	Nonverbal communication in medical consultations; definition NR	Patients and physicians within medical consultations	45 patients, providers #NR; oncology	Provider, patient	10 items	Frequency, duration
Nonverbal communication in Doctor–Elderly Patient Transactions (NDEPT)	Gorawara-Bhat [35]	2007	n/a	Non-verbal communication; definition provided	Elderly patients, providers NR	50 patients, providers #NR	Provider	16 items, 3 domains: Static attributes, Dynamic attributes & Kinesic attributes	Frequency, evaluation
NR (“Strength of connectional silence” taxonomy)	Bartels [37]	2016	n/a	Silence during interactions, specifically: connectional silence, invitational silence, neutral silence, disengaged silence; definitions provided	Advanced cancer patients, oncologists	124 patients and caregivers, 41 providers; oncology	Provider	10 dimensions – 5 lexical & 5 musical	n/a
NR (ambivalence and reluctance in palliative care)	Gupta [30]	2023	n/a	Ambivalence; definition provided	Patients with serious illness, palliative care clinicians	70 patients and caregivers, 22 providers; palliative care	Patient, patient’s caregiver	3 items	Frequency
NR (Automatic annotation of information-giving in clinical conversations)	Mayfield [95]	2014	n/a	Information-giving; definition provided	Patients and providers in clinical encounters	415 patients, 45 providers; HIV	Provider, patient	3 items	Frequency
NR (behavior change therapy delivery and receipt)	Hoekstra [96]	2022	n/a	Behavior change technique delivery and receipt; definition provided	Adults with physical disabilities, counselors delivering physical activity behavior change counseling	14 patients & 1 provider; chronic spinal cord injuries	Provider, client	94 items, 13 domains (e.g., Support strategies; see Supplementary Table 1)	Frequency
NR (caregiver activation in advanced cancer care)	Dingley [97]	2017	n/a	Caregiver activation during home hospice care; definition provided	Caregivers of (cancer) patients in hospice care, hospice nurses	60 patients and caregivers; providers #NR; hospice care	Patient’s caregiver	9 items, 2 domains: Content & Processes	Frequency
NR (client responses to practitioner-delivered behavior change techniques)	Gainforth [98]	2016	n/a	Client verbal statements in response to Behavioral Change Techniques (BCT); Definition provided for ‘Client statement’	Smokers, practitioners delivering behavior support interventions for smoking cessation	18 patients, providers #NR; behavior change	Patient	58 items, 5 domains: ‘Client’ BCT, Behavior maximizing self-regulatory capacity or skills, Adjuvant activities, General aspects of the interaction & Statements unrelated to smoking or smoking cessation	Frequency
NR (coding frame for shared decision making)	Singh [99]	2010	n/a	Shared decision-making behaviors; definition provided	Cancer patients, cancer specialists	63 patients, providers NR; oncology	Provider	20 items, 6 domains: Establishing the problem, Doctor-patient relationship, Research evidence, Patient perspective, Decision making &Time issues	Evaluation
NR (coding of patient-provider self-care communication and EMR usage)	Arar [100]	2006	n/a	Self-care management practices; definition NR. EMR usage; definition provided	NR	50 patients, 6 providers; chronic illness/primary care	Provider, patient	# items NR	NR
NR (coding scheme to identify the content of general practitioner communication)	Di Caccavo [101]	2000	n/a	Content of general practitioner verbal communication behaviors; definition NR	General practice patients with psychological complaints, providers (not further specified)	75 patients, providers NR; psychological symptoms/general practice	Provider	# items NR, 16 domains (e.g., Mental symptom description; see Supplementary Table 1)	Frequency
NR (cognitive strategies used by general practitioners in diagnostic reasoning)	Donner-Banzhoff [102]	2017	n/a	Cognitive strategies, specifically: inductive foraging, descriptive questioning, triggered routines, hypothesis testing; definitions provided	Patients visiting general practitioner (GP)/primary care physicians, GPs/primary care physicians	134 patients & 12 providers; general practice	Provider, patient	# items NR	Frequency
NR (complex oral information transfer)	Nordfalk [103]	2019	n/a	Complex information transfer; definition provided	Complex information transfer situations	34 standardized patients, 17 providers; multiple sclerosis	Provider, patient	2 items/domains: Count-COPIN (units of provided information), Count-PROPIN (units of information recalled by patient)	Frequency
NR (decision making around limiting life supporting treatment in neonatal intensive care)	Shaw [104]	2023	n/a	Critical care decision making, specifically (1) the first decision point, (2) aspects of any subsequent decision point(s), and (3) how parents respond; definition NR	Parents of critically ill children in neonatal care, physicians in neonatal care	Parents of 21 infants; 14 providers; neonatal intensive care	Provider, patient’s parent(s)	22 items, 3 domains: First decision point, Subsequent decision points, Parents’ responses	Frequency, evaluation
NR (Global Consultation Rating Scale (GCRS) adapted for pediatric subspecialties)	Coburn [105]	2020	n/a	Adequate provider communication according to Cambridge-Calgary guidelines; definition provided	Patients, patients’ caregivers and providers in pediatric sub-specialties	99 patients, 18 providers; chronic kidney disease	Provider	# items NR, 12 domains (e.g., Patient’s perspective; see Supplementary Table 1)	Evaluation
NR (goal communication in palliative care decision-making consultations)	Gramling [106]	2015	n/a	Explicit verbal goal expression in palliative care consultations; Definition provided for ‘ goals’	NR	72 patients and their families, providers #NR; end-of-life care	Provider, patient	11 items, 3 domains: Patient goal expression, Provider goal expression & Other aspects of conversation	Frequency
NR (metaphor use)	Macagno [29]	2019	n/a	Use and problematic understanding of metaphors; Definitions provided for ‘Metaphors’ and ‘Problematic understanding’	Patients, healthcare providers (not further specified)	13 patients, 6 providers; diabetes	Provider, patient	# items NR	Frequency
NR (number and type of problems discussed in primary care consultations)	Procter [107]	2014	n/a	Complex consultations in general practice; definition provided	Patients visiting a general practitioner (GP), GPs	Development: 13 patients, 2 providers; reliability testing: 60 patients, 30 providers; testing: 169 patients, 30 providers; general practice	Provider, patient	27 items, 2 domains: Issues & Problems/diseases	Frequency
NR (nurse-patient touch)	Bottorff [36]	1994	n/a	Nurse-patient touch; definition NR	Patients (not further specified), nurses	8 patients, 32 providers; oncology nursing	Provider, patient	13 items, 2 domains: Interactional context, Touch event	Frequency, evaluation, duration
NR (patient active involvement in mental health interaction)	Bonfils [108]	2017	n/a	Patient involvement; definition provided	Patients with severe/chronic mental health issues, providers in mental healthcare	166 patients, 4 providers; mental health	Patient	8 items	Frequency
NR (physicians’ compliance-gaining strategies)	Smith [109]	2005	n/a	Language used by physicians to influence patients; definition provided	Patients and providers (not further specified)	37 patients, 18 providers; primary health care	Provider	57 items, 10 behavior categories (see Supplementary Table 1), 2 domains (Directness & Completeness)	Frequency
NR (planning talk within motivational interviewing)	Copeland [110]	2017	n/a	Discussion of plans and goals; Definitions provided	Clients and counselors engaging in MI for behavior change	50 clients, providers #NR; weight loss	NR	# items NR, 2 domains: Goals & Plans; Goals: 2 sub-domains (Specificity, Commitment), Plans: 3 sub-domains (Past, Continuing/hypothetical, Future) & 2 criteria (Specificity, Commitment)	Frequency, evaluation
NR (safety-netting communication behaviors in primary care encounters)	Edwards [111]	2019	n/a	Safety-netting communication behaviors; definition provided	Patients in primary care, primary care providers	32 patients, 19 providers; general practice	Provider, patient	# items NR, 4 domains: Administrative codes, Safety netting contextual codes, Safety-netting advice codes & Problem contextual codes (optional)	Frequency
NR (process and content of general practice consultations involving the prescription of antibiotic agents)	Cockburn [112]	1987	n/a	Successful consultation; definition provided	Patients with prescriptions for new courses of antibiotic agents, general practitioners	177 patients, 56 providers; primary care	Provider, patient	12 items, 3 domains: Informational aspects, Mutuality & Strategies to improve knowledge and compliance	Frequency
NR (transparency in patient-provider communication)	Robins [113]	2011	n/a	Transparency in patient-provider communication; definition provided	Primary care patients and physicians	259 patients, 33 providers; primary care	Provider	9 items, 2 domains: Process & Medical content	Frequency
NR (verbal interpersonal clinical problem-solving skills)	Pridham [114]	1980	n/a	Interactive clinical problem-solving; definition provided	Primary care patients, primary care providers (e.g. family physicians, social workers, nurses)	5 patients, 5 providers; various medical settings	Provider, patient	48 items, 16 domains & 7 Phases: Scanning, Formulating; Appraising, Developing willingness/readiness to problem solve, Planning, Implementing & Evaluating	Frequency
NR (workflow task coding scheme)	Asan [33]	2015	n/a	Interaction styles with electronic health records; definition provided	Primary care patients, physicians	18 patients, 6 providers; primary care	Provider	# items NR	Frequency, duration
Observing patient involvement in decision making (OPTION^5item^)	Elwyn [115]	2013	Barr 2015 [116]; Vortel 2016 [117]; Stubenrouch 2016 [118]; Kölker 2018 [119]; Chen 2020 [120]	Essential requirements of shared decision making when providers make an effort to involve patients in decisions. Definition NR, operationalization provided	Provider and patient making a decision (generic)	27–311 patients, 8–37 providers; 6 different samples; various settings	Provider	5 items	Evaluation
Observing patient involvement in decision making (OPTION^12item^ revised)	Elwyn [27]	2005	Elwyn 2003 [121]; Goss 2007 [122]; Weiss 2008 [69]; Butow 2010 [66]; Kasper 2011 [123]; Hirsch 2012 [124]; Keller 2013 [125]; Stubenrouch 2016 [118]	Extent to which clinicians involve patients in decision-making processes; definition NR	Provider and patient making a decision (generic)	40–235 patients, 4–37 providers; 7 different samples; various medical settings	Provider	12 items	Evaluation
ON-Code	Alby [126]	2021	n/a	Doctors’ patient-centered communication practices, in particular when there are linguistic and cultural differences between the speakers; definition NR	Cancer patients, oncologists	19 patients, 3 providers; oncology	Provider, patient, patient’s companion	15 items, 7 domains (e.g., Markers of uncertainty in doctor’ s talk; see Supplementary Table 1)	Evaluation
OnePass Coding System	McMaster [91]	2015	n/a	Motivational interviewing; definition provided	NR (tool developed to be applied in a “range of settings”)	6 simulated patients; providers #NR; behavior change	Provider	23 items, 19 questions & 4 ratios (Talk time, Reflection ratio, Open-closed ratio & Complex simple)	Evaluation
Paediatric Dental Triadic Interaction Coding Scheme (PaeD-TrICS)	Yuan [32]	2019	n/a	Verbal and non-verbal interaction within a triad of dental professional-child -parent; definition NR	Paediatric dental primare care patients and their caregivers, dental health professionals	6 standardized patients, 5 providers; dentistry	Provider, patient, patient’s parent/carer	45 items, 2 domains: Verbal dental professional/parent behaviors and Non-verbal behaviors & Child behaviors	Frequency, duration
Parental Engagement Scale (PES)	Kearney [127]	2011	n/a	Parental engagement in decision making and planning for seriously ill children during pediatric palliative care consultations. Definition provided for ‘ parental engagement’	Families of pediatric palliative patients and providers	38 patients, providers #NR; pediatric advanced care	Parent of patient	# items NR, 3 domains: Information-centered dialogue, Insightful participation & Achievement of a collaboratively agreed-upon plan	NR
Patient Involvement and Collaboration in Emergency care Teams (PIC-ET) tool	Dubois [128]	2023	n/a	Patient participation and collaboration. Definition provided for ‘patient participation’	Emergency care teams	Patients n/a, providers #NR; emergency situations	Provider	22 items, 5 domains: Relationship, Sharing power, Information exchange, Safe and caring environment & Social circumstances	Frequency, evaluation
Patient-centered Behaviour Coding Instrument (PCBI)	Zandbelt [20]	2005	n/a	Patient-centered behavior; definition provided	NR	323 patients, 15 providers; internal medicine	Provider	19 items, 2 domains: Facilitating behaviors & Inhibiting behaviors; Subject area of each expression also coded	Frequency
Patient-Centered Communication Coding System (PCCCS)	Ledoux [129]	2013	n/a	Patient-centered communication (PCC); definition provided	Children/parents dealing with pediatric obesity, their providers/health advisors	30 children and parents, 5 providers; childhood obesity	Provider	16 items, 3 domains: Positive PCC, Negative PCC & Overall communication style	Frequency, evaluation
Pediatric Emergency Discharge Interaction Coding Scheme (PEDICS)	Curran [130]	2017	n/a	Discharge communication; definition provided	Parents of children visiting the emergency department, pediatric emergency department physicians and nurses	329 children and parents, 17 providers; emergency medicine	Provider	41 items, 11 domains: Introduction, Tests, Medications given in ED, Discharge, Diagnosis, Treatment plans, Medication for home, Social concerns, Follow-up, Clarification & Conclusion	Frequency, duration
Pediatric Oncology Prognostic Communication (POPC) codebook	Kaye [131]	2021	n/a	Communication strategies to share prognostic information; definition NR	Children with cancer, pediatric oncologists	17 children and parents, 6 providers; pediatric oncology	Provider	6 items	Frequency, duration
Perioperative Child–Adult Medical Procedure Interaction Scale (P-CAMPIS)	Caldwell-Andrews [132]	2005	n/a	Verbal and nonverbal interactions between children, parents, and medical personnel in the perioperative setting; definition NR	Children undergoing anesthesia, providers working in the perioperative environment	45 patients and parents, providers NR; pediatric surgery	Provider, patient, patient’s parent	40 items, 4 domains: Adult-to-adult communication, Adult-child communication, Either, Child vocalizations & Nonverbal behavior	Frequency
Person-Centered Behavior Inventory (PCBI)	Lann-Wolcott [78]	2011	n/a	Person-centered caregiving in gerontology; definition provided	Long-term care residents with dementia, their caregivers	20 patients, 52 providers; dementia care	Provider	19 items, 2 domains: Verbal categories & Nonverbal categories	Frequency, duration
Physician Recommendation Coding System (PhyReCS)	Scherr [133]	2017	n/a	The strength of physician recommendations during appointments; definition provided	Patients confronted with and providers involving patients in a preference-sensitive treatment choice	257 patients, 47 providers; prostate cancer screening	Provider	1 item	Evaluation
Problem, Analysis or Assessment, and Solution (Pas^®^ System)	Van Mil [21]	1997	n/a	Patient questions about medication and pharmacist assessment of question and solution offered; definition NR	Patients asking questions to pharmacists about medication, pharmacists responding to these questions	n/a	Provider, patient	15 items, 3 domains: Patient questions, Provider analysis/assessment of the nature of the question & Provider solution offered	Frequency
Process of Interactional Sensitivity Coding in Healthcare (PISCH)	Sabee [134]	2015	n/a	Interactional Sensitivity; definition provided	Patients with type 2 diabetes/patients in general, physicians treating these patients	52 patients, 52 providers; various medical settings	Provider, patient	# items NR	Frequency
Refined Cue Utilization and Engagement in Dementia (CUeD)	Liu [135]	2020	n/a	Dyadic behaviors during mealtime interactions; definition provided for ‘intake episode’	Dementia patients living in a nursing home, nursing home staff	25 patients, 29 providers; dementia care	Provider, patient	# items NR, 4 domains: Resident intake process, Caregiver & resident verbal behaviors, Caregiver nonverbal behaviors & Resident nonverbal behaviors	Frequency, duration
Resident-centered Assessment of Interactions with Staff and Engagement tool (RAISE)	Snow [136]	2018	n/a	Person-centered care in staff-resident interactions; Definitions provided for ‘Interaction’, ‘Emotional tone’, and ‘ PCC best clinical practices’	Aged Care Patients and staff	Patients #NR, providers #NR; aged care	Provider	# items NR	Frequency, evaluation
Rochester Participatory Decision-Making Scale (RPAD)	Shields [137]	2005	Guo 2020	Participatory decision making; definition provided	NR	5–200 (standardized) patients, 10–100 providers; 2 different samples; various medical settings	Provider	9 items	Evaluation
Roter Interaction Analysis System (RIAS)	Roter [138]	2002	n/a	Dynamics of resource exchange between patients and providers through the medical dialogue; definition NR, operationalization provided	Generic	NR	Provider, patient	40 items	Frequency, duration
SDM–18	Salyers [139]	2012	n/a	Shared decision-making; definition provided	Psychiatric patients and providers	186 patients, 8 providers; mental healthcare	Provider, patient	15 items, 3 domains: Basic decisions, Intermediate decisions & Complex decisions	Frequency, evaluation
Segue framework	Makoul [140]	2001	n/a	Medical communication tasks; definition NR	NR; For providers: ‘ Different doctors in different settings’	16–46 patients, range, 16–46 providers; 2 different samples; various medical settings	Provider	25 items & 7 optional items, 2 domains: Content & Form, and 5 sub-domains: Set the stage, Elicit information, Give information, Understand the patient’s perspective & End the encounter	Frequency
Siminoff Communication Content & Affect Program (SCCAP)	Siminoff [141]	2011	n/a	Instrumental and Relational communication; definitions provided for ‘Communication content’ and ‘Relational communication’	NR, but instruments reported to be ‘adaptive to multiple health communication contexts’	180–1200 patients and/or family members, #NR–39 providers; 3 different samples; oncology	Provider	29 items, 3 domains: Verbal communication types, Verbal content themes & Non-verbal expressions of speech and affect	Frequency, evaluation
St Andrews Behavioural Interaction Coding Scheme (SABICS)	Zhou [142]	2012	n/a	Nurse-child interaction in terms of encouraging cooperative child behavior; definition provided	Children aged 3–5 in a community-based dental context, dental nurses	36–55 patients, 6–19 providers; 2 different samples; dentistry	Provider, patient	48 items, 3 domains: Protocol behaviors, Nurse behavior & Child behavior	Frequency, duration
State Space Grids	Gainforth [143]	2019	n/a	Behavioral support in provider-patient interaction; definition provided	Clients in smoking cessation behavioral support interactions, behavioral support practitioners	6 patients, 2 providers; behavior change	Provider, patient	# items NR, 2 domains: Practitioner verbal behavior & Client statements	Frequency, duration
Taxonomy of Verbal Response Modes (VRM)	Stiles [144]	1979	Stiles 1982 [145]	Verbal interactions/exchanges; definition NR (reported elsewhere)	Patients in medical interviews, providers conducting medical interviews	52–115 patients, 14–19 providers; 2 different samples; various medical settings	Provider, patient	8 items, coding of both grammatical form and intended function	Frequency, evaluation
Verona coding definitions of emotional sequences (VR-CoDES)	Zimmermann [28]	2011	Vatne 2010 [146]; Eide 2011 [147]; Wright 2012 [148]; Yin 2019 [149]; Vijfhuizen 2017 [150]	Provider responses to explicit (concern) or subtle (cue) expression of negative emotion; definitions provided for ‘Concern’ and ‘Cue’	Patients and providers (not further specified)	2–20 (simulated) patients, #NR–75 providers; 6 different samples; various medical settings	Provider	8 items, 2 domains: Cues & Concerns	Frequency
Verona Coding Definitions of Emotional Sequences for health provider responses (VR-CoDES-P)	Del Piccolo [39]	2011	Wright 2012 [148]; Vijfhuizen 2017 [150]; Yin 2019 [149]	Provider responses to explicit (concern) or subtle (cue) expression of negative emotion; definitions provided for ‘ Concern’ and ‘ Cue’	NR	2–104 (simulated) patients/clients, 2–75 providers; 4 different samples; various medical settings	Provider	17 items, 2 dimensions: Explicit/Non-explicit & Providing/reducing space	Frequency
Verona Medical Interview Classification System (VR-MICS)	Del Piccolo [151]	2004	Del Piccolo 2005 [152]	Provider responses to explicit (concern) or subtle (cue) expression of negative emotion; definitions provided for ‘ Concern’ and ‘ Cue’	Emotionally distressed patients with medical problem, physicians	#NR–238 patients, range, 6–12 providers; 2 different samples; general practice	Provider, patient	18 items, 8 domains (e.g., Relationship building; see Supplementary Table 1)	Frequency
Vr-COPE	McDermott [153]	2021	n/a	Relationship-centered communication; definition provided	Veterinary surgeons and their clients	55 clients, providers #NR; veterinary care	Provider	10 items	Evaluation

Notes:

a Year published for initial development/validation paper;

b Frequency, evaluation and/or duration - whereby frequency can refer to presence/absence, amount of occurrences, timing, and/or sequence of behaviors; evaluation can refer to the extent and/or quality of an observed behavior; and duration to the length of time during which a specific behavior occurs;

c n/a, not applicable; NR, Not Reported; #, number;

d Definitions –if provided– are displayed in Appendix B/ Supplementary Table 1b.

**Table 2:** Extent of psychometric evaluation of the coding systems (N = 98).

Instrument name (Acronym)	First author	Year	Content validity	Structural validity	Internal consistency	Cross-cultural adaptation	Cross-cultural validity	Intra-rater reliability	Inter-rater reliability	Criterion validity	Convergent validity	Discriminative validity	Known-groups validity
5 As for Physical Activity	Carroll	2011	–	–	–	n/a	n/a	–	•	–	–	–	–
5a-DOC	Lawson	2009	–	–	–	n/a	n/a	–	•	–	–	–	–
Active Patient Participation Coding (APPC) scale	Street	2005	–	–	–	n/a	n/a	–	•	–	–	–	–
Advance Care Planning Communication Assessment Tool (ACP-CAT)	Yuen	2020	–	–	–	n/a	n/a	–	•	•	–	–	•
Analysis System for Self-Efficacy Training (ASSET)	Zinken	2008	–	–	–	n/a	n/a	–	•	–	–	•	–
Assessment of Brief Behavioural Change Counselling’ tool (ABC tool)	Fouche	2020	–	–	•	n/a	n/a	•	•	–	–	–	–
Behavioral Health Treatment Model for patients with Medically Unexplained Symptoms (BHTM-MUS)	Grayson-Sneed	2018	–	–	n/a	n/a	n/a	–	•	–	–	–	–
Behavioral Test of Interpersonal Skills (BTIS)	Olson	1991	•	–	n/a	n/a	n/a	•	•	–	•	–	–
Behaviour Change Counselling Index (BECCI)	Lane	2005	•	–	•	n/a	n/a	•	•	–	–	–	–
Brief Decision Support Analysis Tool (DSAT–10)	Stacey	2008	–	–	–	n/a	n/a	–	•	–	–	–	–
Cancode interaction analysis system (Cancode)	Dent	2005	–	•	–	n/a	n/a	•	•	–	–	–	•
Caring Behavior Coding Scheme (CBCS)	Martensson	2021	•	–	–	n/a	n/a	–	•	–	–	–	–
Chronic Pain Coding System (CPCS)	Henry	2016	–	–	•	n/a	n/a	–	•	–	•	–	–
Coding System for Staff-Patient Interactions (CSSPI)	Kolko	1989	–	–	–	n/a	n/a	–	•	–	–	–	–
Content coding for contextualization of care (4 C)	Weiner	2014	–	–	–	n/a	n/a	–	•	–	–	–	–
Davis Observation Code (DOC)	Callahan	1991	–	–	–	n/a	n/a	–	•	–	•	–	–
Decision Analysis System for Oncology (DAS-O)	Brown	2011	•	–	–	n/a	n/a	•	•	–	•	–	–
Decision Support Analysis Tool (DSAT)	Guimond	2003	–	–	–	n/a	n/a	–	•	–	•	–	•
Dementia Competent Social Interaction (DSI) and Culturally Competent Social Interaction (CSI) Coding Schemas	Kim	2012	•	–	–	n/a	n/a	–	•	–	–	–	–
Detail of essential elements and participants in shared decision making (DEEP-SDM)	Clayman	2012	–	–	–	n/a	n/a	–	–	–	–	–	–
Elements of Informed Decision Making (IDM)	Braddock	1999	–	–	•	n/a	n/a	•	•	–	•	–	–
Empathic and Potential Empathic opportunity (E-PE-O) method	Eide	2004	–	–	–	n/a	n/a	–	•	•	–	–	–
Empathic Communication Coding System (ECCS)	Bylund	2002	–	–	–	n/a	n/a	–	•	–	–	–	•
eSEGUE	Assis-Hassid	2015	•	–	–	n/a	n/a	–	–	–	–	–	–
Evidence-Based Patient-Centered Interviewing Method	Grayson-Sneed	2017	–	–	n/a	n/a	n/a	–	•	–	–	–	–
Four Habits Coding Scheme (4-HCS)	Krupat	2006	•	–	•	•	•	•	•	–	•	–	•
Global Behavior Scale (GBS)	Lann-Wolcott	2011	–	–	•	n/a	n/a	–	–	–	•	•	–
Health Coaching Index (HCI)	Sohl	2021	•	–	–	n/a	n/a	–	•	–	•	–	–
Health Professional-Geriatric Patient Interaction Behavior Rating Code	Adelson	1982	–	–	•	n/a	n/a	–	•	•	–	–	–
Indicators of Health Behavior Change (IHBC)	Potempa	2023	•	•	–	n/a	n/a	–	•	–	–	–	–
Kin interaction coding system (KINcode)	Laidsaar-Powell	2015	•	–	–	n/a	n/a	•	•	–	–	–	–
Liverpool-MAAS (LIV-MAAS)	Enzer	2003	–	–	–	n/a	n/a	–	•	–	–	–	–
Manchester Observation Code (MOC) for genetic counseling	Liede	2000	–	–	–	n/a	n/a	•	•	–	–	–	–
Mappin’SDM	Kasper	2012	–	–	–	•	–	–	•	•	•	–	–
Measure of Patient-Centered Communication (MPCC)	Shields	2005	–	–	–	n/a	n/a	–	•	•	•	–	–
Medical Interaction Process System (MIPS)	Ford	2000	–	–	–	n/a	n/a	–	•	–	•	–	•
MEDICODE	Richard	2006	–	–	n/a	n/a	n/a	•	•	–	•	–	–
Moffitt Accrual Analysis System (MAAS)	Albrecht	1999	–	–	–	n/a	n/a	-	•	–	•	•	–
Motivational Interviewing Skills Code (MISC)	Moyers	2003	–	–	–	n/a	n/a	•	•	–	-	–	–
Motivational Interviewing Treatment Integrity (MITI) rating system	McMaster	2015	–	•	–	•	–	–	•	–		–	–
Multi-Dimensional Analysis of Patient Outcome Predictions (MD.POP)	Ménard	2018	–	–	–	n/a	n/a	–	•	–		–	–
Nonverbal Accommodation Analysis System (NAAS)	D’Agostino	2011	–	–	n/a	n/a	n/a	•	•	–	•	–	–
Nonverbal communication in doctor-elderly patient transactions (NDEPT)	Gorawara-Bhat	2007	–	–	–	n/a	n/a	–	•	–	–	–	–
NR (“Strength of connectional silence” taxonomy)	Bartels	2016	–	–	–	n/a	n/a	–	•	–	–	–	–
NR (ambivalence and reluctance in palliative care)	Gupta	2023	–	–	n/a	n/a	n/a	–	•	•	–	–	–
NR (Automatic annotation of information-giving in clinical conversations)	Mayfield	2014	–	–	–	n/a	n/a	–	•	–	–	•	–
NR (behavior change therapy delivery and receipt)	Hoekstra	2022	–	–	–	n/a	n/a	–	•	–	–	–	–
NR (Caregiver Activation in Advanced Cancer Care)	Dingley	2017	–	–	–	•	–	–	–	–	–	–	–
NR (Characterize client responses to practitioner-delivered behaviour change techniques)	Gainforth	2016	–	–	–	n/a	n/a	–	•	–	–	–	–
NR (coding frame for shared decision-making)	Singh	2010	–	•	•	n/a	n/a	•	•	–	•	–	–
NR (coding of Patient-provider self-care communication and EMR usage)	Arar	2006	–	–	–	n/a	n/a	•	•	–	–	–	–
NR (coding scheme to identify the content of general practitioner communication)	Di Caccavo	2000	–	–	–	n/a	n/a	–	•	–	–	–	–
NR (Cognitive strategies used by General Practitioners in diagnostic reasoning)	Donner-Banzhoff	2017	–	–	–	n/a	n/a	–	•	–	–	–	–
NR (Complex oral information transfer)	Nordfalk	2019	–	–	–	n/a	n/a	–	•	–	–	–	–
NR (Decision-making around limiting life supporting treatment in neonatal intensive care)	Shaw	2023	–	–	•	n/a	n/a	–	•	–	–	–	–
NR (Global Consultation Rating Scale (GCRS) adapted for pediatric subspecialties)	Coburn	2020	–	–	–	n/a	n/a	–	•	–	–	–	–
NR (Goal communication in palliative care decision-making consultations)	Gramling	2015	–	–	–	n/a	n/a	–	•	–	–	–	–
NR (Metaphor use)	Macagno	2019	–	–	–	n/a	n/a	–	•	–	–	–	–
NR (number and type of problems discussed in primary care consultations)	Procter	2014	–	–	n/a	n/a	n/a	–	•	–	–	–	–
NR (Nurse Patient Touch)	Bottorff	1994	–	–	–	n/a	n/a	•	•	–	–	–	•
NR (Patient active involvement in mental health interaction)	Bonfils	2017	–	–	•	n/a	n/a	–	•	–	•	–	–
NR (physicians’ compliance-gaining strategies)	Smith	2005	–	–	–	n/a	n/a	–	•	–	–	–	–
NR (Planning talk within motivational interviewing)	Copeland	2017	–	–	–	n/a	n/a	–	•	–	–	–	–
NR (Safety-netting communication behaviors in primary care encounters)	Edwards	2019	–	–	–	n/a	n/a	–	•	–	–	–	–
NR (the process and content of general practice consultations involving the prescription of antibiotic agents)	Cockburn	1987	–	–	–	n/a	n/a	–	•	–	–	–	–
NR (transparency in patient-provider communication)	Robins	2011	–	–	–	n/a	n/a	–	•	–	–	–	–
NR (Verbal interpersonal clinical problem-solving skills)	Pridham	1980	–	–	–	n/a	n/a	–	•	–	–	–	•
NR (Workflow task coding scheme)	Asan	2015	–	–	–	n/a	n/a	–	•	–	–	–	–
Observing patient involvement in decision making (OPTION5item)	Elwyn	2013	–	–	•	•	–	•	•	–	•	–	•
Observing patient involvement in decision making (OPTION12item revised)	Elwyn	2005	–	•	•	•	–	•	•	–	•	–	•
ON-Code	Alby	2021	–	–	–	n/a	n/a	–	•	–	•	–	•
OnePass coding system	McMaster	2015	–	–	–	n/a	n/a	–	•	•	–	–	–
Paediatric Dental Triadic Interaction Coding Scheme (PaeD-TrICS)	Yuan	2019	–	–	n/a	n/a	n/a	•	•	–	–	–	–
Parental Engagement Scale (PES)	Kearney	2011	–	–	–	n/a	n/a	–	•	-	-	-	-
Patient Involvement and Collaboration in Emergency care Teams (PIC-ET) tool	Dubois	2023	•	–	–	n/a	n/a	–	•	–	–	–	–
Patient-Centered Behaviour Coding Instrument (PCBI)	Zandbelt	2005	-	•	•	n/a	n/a	–	•	•	-	-	-
Patient-Centered Communication Coding System (PCCCS)	Ledoux	2013	–	–	–	n/a	n/a	•	•	–	–	–	–
Pediatric Emergency Discharge Interaction Coding Scheme (PEDICS)	Curran	2017	–	–	–	n/a	n/a	–	•	–	–	–	–
Pediatric Oncology Prognostic Communication (POPC) codebook	Kaye	2023	–	–	–	n/a	n/a	–	–	–	–	–	–
Perioperative Child-Adult Medical Procedure Interaction Scale (P-CAMPIS)	Caldwell-Andrews	2005	–	–	–	n/a	n/a	–	•	–	•	•	–
Person Centered Behavior Inventory (PCBI)	Lann-Wolcott	2011	–	–	–	n/a	n/a	–	–	–	•	•	-
Physician Recommendation Coding System (PhyReCS)	Scherr	2017	–	n/a	n/a	n/a	n/a	–	•	•	–	–	•
Problem, Analysis or Assessment, and Solution (Pas^®^ System)	Van Mil	1997	–	–	–	n/a	n/a	–	–	–	–	–	–
Process of Interactional Sensitivity Coding in Healthcare (PISCH)	Sabee	2015	–	–	–	n/a	n/a	–	•	–	–	–	–
Refined Cue Utilization and Engagement in Dementia (CUeD)	Liu	2020	–	–	–	n/a	n/a	–	•	–	–	–	–
Resident-centered Assessment of Interactions with Staff and Engagement tool (RAISE)	Snow	2018	•	–	–	n/a	n/a	–	•	–	–	–	–
Rochester Participatory Decision-Making Scale (RPAD)	Shields	2005	–	•	•	•	–	•	–	•	•	–	–
Roter Interaction Analysis System (RIAS)*	Roter	2002	?	?	?	n/a	n/a	–	•	–	–	–	•
SDM–18	Salyers	2012	–	–	–	n/a	n/a	–	•	–	–	–	–
Segue framework	Makoul	2001	•	–	n/a	n/a	n/a	•	•	•	•	–	–
Siminoff Communication Content & Affect Program (SCCAP)	Siminoff	2011	–	–	–	n/a	n/a	–	•	–	–	–	•
St Andrews Behavioural Interaction Coding Scheme (SABICS)	Zhou	2012	–	–	–	n/a	n/a	•	•	–	–	–	•
State Space Grids	Gainforth	2019	–	–	–	n/a	n/a	–	•	–	–	–	–
Taxonomy of Verbal Response Modes (VRM)	Stiles	1979	–	•	–	n/a	n/a	–	•	–	–	–	–
Verona coding definitions of emotional sequences (VR-CoDES)	Zimmermann	2011	–	–	–	•	•	•	•	•	–	–	–
Verona Coding Definitions of Emotional Sequences for health provider responses (VR-CoDES-P)	Del Piccolo	2011	–	–	–	•	•	•	•	–	–	–	–
Verona Medical Interview Classification System (VR-MICS)	Del Piccolo	2004	–	•	–	n/a	n/a	–	•	–	–	–	–
Vr-COPE	McDermott	2021	–	–	–	n/a	n/a	–	–	–	–	–	–

Notes. •, assessed; –, not assessed; n/a, not applicable

* The original publication could not be retrieved. Information was based on Roter et al. (2002) [138] and Ong et al. (1998) [154]

## Appendix A. Supporting information

Supplementary data associated with this article can be found in the online version at doi:10.1016/j.pec.2025.108718.
