# Supplementary material for: Systematic review of observational coding systems to assess patient-healthcare professional interactions
Source: Patient Educ Couns. Author manuscript; Available in PMC 2026 Aug 16. (PMC13477582; doi:10.1016/j.pec.2025.108718)
Supplement: Supplementary file 1 [file NIHMS2172109-supplement-1.docx]

Appendix A: Full search Strategies

| Medline (Ovid) | |
| --- | --- |
| 1 | (Development* or validation* or construct valid* or cultur* adaptation* or cultur* valid* or cross-cultur* valid* or content valid* or reproducibility of results or reproducib* or psychometrics or psychometr* or observer variation or observer variation or discriminant analysis or reliab* or valid* or coefficient or internal consistency or agreement or test retest or stability or interrater or inter-rater or intrarater or intra-rater or intertester or inter-tester or intratester or intra-tester or interobserver or inter-observer or intraobserver or intra-observer or interexaminer or inter-examiner or intraexaminer or intra-examiner or interindividual or inter-individual or intraindividual or intra-individual or kappa or kappas or coefficient of variation or repeatab* or generaliza* or generalisa* or concordance or discriminative or known group or factor analysis or factor analyses or dimensionality or subscale* or item discriminant or interscale correlation or interscale correlations or individual variability or interpretab* or ceiling effect or floor effect or cross-cultural equivalence or test-retest).ti,ab,kw. |
| 2 | exp Observer Variation/ or exp Validation Study/ or exp "Predictive Value of Tests"/ or ((replicab* or repeated) adj3 (measure or measures or findings or result or results or test or tests)).mp. or (intraclass adj3 correlation*).mp. or ((error or errors) adj3 (measure* or correlat* or evaluat* or accuracy or accurate or precision or mean)).mp. or (limit adj3 detection).mp. or (small* adj3 (real or detectable) adj3 (change or difference)).mp. or (cronbach* adj3 (alpha or alphas)).mp. or (test adj3 retest).mp. or (reliab* adj3 (test or retest)).mp. |
| 3 | 1 or 2 |
| 4 | ((coding or code or coded) adj3 (method or content or scheme or definitions or framework or tool or scale or measure or instrument or procedure or process or data or measure or form or system)).mp. or (coding or code).ti. or (interview rating or discourse analysis or content analysis).kw. |
| 5 | (Provider* or clinician* or doctor* or oncologist* or cardiologist* or surgeon* or generalist* or obstetrician* or gynecologist* or physician assistant* or advanced practice nurse* or nurse* or nurse anthesthsiologist* or nurse practitioner* anesthesiologist* or midwif* or midwives* or pharmacist* or endocrinologist* or allergist* or dermatologist* or gastroenterologist* or hematologist* or immunologist* or nephrologist* or neurologist* or opthalmologist* or otorhinolaryngologist* or physical therapist* or psychiatrist* or pulmonologist* or radiologist* or rheumatologist* or urologist* or healthcare provider* or healthcare professional* or healthcare practitioner* or healthcare personnel or healthcare specialist* or health provider* or health professional* or health practitioner* or health personnel or health specialist* or medical provider* or medical professional* or medical practitioner* or medical personnel or medical specialist* or primary care provider* or primary care professional* or primary care practitioner* or primary care specialist* or primary care physician* or family provider* or family clinician* or family doctor* or family physician* or gynaecologist* or veterinarian or dentist* or general practitioner or general practice doctor or pediatrician or palliative care physician or (patient* or client* or inpatient* or outpatient* or person* or spokesperson* or spouse* or advocate* or companion* or parent* or interpreter* or caretaker* or caregiver* or navigator* or patient* spokesperson* or patient* spouse* or patient* advocat* or patient* companion* or patient* parent* or patient* interpreter* or patient* caretaker* or patient* caregiver* or patient* navigator* or family* or family member* or significant other)).ti,ab,kw. |
| 6 | (Communicat* or interact* or verbal behaviour* or verbal behavior* or verbal interaction* or non-verbal behaviour* or non-verbal behavior* or non-verbal interaction* or interpersonal behavior* or interpersonal behaviour* or interpersonal skill* or participa* behavior* or body language* or patient provider interaction* or verbal communication* or non-verbal communication* or participation* or patient-centered interviewing* or verbal response* or non-verbal response* or emotional sequence* or information-sharing* or disclosure* or patient* interviewing or relationship building or empathy or decision making or shared decision making or patient involvement or consult*).ti,ab,kw. |
| 7 | 3 and 4 and 5 and 6 |

| Embase (Ovid) | |
| --- | --- |
| 1 | (Development* or validation* or construct valid* or cultur* adaptation* or cultur* valid* or cross-cultur* valid* or content valid* or reproducibility of results or reproducib* or psychometrics or psychometr* or observer variation or observer variation or discriminant analysis or reliab* or valid* or coefficient or internal consistency or agreement or test retest or stability or interrater or inter-rater or intrarater or intra-rater or intertester or inter-tester or intratester or intra-tester or interobserver or inter-observer or intraobserver or intra-observer or interexaminer or inter-examiner or intraexaminer or intra-examiner or interindividual or inter-individual or intraindividual or intra-individual or kappa or kappas or coefficient of variation or repeatab* or generaliza* or generalisa* or concordance or discriminative or known group or factor analysis or factor analyses or dimensionality or subscale* or item discriminant or interscale correlation or interscale correlations or individual variability or interpretab* or ceiling effect or floor effect or cross-cultural equivalence or test-retest).ti,ab,kw. or exp observer variation/ or exp validation study/ or exp predictive value/ or ((replicab* or repeated) adj3 (measure or measures or findings or result or results or test or tests)).ti,ab,kw. or (intraclass adj3 correlation*).ti,ab,kw. or ((error or errors) adj3 (measure* or correlat* or evaluat* or accuracy or accurate or precision or mean)).ti,ab,kw. or (limit adj3 detection).ti,ab,kw. or (small* adj3 (real or detectable) adj3 (change or difference)).ti,ab,kw. or (cronbach* adj3 (alpha or alphas)).ti,ab,kw. or (test adj3 retest).ti,ab,kw. or (reliab* adj3 (test or retest)).ti,ab,kw. |
| 2 | ((coding or code or coded) adj3 (method or content or scheme or definitions or framework or tool or scale or measure or instrument or procedure or process or data or measure or form or system)).mp. or (coding or code).ti. or (interview rating or discourse analysis or content analysis).kw. |
| 3 | (Provider* or clinician* or doctor* or oncologist* or cardiologist* or surgeon* or generalist* or obstetrician* or gynecologist* or physician assistant* or advanced practice nurse* or nurse* or nurse anthesthsiologist* or nurse practitioner* anesthesiologist* or midwif* or midwives* or pharmacist* or endocrinologist* or allergist* or dermatologist* or gastroenterologist* or hematologist* or immunologist* or nephrologist* or neurologist* or opthalmologist* or otorhinolaryngologist* or physical therapist* or psychiatrist* or pulmonologist* or radiologist* or rheumatologist* or urologist* or healthcare provider* or healthcare professional* or healthcare practitioner* or healthcare personnel or healthcare specialist* or health provider* or health professional* or health practitioner* or health personnel or health specialist* or medical provider* or medical professional* or medical practitioner* or medical personnel or medical specialist* or primary care provider* or primary care professional* or primary care practitioner* or primary care specialist* or primary care physician* or family provider* or family clinician* or family doctor* or family physician* or gynaecologist* or veterinarian or dentist* or general practitioner or general practice doctor or pediatrician or palliative care physician or (patient* or client* or inpatient* or outpatient* or person* or spokesperson* or spouse* or advocate* or companion* or parent* or interpreter* or caretaker* or caregiver* or navigator* or patient* spokesperson* or patient* spouse* or patient* advocat* or patient* companion* or patient* parent* or patient* interpreter* or patient* caretaker* or patient* caregiver* or patient* navigator* or family* or family member* or significant other)).ti,ab,kw. |
| 4 | (Communicat* or interact* or verbal behaviour* or verbal behavior* or verbal interaction* or non-verbal behaviour* or non-verbal behavior* or non-verbal interaction* or interpersonal behavior* or interpersonal behaviour* or interpersonal skill* or participa* behavior* or body language* or patient provider interaction* or verbal communication* or non-verbal communication* or participation* or patient-centered interviewing* or verbal response* or non-verbal response* or emotional sequence* or information-sharing* or disclosure* or patient* interviewing or relationship building or empathy or decision making or shared decision making or patient involvement or consult*).ti,ab,kw. |
| 5 | 1 and 2 and 3 and 4 |

| Cochrane Library (Wiley) | |
| --- | --- |
| #1 | [mh "Observer Variation"] or [mh "Validation Study"] or [mh "Predictive Value of Tests"] or Development* or validation* or construct valid* or cultur* adaptation* or cultur* valid* or cross-cultur* valid* or content valid* or reproducibility of results or reproducib* or psychometrics or psychometr* or observer variation or observer variation or discriminant analysis or reliab* or valid* or coefficient or internal consistency or agreement or test retest or stability or interrater or inter-rater or intrarater or intra-rater or intertester or inter-tester or intratester or intra-tester or interobserver or inter-observer or intraobserver or intra-observer or interexaminer or inter-examiner or intraexaminer or intra-examiner or interindividual or inter-individual or intraindividual or intra-individual or kappa or kappas or coefficient of variation or repeatab* or generaliza* or generalisa* or concordance or discriminative or known group or factor analysis or factor analyses or dimensionality or subscale* or item discriminant or interscale correlation or interscale correlations or individual variability or interpretab* or ceiling effect or floor effect or cross-cultural equivalence or test-retest |
| #2 | ((replicab* or repeated) NEAR/3 (measure or measures or findings or result or results or test or tests)) |
| #3 | (intraclass NEAR/3 correlation*) |
| #4 | ((error or errors) NEAR/3 (measure* or correlat* or evaluat* or accuracy or accurate or precision or mean)) |
| #5 | (limit NEAR/3 detection) |
| #6 | (small* NEAR/3 (real or detectable) NEAR/3 (change or difference)) |
| #7 | (cronbach* NEAR/3 (alpha or alphas)) |
| #8 | (test NEAR/3 retest) |
| #9 | (reliab* NEAR/3 (test or retest)) |
| #10 | {OR #1-#9} |
| #11 | ((coding or code or coded) NEAR/3 (method or content or scheme or definitions or framework or tool or scale or measure or instrument or procedure or process or data or measure or form or system)) |
| #12 | ((coding or code)):ti |
| #13 | ((interview rating or discourse analysis or content analysis)):ti,ab,kw |
| #14 | {OR #11-#13} |
| #15 | ((Provider* or clinician* or doctor* or oncologist* or cardiologist* or surgeon* or generalist* or obstetrician* or gynecologist* or physician assistant* or advanced practice nurse* or nurse* or nurse anthesthsiologist* or nurse practitioner* anesthesiologist* or midwif* or midwives* or pharmacist* or endocrinologist* or allergist* or dermatologist* or gastroenterologist* or hematologist* or immunologist* or nephrologist* or neurologist* or opthalmologist* or otorhinolaryngologist* or physical therapist* or psychiatrist* or pulmonologist* or radiologist* or rheumatologist* or urologist* or healthcare provider* or healthcare professional* or healthcare practitioner* or healthcare personnel or healthcare specialist* or health provider* or health professional* or health practitioner* or health personnel or health specialist* or medical provider* or medical professional* or medical practitioner* or medical personnel or medical specialist* or primary care provider* or primary care professional* or primary care practitioner* or primary care specialist* or primary care physician* or family provider* or family clinician* or family doctor* or family physician* or gynaecologist* or veterinarian or dentist* or general practitioner or general practice doctor or pediatrician or palliative care physician)):ti,ab,kw |
| #16 | ((patient* or client* or inpatient* or outpatient* or person* or spokesperson* or spouse* or advocate* or companion* or parent* or interpreter* or caretaker* or caregiver* or navigator* or patient* spokesperson* or patient* spouse* or patient* advocat* or patient* companion* or patient* parent* or patient* interpreter* or patient* caretaker* or patient* caregiver* or patient* navigator* or family* or family member* or significant other)):ti,ab,kw |
| #17 | #15 or #16 |
| #18 | ((Communicat* or interact* or verbal behaviour* or verbal behavior* or verbal interaction* or non-verbal behaviour* or non-verbal behavior* or non-verbal interaction* or interpersonal behavior* or interpersonal behaviour* or interpersonal skill* or participa* behavior* or body language* or patient provider interaction* or verbal communication* or non-verbal communication* or participation* or patient-centered interviewing* or verbal response* or non-verbal response* or emotional sequence* or information-sharing* or disclosure* or patient* interviewing or relationship building or empathy or decision making or shared decision making or patient involvement or consult*)):ti,ab,kw |
| #19 | #17 and #18 |
| #20 | #10 AND #14 AND #19 |

| CINAHL(EBSCO) | |
| --- | --- |
| #1 | ( TI ( (Development or validation or "construct validation" or "construct validity" or "cultural adaptation" or "cultural validation" or "cultural validity" or "cross-cultural validity" or "cross-cultural validation" or content validity or content validation or "reproducibility of results" or reproducibility or psychometrics or psychometr* or "observer variation" or "observer variation" or "discriminant analysis" or reliability or validity or coefficient or internal consistency or agreement or test retest or stability or interrater or inter-rater or intrarater or intra-rater or intertester or inter-tester or intratester or intra-tester or interobserver or inter-observer or intraobserver or intra-observer or interexaminer or inter-examiner or intraexaminer or intra-examiner or interindividual or inter-individual or intraindividual or intra-individual or kappa or kappas or "coefficient of variation" or repeatab* or generalizability or generalization or generalisability generalisability or concordance or discriminative or "known group" or "factor analysis" or "factor analyses" or dimensionality or subscale or subscales or "item discriminant" or "interscale correlation" or "interscale correlations" or "individual variability" or interpretability or "ceiling effect" or "floor effect" or "cross-cultural equivalence" or test-retest) ) OR AB ( (Development or validation or "construct validation" or "construct validity" or "cultural adaptation" or "cultural validation" or "cultural validity" or "cross-cultural validity" or "cross-cultural validation" or content validity or content validation or "reproducibility of results" or reproducibility or psychometrics or psychometr* or "observer variation" or "observer variation" or "discriminant analysis" or reliability or validity or coefficient or internal consistency or agreement or test retest or stability or interrater or inter-rater or intrarater or intra-rater or intertester or inter-tester or intratester or intra-tester or interobserver or inter-observer or intraobserver or intra-observer or interexaminer or inter-examiner or intraexaminer or intra-examiner or interindividual or inter-individual or intraindividual or intra-individual or kappa or kappas or "coefficient of variation" or repeatab* or generalizability or generalization or generalisability generalisability or concordance or discriminative or "known group" or "factor analysis" or "factor analyses" or dimensionality or subscale or subscales or "item discriminant" or "interscale correlation" or "interscale correlations" or "individual variability" or interpretability or "ceiling effect" or "floor effect" or "cross-cultural equivalence" or test-retest) ) ) OR ( ( ((replicable or replicability or repeated) N2 (measure or measures or findings or result or results or test or tests)) ) OR (intraclass N2 (correlation or correlations)) OR ( ((error or errors) N2 (measure or measures or correlates or correlation or correlations or evaluation evaluate or accuracy or accurate or precision or mean)) ) OR (limit N2 detection) OR ( (small N2 (real or detectable) N2 (change or difference)) ) OR ( (cronbach N2 (alpha or alphas)) ) OR (test N2 retest) OR ( (reliable or reliability N3 (test or retest)) ) ) |
| #2 | TI ( ( (Provider or providers or clinician or clinician or doctor or doctors or oncologist or oncologists or cardiologist or cardiologists or surgeon or surgeons or generalist or generalists or obstetrician or obstetricians or gynecologist or gynecologists or “physician assistant” or “physician assistants” or “advanced practice nurse” or “advanced practice nurses” or nurse or nurses or “nurse anesthesiologist” or “nurse anesthesiologists” or “nurse practitioner” or “nurse practitioners” or anesthesiologist or midwife or midwifery or midwives or pharmacist or pharmacists or endocrinologist or endocrinologists or allergist or allergists or dermatologist or dermatologists or gastroenterologist or gastroenterologists or hematologist or hematologists or immunologist or immunologists or nephrologist or nephrologists or neurologist or neurologists or ophthalmologist or opthalmologists or otorhinolaryngologist or otorhinolaryngologists or “physical therapist” or “physical therapists” or psychiatrist or psychiatrists or pulmonologist or pulmonologists or radiologist or radiologists or rheumatologist or rheumatologists or urologist or urologists or “healthcare provider” or “healthcare providers” or “healthcare professional” or “healthcare professionals” or “healthcare practitioner” or “healthcare practitioners” or “healthcare personnel” or “healthcare specialist” or “healthcare specialists” or “health provider” or “health providers” or “health professional” or “health professionals” or “health practitioner” or “health practitioners” or “health personnel” or “health specialist” or “health specialists” or “medical provider” or “medical providers” or “medical professional” or “medical professionals” or “medical practitioner” or “medical practitioners” or “medical personnel” or “medical specialist” or “medical specialists” or “primary care provider” or “primary care providers” or “primary care professional” or “primary care professionals” or “primary care practitioner” or “primary care practitioners” or “primary care specialist” or “primary care specialists” or “primary care physician” or “primary care physicians” or “family provider” or “family providers” or “family clinician” or “family clinicians” or “family doctor” or “family doctors” or “family physician” or “family physicians” or gynaecologist or gynaecologists or veterinarian or veterinarians or dentist or dentists or “general practitioner” or “general practice doctor” or pediatrician or pediatricians or “palliative care physician”) ) OR ( (patient or patients or client or clients or inpatient or inpatients or outpatient or outpatients or person or persons or spokesperson or spokespersons or spouse or advocate or advocates or companion or companions or parent or parents or interpreter or interpreters or caretakers or caregiver or caregivers or navigator or navigators or patient or patients or spokesperson or spokespersons or patient or patients or “patient companion” or “patient companions” or “patient navigator” or “patient navigators” or family or families or “family member” or “family members” or “significant other”) ) ) OR AB ( ( (Provider or providers or clinician or clinician or doctor or doctors or oncologist or oncologists or cardiologist or cardiologists or surgeon or surgeons or generalist or generalists or obstetrician or obstetricians or gynecologist or gynecologists or “physician assistant” or “physician assistants” or “advanced practice nurse” or “advanced practice nurses” or nurse or nurses or “nurse anesthesiologist” or “nurse anesthesiologists” or “nurse practitioner” or “nurse practitioners” or anesthesiologist or midwife or midwifery or midwives or pharmacist or pharmacists or endocrinologist or endocrinologists or allergist or allergists or dermatologist or dermatologists or gastroenterologist or gastroenterologists or hematologist or hematologists or immunologist or immunologists or nephrologist or nephrologists or neurologist or neurologists or ophthalmologist or opthalmologists or otorhinolaryngologist or otorhinolaryngologists or “physical therapist” or “physical therapists” or psychiatrist or psychiatrists or pulmonologist or pulmonologists or radiologist or radiologists or rheumatologist or rheumatologists or urologist or urologists or “healthcare provider” or “healthcare providers” or “healthcare professional” or “healthcare professionals” or “healthcare practitioner” or “healthcare practitioners” or “healthcare personnel” or “healthcare specialist” or “healthcare specialists” or “health provider” or “health providers” or “health professional” or “health professionals” or “health practitioner” or “health practitioners” or “health personnel” or “health specialist” or “health specialists” or “medical provider” or “medical providers” or “medical professional” or “medical professionals” or “medical practitioner” or “medical practitioners” or “medical personnel” or “medical specialist” or “medical specialists” or “primary care provider” or “primary care providers” or “primary care professional” or “primary care professionals” or “primary care practitioner” or “primary care practitioners” or “primary care specialist” or “primary care specialists” or “primary care physician” or “primary care physicians” or “family provider” or “family providers” or “family clinician” or “family clinicians” or “family doctor” or “family doctors” or “family physician” or “family physicians” or gynaecologist or gynaecologists or veterinarian or veterinarians or dentist or dentists or “general practitioner” or “general practice doctor” or pediatrician or pediatricians or “palliative care physician”) ) OR ( (patient or patients or client or clients or inpatient or inpatients or outpatient or outpatients or person or persons or spokesperson or spokespersons or spouse or advocate or advocates or companion or companions or parent or parents or interpreter or interpreters or caretakers or caregiver or caregivers or navigator or navigators or patient or patients or spokesperson or spokespersons or patient or patients or “patient companion” or “patient companions” or “patient navigator” or “patient navigators” or family or families or “family member” or “family members” or “significant other”) ) ) |
| #3 | TI ( (Communication or communicating or communications or interaction or interactions or “verbal behavior” or “verbal behaviors” or “verbal interaction” or “verbal interactions” or “non-verbal behavior” or “non-verbal behaviors” or “non-verbal interaction” or “non-verbal interaction” or “interpersonal behavior” or “interpersonal behaviors” or “interpersonal behavior” or “interpersonal behaviours” or “interpersonal skill” or “interpersonal skills” or “participant behavior” or “participant behaviors” or “body language” or “patient provider interaction” or “patient provider interactions” or “verbal communication” or “verbal communications” or “non-verbal communication” or “non-verbal communications” or participation or “patient-centered interviewing” or “patient-centered interview” or “patient-centered interviews” or “verbal response” or “verbal responses” or “non-verbal response” or “non-verbal responses” or “emotional sequence” or “emotional sequences” or “information sharing” or disclosure or disclosures or “patient interviewing” or “relationship building” or empathy or “decision making” or “shared decision making” or “patient involvement” or consult or consultation) ) OR AB ( (Communication or communicating or communications or interaction or interactions or “verbal behavior” or “verbal behaviors” or “verbal interaction” or “verbal interactions” or “non-verbal behavior” or “non-verbal behaviors” or “non-verbal interaction” or “non-verbal interaction” or “interpersonal behavior” or “interpersonal behaviors” or “interpersonal behavior” or “interpersonal behaviours” or “interpersonal skill” or “interpersonal skills” or “participant behavior” or “participant behaviors” or “body language” or “patient provider interaction” or “patient provider interactions” or “verbal communication” or “verbal communications” or “non-verbal communication” or “non-verbal communications” or participation or “patient-centered interviewing” or “patient-centered interview” or “patient-centered interviews” or “verbal response” or “verbal responses” or “non-verbal response” or “non-verbal responses” or “emotional sequence” or “emotional sequences” or “information sharing” or disclosure or disclosures or “patient interviewing” or “relationship building” or empathy or “decision making” or “shared decision making” or “patient involvement” or consult or consultation) ) |
| #4 | ( TI ( ((coding or code or coded) N3 (method or content or scheme or definitions or framework or tool or scale or measure or instrument or procedure or process or data or measure or form or system) ) OR AB ( ((coding or code or coded) N3 (method or content or scheme or definitions or framework or tool or scale or measure or instrument or procedure or process or data or measure or form or system) ) ) OR ( TI ( coding or code ) OR ( "interview rating" or "discourse analysis" or "content analysis" ) ) |
| #5 | 1 AND 2 AND 3 AND 4 |

| PsycINFO (EBSCO) | |
| --- | --- |
| 1 | ( TI ( (Development or validation or "construct validation" or "construct validity" or "cultural adaptation" or "cultural validation" or "cultural validity" or "cross-cultural validity" or "cross-cultural validation" or content validity or content validation or "reproducibility of results" or reproducibility or psychometrics or psychometr* or "observer variation" or "observer variation" or "discriminant analysis" or reliability or validity or coefficient or internal consistency or agreement or test retest or stability or interrater or inter-rater or intrarater or intra-rater or intertester or inter-tester or intratester or intra-tester or interobserver or inter-observer or intraobserver or intra-observer or interexaminer or inter-examiner or intraexaminer or intra-examiner or interindividual or inter-individual or intraindividual or intra-individual or kappa or kappas or "coefficient of variation" or repeatab* or generalizability or generalization or generalisability generalisability or concordance or discriminative or "known group" or "factor analysis" or "factor analyses" or dimensionality or subscale or subscales or "item discriminant" or "interscale correlation" or "interscale correlations" or "individual variability" or interpretability or "ceiling effect" or "floor effect" or "cross-cultural equivalence" or test-retest) ) OR AB ( (Development or validation or "construct validation" or "construct validity" or "cultural adaptation" or "cultural validation" or "cultural validity" or "cross-cultural validity" or "cross-cultural validation" or content validity or content validation or "reproducibility of results" or reproducibility or psychometrics or psychometr* or "observer variation" or "observer variation" or "discriminant analysis" or reliability or validity or coefficient or internal consistency or agreement or test retest or stability or interrater or inter-rater or intrarater or intra-rater or intertester or inter-tester or intratester or intra-tester or interobserver or inter-observer or intraobserver or intra-observer or interexaminer or inter-examiner or intraexaminer or intra-examiner or interindividual or inter-individual or intraindividual or intra-individual or kappa or kappas or "coefficient of variation" or repeatab* or generalizability or generalization or generalisability generalisability or concordance or discriminative or "known group" or "factor analysis" or "factor analyses" or dimensionality or subscale or subscales or "item discriminant" or "interscale correlation" or "interscale correlations" or "individual variability" or interpretability or "ceiling effect" or "floor effect" or "cross-cultural equivalence" or test-retest) ) ) OR ( ( ((replicable or replicability or repeated) N2 (measure or measures or findings or result or results or test or tests)) ) OR (intraclass N2 (correlation or correlations)) OR ( ((error or errors) N2 (measure or measures or correlates or correlation or correlations or evaluation evaluate or accuracy or accurate or precision or mean)) ) OR (limit N2 detection) OR ( (small N2 (real or detectable) N2 (change or difference)) ) OR ( (cronbach N2 (alpha or alphas)) ) OR (test N2 retest) OR ( (reliable or reliability N3 (test or retest)) ) ) |
| 2 | TI ( ( (Provider or providers or clinician or clinician or doctor or doctors or oncologist or oncologists or cardiologist or cardiologists or surgeon or surgeons or generalist or generalists or obstetrician or obstetricians or gynecologist or gynecologists or “physician assistant” or “physician assistants” or “advanced practice nurse” or “advanced practice nurses” or nurse or nurses or “nurse anesthesiologist” or “nurse anesthesiologists” or “nurse practitioner” or “nurse practitioners” or anesthesiologist or midwife or midwifery or midwives or pharmacist or pharmacists or endocrinologist or endocrinologists or allergist or allergists or dermatologist or dermatologists or gastroenterologist or gastroenterologists or hematologist or hematologists or immunologist or immunologists or nephrologist or nephrologists or neurologist or neurologists or ophthalmologist or opthalmologists or otorhinolaryngologist or otorhinolaryngologists or “physical therapist” or “physical therapists” or psychiatrist or psychiatrists or pulmonologist or pulmonologists or radiologist or radiologists or rheumatologist or rheumatologists or urologist or urologists or “healthcare provider” or “healthcare providers” or “healthcare professional” or “healthcare professionals” or “healthcare practitioner” or “healthcare practitioners” or “healthcare personnel” or “healthcare specialist” or “healthcare specialists” or “health provider” or “health providers” or “health professional” or “health professionals” or “health practitioner” or “health practitioners” or “health personnel” or “health specialist” or “health specialists” or “medical provider” or “medical providers” or “medical professional” or “medical professionals” or “medical practitioner” or “medical practitioners” or “medical personnel” or “medical specialist” or “medical specialists” or “primary care provider” or “primary care providers” or “primary care professional” or “primary care professionals” or “primary care practitioner” or “primary care practitioners” or “primary care specialist” or “primary care specialists” or “primary care physician” or “primary care physicians” or “family provider” or “family providers” or “family clinician” or “family clinicians” or “family doctor” or “family doctors” or “family physician” or “family physicians” or gynaecologist or gynaecologists or veterinarian or veterinarians or dentist or dentists or “general practitioner” or “general practice doctor” or pediatrician or pediatricians or “palliative care physician”) ) OR ( (patient or patients or client or clients or inpatient or inpatients or outpatient or outpatients or person or persons or spokesperson or spokespersons or spouse or advocate or advocates or companion or companions or parent or parents or interpreter or interpreters or caretakers or caregiver or caregivers or navigator or navigators or patient or patients or spokesperson or spokespersons or patient or patients or “patient companion” or “patient companions” or “patient navigator” or “patient navigators” or family or families or “family member” or “family members” or “significant other”) ) ) OR AB ( ( (Provider or providers or clinician or clinician or doctor or doctors or oncologist or oncologists or cardiologist or cardiologists or surgeon or surgeons or generalist or generalists or obstetrician or obstetricians or gynecologist or gynecologists or “physician assistant” or “physician assistants” or “advanced practice nurse” or “advanced practice nurses” or nurse or nurses or “nurse anesthesiologist” or “nurse anesthesiologists” or “nurse practitioner” or “nurse practitioners” or anesthesiologist or midwife or midwifery or midwives or pharmacist or pharmacists or endocrinologist or endocrinologists or allergist or allergists or dermatologist or dermatologists or gastroenterologist or gastroenterologists or hematologist or hematologists or immunologist or immunologists or nephrologist or nephrologists or neurologist or neurologists or ophthalmologist or opthalmologists or otorhinolaryngologist or otorhinolaryngologists or “physical therapist” or “physical therapists” or psychiatrist or psychiatrists or pulmonologist or pulmonologists or radiologist or radiologists or rheumatologist or rheumatologists or urologist or urologists or “healthcare provider” or “healthcare providers” or “healthcare professional” or “healthcare professionals” or “healthcare practitioner” or “healthcare practitioners” or “healthcare personnel” or “healthcare specialist” or “healthcare specialists” or “health provider” or “health providers” or “health professional” or “health professionals” or “health practitioner” or “health practitioners” or “health personnel” or “health specialist” or “health specialists” or “medical provider” or “medical providers” or “medical professional” or “medical professionals” or “medical practitioner” or “medical practitioners” or “medical personnel” or “medical specialist” or “medical specialists” or “primary care provider” or “primary care providers” or “primary care professional” or “primary care professionals” or “primary care practitioner” or “primary care practitioners” or “primary care specialist” or “primary care specialists” or “primary care physician” or “primary care physicians” or “family provider” or “family providers” or “family clinician” or “family clinicians” or “family doctor” or “family doctors” or “family physician” or “family physicians” or gynaecologist or gynaecologists or veterinarian or veterinarians or dentist or dentists or “general practitioner” or “general practice doctor” or pediatrician or pediatricians or “palliative care physician”) ) OR ( (patient or patients or client or clients or inpatient or inpatients or outpatient or outpatients or person or persons or spokesperson or spokespersons or spouse or advocate or advocates or companion or companions or parent or parents or interpreter or interpreters or caretakers or caregiver or caregivers or navigator or navigators or patient or patients or spokesperson or spokespersons or patient or patients or “patient companion” or “patient companions” or “patient navigator” or “patient navigators” or family or families or “family member” or “family members” or “significant other”) ) ) |
| 3 | TI ( (Communication or communicating or communications or interaction or interactions or “verbal behavior” or “verbal behaviors” or “verbal interaction” or “verbal interactions” or “non-verbal behavior” or “non-verbal behaviors” or “non-verbal interaction” or “non-verbal interaction” or “interpersonal behavior” or “interpersonal behaviors” or “interpersonal behavior” or “interpersonal behaviours” or “interpersonal skill” or “interpersonal skills” or “participant behavior” or “participant behaviors” or “body language” or “patient provider interaction” or “patient provider interactions” or “verbal communication” or “verbal communications” or “non-verbal communication” or “non-verbal communications” or participation or “patient-centered interviewing” or “patient-centered interview” or “patient-centered interviews” or “verbal response” or “verbal responses” or “non-verbal response” or “non-verbal responses” or “emotional sequence” or “emotional sequences” or “information sharing” or disclosure or disclosures or “patient interviewing” or “relationship building” or empathy or “decision making” or “shared decision making” or “patient involvement” or consult or consultation) ) OR AB ( (Communication or communicating or communications or interaction or interactions or “verbal behavior” or “verbal behaviors” or “verbal interaction” or “verbal interactions” or “non-verbal behavior” or “non-verbal behaviors” or “non-verbal interaction” or “non-verbal interaction” or “interpersonal behavior” or “interpersonal behaviors” or “interpersonal behavior” or “interpersonal behaviours” or “interpersonal skill” or “interpersonal skills” or “participant behavior” or “participant behaviors” or “body language” or “patient provider interaction” or “patient provider interactions” or “verbal communication” or “verbal communications” or “non-verbal communication” or “non-verbal communications” or participation or “patient-centered interviewing” or “patient-centered interview” or “patient-centered interviews” or “verbal response” or “verbal responses” or “non-verbal response” or “non-verbal responses” or “emotional sequence” or “emotional sequences” or “information sharing” or disclosure or disclosures or “patient interviewing” or “relationship building” or empathy or “decision making” or “shared decision making” or “patient involvement” or consult or consultation) ) |
| 4 | 2 AND 3 |
| 5 | ( TI ( ((coding or code or coded) N3 (method or content or scheme or definitions or framework or tool or scale or measure or instrument or procedure or process or data or measure or form or system) ) OR AB ( ((coding or code or coded) N3 (method or content or scheme or definitions or framework or tool or scale or measure or instrument or procedure or process or data or measure or form or system) ) ) OR ( TI ( coding or code ) OR ( "interview rating" or "discourse analysis" or "content analysis" ) ) |
| 6 | 1 AND 4 AND 5 |
