# Supplementary material for: Systematic review of observational coding systems to assess patient-healthcare professional interactions
Source: Patient Educ Couns. Author manuscript; Available in PMC 2026 Aug 16. (PMC13477582; doi:10.1016/j.pec.2025.108718)
Supplement: Supplementary file 2 [file NIHMS2172109-supplement-2.docx]

**Appendix B / Supplementary Table 1b. Further details for all included articles (N=124)^1^**

| Instrument name (acronym) | First author | Year | Country | Sample | Behaviors assessed: # items, (sub)scales, behaviors NIET | Construct & its definition | Development process a) how was construct developed;  b) item generation and selection;  c) pilot test |
| --- | --- | --- | --- | --- | --- | --- | --- |
| 5 As for Physical Activity | Carroll | 2011 | USA | 135 patients (various conditions; Mean age = 54 yrs; 73.3% female; 67% white, 32% black, 7.5 other. 1.5% Hispanic); 28 physicians (internal medicine & family medicine, 50% female; Avg. yrs. since residency: 14; 57% white, 32% black, 11% other race/ethnicity) | 5 items: Ask, advise, assess, assist, arrange | Behavior change counseling; defined as: 'To ask about current behavior, advise a change, assess readiness to change, assist with goal-setting, and arrange follow-up' | a) based on existing framework; b) review of published definitions of the 5As and examining prior direct-observation measures of the 5As; c) 10 discussions were pilot-coded, after which codes were reviewed and the coding measure was refined |
| 5a-DOC | Lawson | 2009 | USA | N=186 (development sample) and N=739 (evaluation sample) patients. N=5 (development sample) and N=28 (evaluation sample) physicians (primary care) | 5 items: Ask, advise, assess, assist, arrange | Behavior change counseling; defined according to the 5a's framework: 'Ask, Advise, Assess, Assist, and Arrange' | a) literature using the existing framework 5 A's was reviewed; b) activities associated with the 5 goals were selected from literature, and applied to transcribed encounters by an interdisciplinary team of experts. After modification, operationalized definitions for 5 A's were created and checked with the experts in test data set; c) operationalized definitions were iteratively applied to new transcriptions and modified accordingly |

| Instrument name (acronym) | First author | Year | Country | Sample | Behaviors assessed: # items, (sub)scales, behaviors NIET | Construct & its definition | Development process a) how was construct developed;  b) item generation and selection;  c) pilot test |
| --- | --- | --- | --- | --- | --- | --- | --- |
| Active Patient Participation Coding (APPC) scale | Street | 2005 | USA (presumed) | 9 patients (5 men, ethnically diverse (not specified)); 9 providers (physicians, 4 women) | 5 items, 2 subscales: Patient participation: 3 forms of speech (asking questions, expressions of concern, assertive responses); Providers' patient-centered communication: 2 types (partnership building, supportive talk) | Patient participation (patients) & partnership building & supportive talk (providers). Patient participation defined as: The extent to which patients produce verbal responses that have the potential to significantly influence the content and structure of the interaction as well as the health care provider's beliefs and behaviors. Partnership building: communicative acts that encourage patients to discuss their opinions, express feelings, ask questions, and participate in decision making. Supportive talk: statements of reassurance, support, empathy, and other verbal displays of interpersonal sensitivity | a-b) based on previous research; c) NR |
| Advance Care Planning Communication Assessment Tool (ACP-CAT) | Yuen | 2020 | USA (presumed) | 2 simulated patients (advanced cancer); 29 providers (1st year residents Internal Medicine; Mean age=27 yrs, 66% female; conducting pre- and post training simulated consultations) | 22 items, 6 subscales (5 for ACP-steps, 1 general communication skills) and 2 summative items | Advance care planning communication skills; defined as: 'communication focusing on supporting patients to define their ‘‘personal values, life goals, and preferences regarding future medical care’’ | a) literature review and expert consultation; b) items generated based on literature, existing tools and teaching interventions. Item elimimation through consultation of experts; c) five physician raters pilot-tested the instrument by applying it to 5 video-recorded ACP-encounters, followed by discussion and modification |
| Analysis System for Self-Efficacy Training (ASSET) | Zinken | 2008 | UK | 12 patients (Type 1 diabetes; Mean age=48.17 yrs, 7/12 female); 4 (Facilitators of a diabetes self-management program) | 13 items, 6 scales; Self-efficacy driven verbal techniques (Mastery experience: Self-reflection Facilitating pro-active self; Role modelling: Competent other Group solving Sharing obstacles; Verbal persuasion: Elicitation of knowledge Positive feedback Planning for obstacles; Physiological and affective states: Exploration of affective state Exploration of physiological state Outcome expectancies Non-self-efficacy techniques) and patient's response to those techniques | Self-efficacy enhancement using a collection of techniques; defined as: 'the belief in the personal ability to master adversities, whereby 4 sources of self-efficacy are assumed: mastery experience, role modelling, verbal persuasion, and physiological and affective states. Intervention is defined as a collection of techniques, whereby techniques are defined as a concrete description of the procedures used by those delivering the intervention in sufficient detail to enable exact replication’ | a) specific verbal behavioral techniques identified based on published self-efficacy based interventions for diabetes care; b) verbal techniques collected through systematic literature search were used to develop coding criteria. Relevant interventions were observed to identify further techniques; c) coding tool refined and disagreements discussed until agreement improved (good Kappa scores). The coding tool was amended iteratively |
| Instrument name (acronym) | First author | Year | Country | Sample | Behaviors assessed: # items, (sub)scales, behaviors NIET | Construct & its definition | Development process a) how was construct developed;  b) item generation and selection;  c) pilot test |
| Assessment of Brief Behavioural Change Counselling’ tool (ABC tool) | Fouche | 2020 | South Africa | NR (standardized patients, not further specified); NR (nurse practitioners, family medicine registrars, GPs, family physicians) | 22 items; 5 domains: ask, advise, assess, assist, arrange. Domains 4 and/or 5 completed or not depending on patient's readiness to change | Brief behavior change counseling; defined according to the 5A's theory (ask, advise, assess, assist, arrange) with a 'guiding style' derived from motivational interviewing. The guiding style is characterized as a collaborative, evocative, empathic, respectful and focused approach to the person | a) NR; b) an expert panel judged a preliminary version of the tool, including item redundancy, comprehensiveness, accuracy and comprehensibility. Changes were made, and respondent validation performed; c) NR |
| Behavioral Health Treatment Model for patients with Medically Unexplained Symptoms (BHTM-MUS) | Grayson-Sneed | 2018 | USA | 12 simulated patients (age range 38-58; 11/12 Caucasian; 8/11 female). 161 providers (medical residents; 58% male; 44% Asian, 35% Caucasian, 17% other; Level of training 0-3 yrs) | 33 items, 7 domains: 1) Educating and Informing (4 items); 2) Motivating (2 items); 3) Treatment Statements (5 items); 4) Commitment and Goals (2 items); 5) Negotiates Plan (8 items); 6) Non-Emotion Patient-Centered Skills (4 items); and 7) Patient- Centered Emotional Skills (8 items) | Patient-centered skills and BHTM-MUS skills derived from motivational interviewing; definition NR | a) based on two Randomized Controlled Trials; b-c) NR |
| Behavioral Test of Interpersonal Skills (BTIS) | Olson | 1991 | Canada | Patients NR; 41 providers (25 registered nurses and 16 non-professional nursing personnel) | 3 subscales: Feeling (any reference to feelings); Content (the reason for the speaker's feelings); and 'Don't feel' (any attempt to suppress or discourage expression of speakers feeling). Items assessed in three 5-minute segments per interaction | Active listening; defined as: 'The skill of understanding what your patient is saying and feeling and communicating to your patient in your own words what you think he is saying and feeling' | a-c) NR |
| Behaviour Change Counselling Index (BECCI) | Lane | 2005 | UK (presumed) | 183 patients (divided over 9 different data sets, N(range)= 5-74; details not provided); providers NR | 11 items, 4 domains: agenda setting and permission seeking; the how and why of change in behavior; the consultation as a whole; and talk about targets | Behavior change counseling; defined as: An adaptation of motivational interviewing which might be suitable for brief consultations about behavior change in healthcare settings. Its goal is to help the person to talk through the why and how of change, with the practitioner’s main task being to understand how the person is feeling and what plans they might have for change | a) based on existing models; b) literature review & theory/practice of behavior change counseling; c) items generated and subdivided in domains |

| Instrument name (acronym) | First author | Year | Country | Sample | Behaviors assessed: # items, (sub)scales, behaviors NIET | Construct & its definition | Development process a) how was construct developed;  b) item generation and selection;  c) pilot test |
| --- | --- | --- | --- | --- | --- | --- | --- |
| Brief Decision Support Analysis Tool (DSAT-10) | Stacey | 2008 | Canada | 6 (standardized patients making decision about gall bladder surgery, medication for child with attention deficit hyperactivity disorder, amniocentesis, allergy shots, herniated disc, or cholesterol lowering medications; range age, 37-65 yrs, 66% female); 38 nurses (no further details reported) | 10 items relating to 5 elements: decision-making status, knowledge, values/preferences, others' involvement in decision, next steps | Decision support, defined as preparing clients for decision making by providing tailored information, clarifying values, and enhancing self-help skills in decision-making and implementation | a) based on the Ottowa Decision Support Framework; b) used items from existing DSAT instrument, adapted and removed items, and simplified scoring procedure; c) five coders trained on original DSAT instrument coded encounters between standardized patients and experienced call center nurses, and adaptions were made based on their findings |
| Cancode interaction analysis system (Cancode) | Dent | 2005 | Australia | 1 simulated patient; 10 providers (oncologists; 8/10 male; 7/10 <50 yrs old; all 10+ yrs experience) | 4 domains: 1) Source, 5; 2) content, 9; 3) Function, 12; 4) Emotion (6). Items: Verbal (disclose, advise/recommend/influence, open/closed/leading/multiple question, label/judge/criticize, express feelings/seek reassurance, inform/educate, actively support/empathize/reflect/reassure, partnership build, check patient understanding); Nonverbal (touching, distance, observation, body orientation, asymmetry in arm position; sideways lean; leg position asymmetry, relaxation of hands, relaxation of neck; backward lean, facial activity, vocal activity, speech rate, speech volume) | Cancer-specific consultation interaction; definition NR | a) NR; b) adapted from existing model; c) NR; |
| Caring Behavior Coding Scheme (CBCS) | Martensson | 2021 | Sweden | Patients NR; Providers (Nursing students interacting with standardized patients) | # items NR; Example items: Maintaining belief, knowing , being with , doing for, enabling | Patient and provider caring and non caring behavior; caring defined as: ‘nurturing way of relating to a valued other towards whom one feels a personal sense of commitment and responsibility’ | a) NR; b) research question refined to establish what behaviors to code; coding manual developed with list of operational definitions; c) pilot testing and refinement of coding manual |
| Instrument name (acronym) | First author | Year | Country | Sample | Behaviors assessed: # items, (sub)scales, behaviors NIET | Construct & its definition | Development process a) how was construct developed;  b) item generation and selection;  c) pilot test |
| Chronic Pain Coding System (CPCS) | Henry | 2016 | USA | 45 patients (chronic noncancer pain; Mean age = 52.7; 51% female; 56% white; 20% black; 16% Hispanic; 4% other). 33 providers (primary care clinicians and general internists; Mean age = 44.5; 61% female; 48% white, 27% Asian/Pacific Islander; 21% other) | 7 major patient utterance themes; 22 subscales. 6 major clinician utterance themes; 21 subscales. Patient/companion utterances: e.g. evaluation or description of pain, requests for clinician action, requests for information (i.e., questions), evaluation of non-opioid pain treatments, evaluation of opioids, and responses to clinician recommendations. Clinician utterances: e.g., patient-centered communication, treatment recommendations, evaluation of non-opioid pain treatments, evaluation of opioids, and responses to patient requests for actions | Patient-clinician communication, specifically patient-centered communication, presence of negotiations between patient and clinician; patient assessments of their physical pain and assessments of pain treatment options, discussion of opioid-related side effects and opioid dose changes; definition NR except behaviorally | a) list of communication patterns and constructs developed based on clinical experience, existing research, and previously published coding systems; b) list of patterns and constructs organized into utterance-level categories for coding purposes; c) four authors repeatedly applied the initial coding system to two transcripts, met to discuss results, and modified categories until the categories could be applied reliably |
| Coding System for Staff-Patient Interactions (CSSPI) | Kolko | 1989 | USA | 24 patients (Conduct Disorder (65%), Attention Deficit Disorder (25%), Major Depression (10%); Mean age =9.8 yrs; range, 5-13 yrs). 31 providers (24 psychiatric nurses, 7 clinical assistants; Mean age = 29.8 yrs; 74% female) | 11 items, 3 subscales: Staff antecedent (command, warning, social; 3 items); patient behavior (compliance/noncompliance, inappropriate, physical aggression, social; 4 items); staff consequence (praise, bonus reward, social, redirection, fine, time-out, no response, no opportunity for a response; 8 items). Items assessed in 30 sec intervals within a 5 min. period | Adherence to contingency management procedures; definition NR | a) NR; b) the unit's behavior management procedures manual; c) NR |

| Instrument name (acronym) | First author | Year | Country | Sample | Behaviors assessed: # items, (sub)scales, behaviors NIET | Construct & its definition | Development process a) how was construct developed;  b) item generation and selection;  c) pilot test |
| --- | --- | --- | --- | --- | --- | --- | --- |
| Content Coding for Contextualization of Care (4C) | Weiner | 2014 | USA | Patients NR; Providers (internal medicine residents) | # items NR, 10 contextual domains. Contextual Factors, Contextual Red Flags, Contextual Probes, Contextualized Plans of Care relating to 1 of 10 domains | Physician attention and responsiveness to patient context when planning care; Patient context defined as: 'Everything expressed outside of the boundaries of a patient’s skin that is relevant to their care'. Contextualizing care defined as: 'The process of identifying contextual factors through contextual probes of any observed contextual red flags in order to formulate a contextualized plan of care' | a) research team discussion; b) development within team; c) one coder identified contextual red flags in medical charts, 2 others coded encounters; coders reviewed progress in developing protocol in (bi)weekly meetings and tested multiple coding strategies in mini-experiments involving typically 20-30 encounters (total, 170 encounters) |
| Davis Observation Code (DOC) | Callahan | 1991 | USA | 60 patients; 31 providers (residents in training) | 20 items: Chatting, Structuring Interaction, Counseling, History Taking, Family Information, Treatment Effects, Health Knowledge, Evaluation Feedback, Physical Examination, Patient Question, Compliance, Preventive Services, Health Education, Health Promotion, Planning Treatment, Exercise, Smoking Behavior, Nutrition, Substance Use, Procedure. No subscales. Items assessed in 15-second intervals | Verbal physician behaviors relating to disease prevention, health education, health promotion, compliance checking; definition NR | a) NR; b) categories of behavior developed based on medical education textbooks, observation of videotaped physician-patient interactions, theories of good medical interaction, relevant health-risk behaviors, and behavior modification techniques; Each behavior was clearly and succinctly defined; c) four observers reviewed videotapes using the categories, and coding exercises were used to revise the definitions to achieve consensus |
| Decision Analysis System for Oncology (DAS-O) | Brown | 2011 | Australia, New Zealand, Switzerland, Germany, Austria | 143 patients (N=70 (early-stage breast cancer) and N=73 (women with confirmed early stage breast cancer)). 46 providers (oncologists; 21 from Australia and New Zealand, 25 from Swiss, German and Austria) | 56 items, 5 domains: (i) establishing the physician-patient team (16), (ii) following a consultation pathway (13), (iii) providing information about standard treatments and clinical trials (20), (iv) promoting clarity (7), (v) avoiding coercion (8) | Establishing a shared decision making framework, and providing clear and unbiased information about standard treatments and clinical trials | a) qualitative analysis of audio recordings of 26 treatment decision making general oncology consultations, to identify strategies to facilitate shared treatment decision making and essential clinical and ethical information about treatments and clinical trials; b) strategies were operationalized as a set of discrete items; c) NR |
| Instrument name (acronym) | First author | Year | Country | Sample | Behaviors assessed: # items, (sub)scales, behaviors NIET | Construct & its definition | Development process a) how was construct developed;  b) item generation and selection;  c) pilot test |
| Decision Support Analysis Tool (DSAT) | Butow | 2010 | Australia, New Zealand, Switzerland, Germany, Austria | 55 (early stage breast cancer; Mean age=52.5 yrs, range 31-81 yrs, 100% female); 20 (n=3 surgical, n=6 radiation, n=11 medical oncologists; Mean age=47 yrs, range, 33-62 yrs, 9/20 female) | *see Guimond et al., 2003* | *see Guimond et al., 2003* | a-c) n/a |
| Decision Support Analysis Tool (DSAT) | Guimond | 2003 | Canada | 34 (menopausal patients, 100% female); 34 family physicians | 22 items, 6 categories of decision support skills: 1. Discuss decision making status (5 items), 2. Discuss knowledge/information (5 items), 3. Discuss values (4 items), 4. Discuss support (3 items), 5. Discuss commitment to act (1 item), 6. Discuss learning for future decisions (3 items), Behavior not classified (1 item); 4 categories of communication skills (14 items): 1. Managing the encounter (4 items), 2. Listening (5 items), 3. Questioning (2 items) 4. Sending messages (2 items), Behavior not classified (1 item) | Providers' use of decision support and related communication skills during clinical encounters. Definition NR | a) literature, Ottawa Decision Support framework and Ivey's problem-solving model; b) a panel of researchers, clinicians, and specialists in decision support and communication revised and re-classified items from an existing instrument; c) NR |

| Instrument name (acronym) | First author | Year | Country | Sample | Behaviors assessed: # items, (sub)scales, behaviors NIET | Construct & its definition | Development process a) how was construct developed;  b) item generation and selection;  c) pilot test |
| --- | --- | --- | --- | --- | --- | --- | --- |
| Dementia Competent Social Interaction (DSI) and Culturally Competent Social Interaction (CSI) Coding Schemas | Kim | 2012 | USA | 32 patients (assumed; Korean-American older adults with dementia). 6 providers (direct care staff, Mean age = 31; 4 females; 5 Hispanic) | 27 items (DSI) and 17 items (CSI). For DSI: 12 Competent behaviors (e.g., call resident's name, instruct step-by-step, use simple and familiar words, gentle pace, occasional eye contact); 15 Incompetent behaviors (e.g., no introduction, more than 1 instruction at a time, inappropriate words, monotonous tone of voice/yelling). For CSI: 8 Competent behaviors (e.g., greeting, Korean speech style, face-to-face proximity around arm length); 9 Incompetent behaviors (no/inappropriate call of name, eye-level look down, use of unfamiliar language). Items assessed using direct observation | Dementia competent and culturally competent social interactions; definition NR | a) NR; b) dementia competent social interaction (DSI): literature review of guidelines for dementia care and expert opinions of gerontological nurses, Culturally competent social interaction (CSI): literature review, opinions of Korean experts in gerontological nursing, PI's experience as Korean descendent; c) NR |
| Detail of Essential Elements and Participants in Shared Decision Making (DEEP-SDM) | Clayman | 2012 | USA | 20 patients (9 surgical, Mean age = 49, 11 metastatic breast cancer, Mean age = 51; 15 white, 3 black, 1 Asian, 1 mixed race). Providers (Surgeons (N=NR; early stage) and medical oncologists (N=2; advanced cancer)) | # items NR: Rationale for option, definition of option, process or procedure, risks/cons, benefits/pros, patient self-efficacy, patient preferences and values, patient outcome expectations, patient understanding confirmed, plan for follow-up | Essential elements of shared decision making, defined as: 'SDM is based on the premise that medical or health related decisions should be made with provider and patient input; patients should be involved to the extent that they wish; and their values and preferences are important' | a) based on a preliminary 'Evidence-Based Patient Choice Instrument'; b) items were modified/added based on author's expertise; c) pilot testing on initial sample of early breast cancer recordings (N=9) |
| Elements of Informed Decision Making (IDM) | Braddock | 1999 | USA | 1057 patients (63% some college education, 85% White); 124 physicians (n=65 surgeons, n=59 primary care physicians; 94% male, 92% White) | 7 items assessing 7 elements: 1. Patient's role in decision making, 2. Nature of the decision, 3. Alternatives, 4. Pros and cons, 5. Uncertainties, 6. Assessment of patient understanding, 7. Exploration of patient's preferences. 3 items assessing decision complexity (basic, intermediate, complex) | Informed decision making, defined as: 'a meaningful dialogue between provider and patient, characterized as the completeness of informed decision making as a function of the complexity of the decision; decision defined as verbal commitment to a definitive course of action' | a) literature review and professional consensus; b) earlier work of author and iterative group techniques among providers and laypersons to define completeness for basic, intermediate and complex decisions, and to determine complexity of specific kinds of decisions; c) NR |

| Instrument name (acronym) | First author | Year | Country | Sample | Behaviors assessed: # items, (sub)scales, behaviors NIET | Construct & its definition | Development process a) how was construct developed;  b) item generation and selection;  c) pilot test |
| --- | --- | --- | --- | --- | --- | --- | --- |
| Empathic and Potential Empathic opportunity (E-PE-O) method | Eide | 2004 | Norway | 39 patients (various cancer types; Mean age =49.4 yrs; 59% male). 4 providers (Oncologists; age range 45-55 yrs; 2 women) | # items NR: Empathic opportunity, Potential empathic opportunity. Items assessed within 'thematic episodes' | Empathic opportunities (EO) and potential empathic opportunities (PEO); Empathic opportunity defined as: 'A direct and explicit expression of an emotion by a patient (including a silence or pause that is emotionally loaded).' Potential empathic opportunity defined as: 'A patient statement from which a clinician might infer underlying emotion that has not been explicitly expressed' | a-c) NR |
| Empathic Communication Coding System (ECCS) | Bylund | 2002 | USA | 100 patients. 20 providers (general internists; age range = 30-48 (M=37.7); 20% female; Mean yrs of practice =7.8) | 9 items, 2 domains: Patient's empathic opportunity (progress, challenge, emotion), Physician empathy rating (shared, confirmation, acknowledgment, implicit, perfunctory, denial); 6 levels of physicians' responses; shared, confirmation, acknowledgement, implicit, perfunctory, denial. 5 levels of patients' emotional opportunity intensity | Empathic communication in the physician–patient encounter, specifically patients' empathic opportunities and physicians' responses to these empathic opportunities; Empathic opportunity defined as: 'a clear and direct statement of emotion, progress, or challenge by the patient'. Empathy defined as: 'a physician’s cognitive capacity to understand a patient’s needs, an affective sensitivity to a patient’s feelings, and a behavioral ability to convey empathy to a patient' | a) questionnaires to learn about physician behaviors that patients perceive to be empathic, literature review on communication theory, test to validate the assumed hierarchy in empathic responses; b-c) NR |
| eSEGUE | Assis-Hassid | 2015 | Israel | n/a | 23 items, 5 dimensions: Set the stage (7 items), Elicit information (8 items), Give information (5 items) , Understand the patient’s perspective (2 items), End the encounter (1 item) | Patient-doctor-computer communication; defined as: the physician’s ability to provide patient-centered care while using the computer during the medical encounter | a-b) literature review; c) two-phase experts' panel; phase 1 (N=48) to assess item relevance; phase 2 (N=8) to assess relevance, comprehensiveness. Modifications were made after each round |
| Evidence-Based Patient-Centered Interviewing Method | Grayson-Sneed | 2017 | USA | 12 simulated patients (age range 38-58; 11/12 Caucasian; 8/11 female). 127 providers (medical residents; 61% male; 48% Asian, 27% Caucasian, 5% Black, 1% Hispanic/Latino, 19% other; Level of training 0-3 yrs) | 33 items, 6 subscales: 1) setting the agenda (3 items); 2) physical story (2 items); 3) Personal story (6 items); 4) Emotional story (15 items); 5) Indirect patient-centered skills (4 items); 6) General skills (3 items) | Basic patient-centered skills, i.e. data-gathering and emotion-handling; definition NR, except in behavioral terms | a) literature review; previous coding scheme on patient-centered method; b) items created after construct definition for each item was established; items reviewed for usability by coders; c) NR |
| Instrument name (acronym) | First author | Year | Country | Sample | Behaviors assessed: # items, (sub)scales, behaviors NIET | Construct & its definition | Development process a) how was construct developed;  b) item generation and selection;  c) pilot test |
| Four Habits Coding Scheme (4-HCS) / Physician–patient relationship competence assessment scale | Bellier | 2020 | France | 20 standardized patients (portraying nine personality traits and characters). 200 providers (physician, medical students) | *See Krupat et al., 2006* | *See Krupat et al., 2006* | a-c) n/a |
| Four Habits Coding Scheme (4-HCS) | Catani | 2018 | Brazil | 4 patients. Providers (medical students and residents) | *See Krupat et al., 2006* | *See Krupat et al., 2006* | a-c) n/a |
| Four Habits Coding Scheme (4-HCS) | Jensen | 2010 | Norway | 497 patients (16% bedside, 8% Emergency room, 75% Outpatient; Mean age = 46 yrs (range 0–91); 52% female).71 providers (doctors from various clinical specialties except psychiatry; Mean age = 41 yrs (range: 27–58); 62% female) | *See Krupat et al., 2006* | *See Krupat et al., 2006* | a) NR; b) derived from the core skills referred to in the Four Habit Model; c) NR |
| Four Habits Coding Scheme (4-HCS) | Krupat | 2006 | USA | 100 patients (assumed; Mean age =62 yrs; 50% female; primarily white). 50 providers (residents; senior; 50% female) | 23 items, 4 subscales: Invest in the beginning (6; e.g., demonstrating familiarity with the patient, greeting the patient warmly), Elicit patient’s perspective (3; e.g., eliciting the patient’s understanding of the problem), Demonstrate empathy (4; e.g., helping the patient to identify their emotions), Invest in the end (10; e.g., testing for patient comprehension, determining the acceptability of the treatment plan) | Provider Communication skills, definition NR | a) reported previously (initial development); b) a 4HCS codebook was developed to describe the qualities in communication that correspond to each value on the scale; c) NR |

| Instrument name (acronym) | First author | Year | Country | Sample | Behaviors assessed: # items, (sub)scales, behaviors NIET | Construct & its definition | Development process a) how was construct developed;  b) item generation and selection;  c) pilot test |
| --- | --- | --- | --- | --- | --- | --- | --- |
| Global Behavior Scale (GBS) | Lann-Wolcott | 2011 | USA | 20 patients (long-term care residents with dementia; age range = 69-97 yrs (M = 82.9); 19 white, 1 African American). 52 providers (32 certified nursing assistants, 22 other staff; Mean age = 31.6 yrs; 83% female; 68% white, 30% African American, 6% other; Mean yrs experience =7.5) | 11 items, no subscales. Example behaviors: “treating the resident like a person” vs. “treating the resident in a stereotyped way”; and “treating the resident like he or she is worthy of a relationship” vs. “being indifferent to bond or connection” . Items assessed in four caregiving interactions within full interaction | Person-centered caregiving in gerontology; defined as: 'Caregiving behaviors which are respectful of personhood' | a) literature review; b-c) NR |
| Health Coaching Index (HCI) | Sohl | 2021 | USA (presumed) | Standardized patients (NR). 42 providers (health coaching trainees; n=32 nurses; 98% female) | 4 domains, 18 items: (1) Long-term vision, Designing goals & actions, and Self-monitoring (4 items); (2) use a self-discovery process (4 items); (3) Match information to client interest and needs (1 item); (4) Have a client-centered/mindful approach (9 items) | Health coaching skills; defined as: 'Required behaviors and competence for the practice of empowering the autonomy of patients in facilitating behavior change, consistent with the guidelines of behavior change outlined by Self-Determination Theory' | a) based on literature review and existing theory regarding behavior change; b) 18 initial items were generated from existing measures of relevant constructs and therapies, and some were adapted for consistent wording. Subject matter experts (behavioral research/health coaching) reviewed the original item pool and improved the quality and content validity of items. Cognitive interviews were conducted to refine the items; c) the training phase involved pilot applications of the instrument, leading to updated response options |
| Health Professional-Geriatric Patient Interaction Behavior Rating Code | Adelson | 1982 | USA | Patients (geriatric). 36 providers (Geriatric health professionals (nurse)) | # items NR. Use of patient's name, banter, ask for feedback, give procedural information, compensate for disabilities, social touch, attend to patient comfort, amount of appropriate smiling, pacing of speech, procedure. Items (with the exception of the last three behavioral codes) assessed in three successive 2-min periods of interaction | Relational style of patients and providers; definition NR | a) identified by experts and through review of the literature; b) 15 behaviors combined into 10 operationally defined items by the authors; c) NR |
| Instrument name (acronym) | First author | Year | Country | Sample | Behaviors assessed: # items, (sub)scales, behaviors NIET | Construct & its definition | Development process a) how was construct developed;  b) item generation and selection;  c) pilot test |
| Indicators of Health Behavior Change (IHBC) | Potempa | 2023 | USA | 59 patients (mainly hypertension; 81% female). Providers (N=NR; registered nurse health coaches, >10 yrs experience) | # items NR: Change talk, insight/pattern recognition, self-efficacy and independent agency, sustainability, resilience | Change talk; defined as: 'the language people use in their natural conversations to verbalize the need, desire, or intention to change from the status quo'. Insight/pattern recognition, defined as: 'a cognitive factor that allows individuals to identify meaningful behaviors to meet specific needs'. Self-efficacy/agency; defined as: 'a person’s confidence to execute behaviors necessary for goal attainment (self-efficacy) and the belief that one’s actions will significantly impact health or quality of life (agency)'. Sustainability; defined as: 'a cognitive–behavioral construct that addresses the “why” of the behavior change and the emerging core identity as one that enacts these healthy behaviors'. Resilience; defined as: 'the process of adapting well in the face of adversity, trauma, tragedy, threats, or significant sources of stress—such as family and relationship problems, serious health problems, or workplace and financial stressors' | a) indicators of all constructs were developed by the research team; b) face/content validity was established with independent experts (nursing/behaviorist faculty members); c) NR |
| Informed Decision Making Measure (IDM) | Leader | 2012 | USA | 146 patients (non-acute appointments for prostate cancer screening; 100% male; Mean age = 56 yrs (range 50-69); 70% white). 19 providers (family/community practice; age range 30-59 yrs; 74% male; 90% white) | *See Braddock et al., 1999* | *See Braddock et al., 1999* | a) literature review of models to measure patient involvement in decision making; b) items generated from previously distinguished 9 elements of IDM; c) NR |
| Informed Decision Making Measure (IDM) | Weiss | 2008 | UK | 123 (various clinical issues including chronic disease reviews, acute injuries, infections, illnesses, or post- surgical assessments); 12 general practitioners (Mean age=42 yrs, 33% female) | *See Braddock et al., 1999* | *See Braddock et al., 1999* | a-c) n/a |
| Instrument name (acronym) | First author | Year | Country | Sample | Behaviors assessed: # items, (sub)scales, behaviors NIET | Construct & its definition | Development process a) how was construct developed;  b) item generation and selection;  c) pilot test |
| Kin interaction coding system (KINcode) | Laidsaar-Powell | 2015 | Australia | 72 patients (Cancer, Mean age =60 yrs (range, 28–84); 57% female). 59 adult family members (22 wives/female partner; 20 husbands/male partner). 18 providers (medical/radiation oncologist; 61% male) | 70 items, 4 domains: Family member global role (4): passive, supportive, active, dominant across 4 phases of consultation; Family member's behaviors (38, e.g., Overall communication (16)); Oncologist behaviors towards family member (24, e.g., information exchange (5)); Patient behaviors towards family member (14, e.g., determination of a decision (2)) | Communication and decision-making behaviors; definition NR | a) NR;  b) items based on: empirical studies; oncologist/patient/family behaviors identified via systematic review, qualitative interviews, content analysis of 20 actual consultations; review of dyadic communication coding systems, dyadic decision-making coding systems, and decision-making theory; a list of behaviors was developed via author discussion; draft coding system was cross-checked with other instruments for comprehensiveness;  c) application to 10 consultation resulted in refinements |
| Liverpool-MAAS | Enzer | 2003 | UK | 13 patients (N=4 (pilot sample) and N=71 (assumed; test sample; age range 18 to >75 yrs; 5 female; 8 White)); 12 providers (N=4 (pilot sample; GPs) and N=8 (test sample; GPs; age range 35-60 yrs; 4 female; 5 White, 3 Asian)) | 95 items, 6 subscales: Exploring; history taking; attitudes; presenting solutions; structuring interview; interpersonal skills; communicative skills | Consultation competence; defined as: 'The range of skills a medical practitioner needs in a meeting with a patient, including communication skills to promote information flow and interpersonal skills to establish rapport with the patient' | a) based on existing scale; b) items added to original scale to include patient perspective and UK context; c) NR |
| Manchester Observation Code (MOC) for genetic counseling | Liede | 2000 | NR | 38 clients and their children in N=21 sessions (visiting for genetic counseling). 39 providers (genetic counselors) | 20 items, 4 aspects. For counselor expressions: grammatical form (4; e.g., open question); purpose (6; e.g., giving advice); subject (7; e.g., family history); and cue source (3; e.g., patient-initiated, non-verbal) | Communication behavior specific to genetic counseling interviews; definition NR | a) in the early stages of design, methods described in the literature for teaching psychiatric interviewing skills to general practitioners were modified, enhanced, and tested; b-c) NR |

| Instrument name (acronym) | First author | Year | Country | Sample | Behaviors assessed: # items, (sub)scales, behaviors NIET | Construct & its definition | Development process a) how was construct developed;  b) item generation and selection;  c) pilot test |
| --- | --- | --- | --- | --- | --- | --- | --- |
| Mappin'SDM | Kasper | 2012 | Germany | 40 patients (55% male); 10 providers (physicians: GPs, MS-neurologists, and dentists; 7/10 male) | 15 items, each scored 3 times (for provider, patient, and dyad): defining problem, equipoise statement, preferred communication approach, listing options, pros & cons, expectations, indicating source of recommendations/evidence, doctor's evaluation of patient's understanding, patient's evaluation of physician's understanding, opportunity for questions (from patient), opportunity for questions (from physician), role attribution, supporting strategies of decision-making, indicate decision, follow up arrangements | Shared decision-making behaviors and results (extent of actual involvement achieved). No definitive definition of shared decision-making provided. Behaviors: "behaviors attempting to involve the two parties in the decision-making process." Results: "the perceived (communication-) result in terms of SDM" | a) based on existing constructs and measures (e.g., OPTION)'b) adapted from an existing coding instrument (OPTION-12); c) adaptations made by the authors, not specified |
| Mappin'SDM (Norwegian) | Kienlin | 2017 | Norway | 32 patients (diagnoses NR; Mean age=45 yrs (SD=24), 60% female); 23 physicians (internal medicine, surgical, pediatrics, neurology, gynecology/obstetrics; Mean age=42 yrs (SD=6); 25% female) | *see Kasper et al., 2012* | *see Kasper et al., 2012* | a) NR; b-c) n/a |
| Measure of Patient-Centered Communication (MPCC) | Shields | 2005 | USA | 2 Standardized patients; 50 patients (visiting a primary care physician; 62% female; 84% white, 11% Black, 5% other). 100 providers (primary care physicians; Mean age = 45; 77% male) | # items n/a. Relative total number of emotion words spoken by the physicians | Physicians' use of emotion words; definition NR | a) NR; b) based on all positive/negative emotions listed in the Positive and Negative Affect Scale (PANAS); c) NR |

| Instrument name (acronym) | First author | Year | Country | Sample | Behaviors assessed: # items, (sub)scales, behaviors NIET | Construct & its definition | Development process a) how was construct developed;  b) item generation and selection;  c) pilot test |
| --- | --- | --- | --- | --- | --- | --- | --- |
| Medical Interaction Process System (MIPS) | Ford | 2000 | UK | Patients (cancer). Providers (oncology). | # items NR. 15 content categories (topic addressed): e.g. medical information, psychosocial feelings or symptoms, administrative/practical details). 16 dependent modes of exchange (process/function of utterance): e.g., checking understanding, giving reassurance. 14 independent modes (e.g., agreement, asking for repetition). 7 global affective (paralinguistic) categories (e.g., doctor sensitivity, patient assertiveness), 7 non-verbal (kinesic) categories (e.g., body lean, eye contact) | Information transfer in terms of content and relationship in oncologist-cancer patient encounters | a) literature review; b) the classification system of the MIPS was developed by combining categories adapted from the RIAS with those derived from the detailed examination and analysis of previously collected audio and videotapes of doctor-patient/simulated patient interviews in oncology; c) NR |
| Medical Interaction Process System (MIPS) | Ford | 2004 | UK | 40 cancer patients, 20 providers | *See Ford et al., 2000* | *See Ford et al., 2000* | a-c) n/a |
| MEDICODE | Richard | 2006 | Canada | 462 patients (visiting a GP; Mean age = 52.4 yrs; 65% female). 40 providers (GPs working in 15 clinics.; 57% male; Mean age = 39.4 yrs) | 40 items, 4 main themes: general information, knowledge of the drug, discussion of prescription, effects of the drug | Content of medication discussion; defined as 'information that is discussed about medications' | a) based on previous literature and own clinical experience; b) expert committee consisting of psychologists, physicians and pharmacists discussed and clarified items; c) NR |
| Moffitt Accrual Analysis System (MAAS) | Albrecht | 1999 | USA | 48 patients (eligible for a phase II or phase III clinical trial; 94% white; 72-76% female; Mean age = 57.8 to 59.0 yrs). 12 providers (medical oncologists; 10 male; Mean age = 55 yrs) | # items NR. Global judgements (hierarchical rapport, connection, MD-PT trust); MD responsiveness to PT concerns; MD directed toward signed consent; Conformity to legal consent form; Sharing of floor time; Amount info given; MD information orientation; opinion-based MD information orientation | Clinical trial accrual interactions; defined as 'Degree to which physician’s discourse conforms to the language, structure and format of the formal consent form' | a) developed after review and initial testing of extant coding systems; b) on the basis of four initial videotaped interactions; c) NR |

| Instrument name (acronym) | First author | Year | Country | Sample | Behaviors assessed: # items, (sub)scales, behaviors NIET | Construct & its definition | Development process a) how was construct developed;  b) item generation and selection;  c) pilot test |
| --- | --- | --- | --- | --- | --- | --- | --- |
| Motivational Interviewing Skills Code (MISC) | Moyers | 2003 | USA (presumed) | 50 patients (participants in a smoking cessation intervention study among African Americans). 4 providers (motivational interviewing counselors; African American) | 46 items (1st Pass: 12; 2nd pass: 33; 3rd pass: 1). Global assessment of provider: acceptance, egalitarianism, warmth, genuineness, empathy, overall adherence to spirit of motivational interviewing. Global assessment of patient: affect, cooperation, disclosure, engagement. Global assessment of interaction: collaboration, benefit. 27 therapist behaviors and 4 client behaviors; therapist adherence to MI method; ratio of therapist talk time to client talk time. Items assessed in 20-minute segments of consultation | Motivational interviewing skills; motivational interviewing defined as: 'a therapeutic method designed to increase readiness to change in situations where ambivalence stymies adaptive life choices' | a-b) described elsewhere;  c) NR |
| Motivational Interviewing Skills Code - modified (MISC 2.1) | Lord | 2015 | USA | 19 patients (primary care; Drug and alcohol abuse; college aged). Therapists (NR) | 46 items (1st Pass: 12; 2nd pass: 33; 3rd pass: 1). Global assessment of provider: acceptance, egalitarianism, warmth, genuineness, empathy, overall adherence to spirit of motivational interviewing. Global assessment of patient: affect, cooperation, disclosure, engagement. Global assessment of interaction: collaboration, benefit. 27 therapist behaviors and 4 client behaviors; therapist adherence to MI method; ratio of therapist talk time to client talk time. Items assessed in 20-minute segments of consultation | Therapist-patient interactions; Motivational Interviewing (MI) counselor fidelity; definitions NR | a) NR; b) several modifications (described in detail in the manuscript) were made to traditional MISC coding; c) NR |

| Instrument name (acronym) | First author | Year | Country | Sample | Behaviors assessed: # items, (sub)scales, behaviors NIET | Construct & its definition | Development process a) how was construct developed;  b) item generation and selection;  c) pilot test |
| --- | --- | --- | --- | --- | --- | --- | --- |
| Motivational Interviewing Treatment Integrity (MITI) rating system | McMaster | 2015 | USA | 6 standardized patients (both aged 40–60, both female). 27 standardized providers (Motivational interviewing course students; 92% female) | 12 items; ‘Global’ scores (5), Behavior counts (7): Evocation, collaboration, autonomy, direction, empathy, MI consistent and inconsistent behavior, closed/open questions, simple/complex reflections, giving information. Items assessed in 20-min randomly-selected segment of clinical encounter, excluding first 5 min | Motivational interviewing; defined as: 'Person-centered, directive method of talking about change, which uses specific techniques such as open questions and reflective listening' | a-c) NR |
| Motivational Interviewing Treatment Integrity Code (MITI) rating system (Swedish) | Forsberg | 2007 | Sweden | 60 patients at time 1 (30 imprisoned clients, 30 callers to tobacco quit-line). 15 patients at time 2 (5 imprisoned clients, 5 smoking quit-line and 5 gamblers) | *see McMaster et al., 2015* | *see McMaster et al., 2015* | a-c) NR |
| Motivational Interviewing Treatment Integrity Code (MITI) rating system (Swedish) | Forsberg | 2008 | Sweden | 60 patients at time 1 (30 imprisoned clients, 30 callers to tobacco quit-line) | *see McMaster et al., 2015* | *see McMaster et al., 2015* | a-c) n/a |
| Multi-Dimensional Analysis of Patient Outcome Predictions (MD.POP) | Ménard | 2018 | Belgium | 1 simulated patient (cancer). 40 providers (physicians) | 6 items: Object, Framing, Value, Domain, Probability, Form. Items assessed in speech segments including one patient outcome | Verbal discussion of patient outcomes during medical encounters; patient outcomes defined as: 'any potential future medical, psychological or social outcomes' | a) based on existing literature on natural language processing and physician communication about uncertainty; b) 8 dimensions of patient outcomes were defined and these and subsequent versions were repeatedly subjected to an expert group (clinical psychologists, patient-physician communication researchers) to inform the final version and create a coding manual; c) 3 doctoral students and 2 master’s students tested the various versions of the MD.POP on sample transcripts of medical encounters to test interrater reliability |
| Instrument name (acronym) | First author | Year | Country | Sample | Behaviors assessed: # items, (sub)scales, behaviors NIET | Construct & its definition | Development process a) how was construct developed;  b) item generation and selection;  c) pilot test |
| Nonverbal Accommodation Analysis System (NAAS) | D'Agostino | 2011 | USA | 45 patients (oncology). Providers (various oncology specialties) | 10 items, no subscales: Talk time, speech rate, pauses, interruption frequency, simultaneous speech, smiling, laughing, nodding, eye contact, and gesturing. Items assessed in first 2 min and last 2 min of consultations | Nonverbal communication in medical consultations; definition NR | a) based on the communication accommodation theory (CAT); b) items based on the non-verbal portion of an existing CAT coding system. After initial assessment, modifications to the items and method were made based on literature review and discussion; c) the revised version was pilot tested on a small number of consultations to assess feasibility and content, and results were discussed and examined |
| Nonverbal communication in Doctor–Elderly Patient Transactions (NDEPT) | Gorawara-Bhat | 2007 | USA | 50 patients (undergoing routine exam room visits; Mean age =72.5 yrs (range 65-89); 58% female; 80% White, 20% African-American and Hispanic) | 16 items, 3 subscales: Static attributes (furniture, equipment; 7 items), Dynamic attributes (Interaction distance, Vertical height difference, Physical barrier(s), Angle of interaction; 4 items), Kinesic attributes (Stance, Eye contact, Facial expression, Gesture, Touch; 5 items). Items assessed in opening, middle and closing parts of single physician–patient dyadic encounter | Non-verbal communication; non-verbal communication defined as: 'consisting of two major dimensions: (1) the physical setting of exam rooms enclosing interaction and (2) the body language of the physician (kinesic attributes) unfolding within that exam room' | a) review of initial tapes to determine scope of study and nonverbal variables; b) independent observations by three coders to verify previously identified static, dynamic and kinesic attributes and their components, and inductively identify new salient attributes. Discrepancies in coding were resolved in weekly consensus meetings, and adaptations made to the coding scheme; c) 50 tapes were coded by two coders. Additionally, a senior coder coded 20% of tapes and explored discrepancies regarding dynamic attributes by revisiting videotapes and made clarifications in team meetings |
| Instrument name (acronym) | First author | Year | Country | Sample | Behaviors assessed: # items, (sub)scales, behaviors NIET | Construct & its definition | Development process a) how was construct developed;  b) item generation and selection;  c) pilot test |
| NR ("Strength of connectional silence" taxonomy) | Bartels | 2016 | USA | 124 patients and caregivers if available (stage III or IV cancer; 53% non-aggressive cancer; 90% white; 56% female). 41 providers (solid tumor oncologists; Mean age = 44.7 yrs; 71% male; 44% white, 39% Asian; 17% other or missing) | 10 broad dimensions – 5 lexical (e.g., specific prompt, utterance before silence) and 5 musical (e.g., pitch variability). Items assessed in silence utterances | Silence present during interactions; 'Connectional silence'; defined as: 'particular type of silence in patient-physician communication associated with emotional exchange, it presumes to build relationships and be therapeutic for patients, often contain elements of resonance, presence, and attentiveness.' 'Invitational Silence'; defined as: 'A moment where the doctor invites the patient to express an emotion or the patient invites the doctor to recognize an emotional cue'. 'Neutral Silence'; defined as: 'A pause in conversation that does not affect the emotion of the conversation. 'Disengaged Silence'; defined as: 'A silence that seems to embody distraction, specifically when the doctor does not seem engaged in communication with the patient' | a) based on literature;  b) using a modified grounded approach sensitized by literature and in consultation with a linguist; c) conversation segments with one silence each were reviewed by 3 expert physicians who were not study participants and not involved in the coding process (Cohen’s kappa = 0.72) |
| NR (ambivalence and reluctance in palliative care ) | Gupta | 2023 | USA | 70 patients and caregivers (all serious illness; 52% cancer patients; 53% female). 22 providers (palliative clinicians) | 3 items, no subscales: Internal ambivalence, ambivalence between patient and caregiver, patients/caregivers' unwillingness towards discussing a treatment option or actively stating against deciding for themselves | Ambivalence' defined as: 'a difference between having opposing desires and being equivocal about a choice'. 'Reluctance' defined as: 'stating a strong preference against what a clinician recommends or showing hesitancy/unwillingness toward making or sometimes even talking about a care decision' | a) NR; b) 5 coders used inductive coding methods to create a codebook, and diverse patient representatives checked the codes for face validity; c) NR |
| NR (Automatic annotation of information-giving in clinical conversations) | Mayfield | 2014 | USA | 415 peope (living with HIV; ≥19 yrs old); 45 providers (physicians, nurse practitioners or physician assistants providing primary care to HIV-infected patients) | 3 items: give information, request information, other | Information-giving; defined as: 'when a speaker gives information or requests information at the speech act level' | a) literature;  b-c) NR |

| Instrument name (acronym) | First author | Year | Country | Sample | Behaviors assessed: # items, (sub)scales, behaviors NIET | Construct & its definition | Development process a) how was construct developed;  b) item generation and selection;  c) pilot test |
| --- | --- | --- | --- | --- | --- | --- | --- |
| NR (behavior change therapy delivery and receipt) | Hoekstra | 2022 | Canada | 14 patients (chronic spinal cord injuries; Mean age =46 yrs, 5/14 female). 1 provider (personal trainer (counselor); trained in BCT) | 94 items (48 for counselors, 46 for patients), 13 categories: Goals and planning, support strategies, natural consequences, comparison of behavior, monitoring and feedback, associations, repetition and substitution, comparison of outcomes, regulation, antecedents, identity, self-belief, general aspects of the communication and interaction | Behavior change technique delivery and receipt; defined as: 'Counsellor’s delivery of behavior change technique as well as teaching or prompting clients to use behavior change techniques; clients' understandings of these techniques as well as how and when to use them' | a) based on previous literature and coding instruments; b) informed by previous instruments (Michie et al., 2013; Gainforth et al., 2016 and 2019; Ma et al., 2019). Based on previous data, expected codes were drafted; c) two team members independently coded five transcripts to refine and add codes, labels, and definitions based on the coded transcripts. Throughout the pilot coding, there were consensus meetings and discussions with the broader team. Subsequently, two team members coded transcripts from two other sessions using the revised coding method |
| NR (caregiver activation in advanced cancer care) | Dingley | 2017 | USA | 60 patients (various cancers; Mean age =70.2 yrs; 52% male; 98% Caucasian). Patients' caregivers (mostly spouses or adult children; Mean age =59.5 yrs; 70% female; 92% Caucasian) | 9 items, 2 subscales: *Content*: knowledge, skills, and behavioral repertoire to provide care in the home setting. *Processes*: asking questions, being assertive, expressing concerns | Caregiver activation during home hospice care; defined as: 'Communications that reflect the primary elements of activation, [namely] knowledge, skills, and behavioral repertoire to provide care in the home setting' | a) based on existing definition, existing coding scheme; b) through iterative process of individual and collaborative coding and consensus-building; c) coding scheme was pilot-tested and modified in 20 audio recordings by 3 coders |

| Instrument name (acronym) | First author | Year | Country | Sample | Behaviors assessed: # items, (sub)scales, behaviors NIET | Construct & its definition | Development process a) how was construct developed;  b) item generation and selection;  c) pilot test |
| --- | --- | --- | --- | --- | --- | --- | --- |
| NR (client responses to practitioner-delivered behaviour change techniques) | Gainforth | 2016 | UK | 18 patients (Smokers at different stages of quitting). Providers (providing routine behavioral support consultations) | 58 items, 5 subscales: 'Client' BCTs (behavior addressing motivation (13 BCTs), behavior maximizing self-regulatory capacity or skills (15 BCTs), adjuvant activities (4 BCTs), general aspects of the interaction (21 BCTs)) and statements unrelated to smoking or smoking cessation (5 codes) | Client verbal statements in response to behavioral change techniques; 'Client statement' defined as: 'A distinct unit (or chunk) of transcribed client verbal behavior representing a single conversational ‘turn’ following (or preceding) practitioner verbal behavior' | a) existing coding framework for specifying practitioner-delivered Behavioral Change Techniques (BCTs) was adapted; b) 2 researchers inductively developed coding categories using 2 transcripts and checked match with the 'practitioner' framework; c) 2 researchers coded the same 2 transcripts deductively using the 'practitioner' framework and developed corresponding client labels and definitions and coded a third transcript using the coding framework; discrepancies were resolved and used to adapt the framework |
| NR (coding frame for shared decision-making) | Singh | 2010 | Australia | 63 patients (solid tumor considering adjuvant therapy; Mean age =54.9 yrs; 64% female; 57% Australia-born). Providers (medical oncologists) | 20 items, 6 subscales: 1. Establishing the problem (3) 2. Doctor-patient relationship (2) 3. Research evidence (4) 4. Patient perspective (4); 5. Decision making (6); 6. Time issues (1) | Shared decision-making behaviors; defined as: 'A model in which the patient is given information regarding their disease and possible treatments and is a participant along with the physician in medical decision making' | a) based on a literature review of SDM in oncology; b) 23 behaviors were listed based on previous literature and coding systems and reviewed by a multidisciplinary team (medical oncologists, oncology nurses, health psychologist), and then modified, resulting in 20 items; c) coding scheme was applied to 5 consultations, resulting in some revisions |
| NR (coding of patient-provider self-care communication and EMR usage) | Arar | 2006 | USA | 50 patients (various chronic illnesses; 98% male; Mean age = 64.5 yrs (range: 40–86); 44% Hispanic, 40% non-Hispanic white; 12% African American; 2% other). 6 providers (primary care physicians (internists); all non-Hispanic white; 4/6 female; all 8 yrs of experience) | # items NR. Self-care management: medication, physical distress, disease symptoms, self-monitoring activities, exercise and diet, smoking, family support/community resources, emotional distress, alcohol consumption, EMR usage | Self-care management practices; definition NR. EMR usage; defined as: 'any contact with the computer, regardless of whether or not the physician was talking to the patient' | a) content of self-care was defined by the authors. Identification and classification of utterances about self-care by either provider or patient were coded from transcripts; b) a detailed code book was developed to define and specify categories based on self-care literature and methods for coding each self-care element in the transcribed encounters; c) NR |
| Instrument name (acronym) | First author | Year | Country | Sample | Behaviors assessed: # items, (sub)scales, behaviors NIET | Construct & its definition | Development process a) how was construct developed;  b) item generation and selection;  c) pilot test |
| NR (coding scheme to identify the content of general practitioner communication) | Di Caccavo | 2000 | UK | 75 patients (various psychological symptoms; 67% female; 39% aged 16-40 yrs, 40% aged 41-65 yrs, 21% aged >65 yrs). Providers (general practitioners) | # items NR, 16 subscales: courtesy, return visit, positivity, physical symptom description, mental symptom description, normal function symptom description, symptom history, symptom severity, external factors, diagnosis, treatment options, current regime, treatment history, treatment arrangements, mental coping, social support | Content of general practitioner verbal communication behaviors; definition NR | a) open coding of 10 general practice consultations to identify categories of discrete ideas or communicative events; b) categories were organized into higher-order (general description) vs. lower-order specific subcodes) codes; c) application of preliminary version of coding scheme to 12 consultations |
| NR (cognitive strategies used by general practitioners in diagnostic reasoning) | Donner-Banzhoff | 2017 | Germany | 134 patients (Mean age =55 yrs; 63% female). 12 providers (GPs; Median age=53 yrs; 5/12 female) | # items NR. Items assessed during diagnostic episodes | Cognitive strategies (Inductive foraging, Descriptive questioning, Triggered routines, Hypothesis testing). 'Inductive foraging' defined as: 'The is guiding the physician to relevant problem areas'. 'Descriptive questioning' defined as: 'Physician obtains descriptions of symptoms or problems. 'Triggered routines' defined as: 'Physician obtains information on problem area becoming relevant because of symptom or problem mentioned by patient'. 'Hypothesis testing' defined as: 'Physician evaluates defined hypotheses (diseases) potentially explaining the patient’s problem' | a) based on literature; b) extensive discussion and several iterative loops of coding and modification of the coding tree; c) 3 randomly-selected consultations and 3 interviews |
| NR (Complex oral information transfer) | Nordfalk | 2019 | Norway | 34 standardized patients (Multiple Sclerosis, Median age 48 yrs, 74% female); 17 providers (neurologists, mean age 41 yrs, 59% male, 2-29y work experience) | 2 items/dimensions: Count-COPIN (units of provided information), Count-PROPIN (units of information recalled by patient) | Complex information transfer; defined as multiple, uncertain, situation dependent clinical information | a) NR; b) literature search, group discussion; c) concept instrument used by coders on 5 transcripts |
| NR (decision-making around limiting life supporting treatment in neonatal intensive care) | Shaw | 2023 | UK | Parents of 21 critically ill babies (n=27 conversations; n=9 with 1 parent). 14 providers (11 neonatologists, 3 cardiologists; 9 male) | 22 items, 3 subscales: first decision point (8); subsequent decision points (2); parents' responses (12 items in total (2 codes, 1 summative code indicating total parental response)). Items assessed during decision-making sequences | Critical care decision making, specifically (1) the first decision point (e.g. who initiated, how initiated); (2) aspects of any subsequent decision point(s); and (3) how parents responded; definition NR | a) based on previous conversation-analytic findings; b) based on and substantiated by additional conversation analysis; c) NR |
| Instrument name (acronym) | First author | Year | Country | Sample | Behaviors assessed: # items, (sub)scales, behaviors NIET | Construct & its definition | Development process a) how was construct developed;  b) item generation and selection;  c) pilot test |
| NR (Global Consultation Rating Scale (GCRS) adapted for pediatric subspecialties) | Coburn | 2020 | USA | 99 patients (Chronic kidney disease, Mean age = 14.9; 55% male, 52% AA, 40% Caucasian, 9% Other). 18 providers (78% MD, 17% Post-doc, 6% Nurse Practitioner; 78% Female; 62% Caucasian, 33% Asian, 6% African American) | 12 domains (initiating the session, gathering information/problem identification, problem exploration, patient’s perspective, nonverbal communication, developing rapport, providing structure, correct amount and type of information, aiding accurate recall and understanding, incorporating the patient’s perspective, planning and shared decision making, closure) | Adequate provider communication according to Cambridge-Calgary guidelines; defined as: 'the providers combines pertinent information with positive relational components, and is demonstrate by using shared decision-making, active listening, and open-ended questions' | a) based on existing global consultation rating scales; b) adaptations based on authors expertise and literature; c) NR |
| NR (goal communication in palliative care decision-making consultations) | Gramling | 2015 | USA | 72 patients and their families (hospitalized and referred for goals of care clarification or end-of-life decision making; 49% cancer; 32% aged <60 yrs and 31% aged >80yrs; 54% female; 93% white, 7% black) | 11 items, 3 subscales: Patient goal expression (5, e.g., Symptom control); Provider goal expression (2, e.g., Suggestion/example of a potential goal); Other aspects of conversation (4, e.g., Palliative Care team communication about prognosis) | Explicit verbal goal expression in palliative care consultations; 'Goals' defined as: 'What patients want to achieve during their remaining lifetime to guide contemplation of treatment options' | a-b) theoretical and empirical understanding of goals communication in advanced illness; c) NR |
| NR (metaphor use) | Macagno | 2019 | Italy | 13 patients (Diabetes; 7/13 female). 6 providers (diabetes health care providers; 3/6 female; 3/6 physician, 2/6 nurse, 1/6 dietician) | # items NR. Declaration of lack of understanding, alternative interpretation of semantic representation, alternate interpretation of intended purpose of utterance, clarification request, check understanding, irrelevance, no uptake, anticipation | Use and problematic understanding of metaphors; 'Metaphors' defined as: 'Tools for enhancing health care providers' ability to communicate effectively, emphatically and clearly,and as strategies for patients to make sense and explain their illness'; 'Problematic understanding' defined as: 'a potential mismatch between speaker's intended meaning and the hearer's understanding thereof, or (negative definition) as a case in which the hearer's understanding cannot be presumed' | a) coding scheme based on classification of linguistic evidence of problematic understanding, based on the literature. Evidence was classified in 3 categories: strong (direct meta-dialogical evidence), A (indirect meta-dialogical evidence), W (indirect dialogical evidence); b-c) NR |
| Instrument name (acronym) | First author | Year | Country | Sample | Behaviors assessed: # items, (sub)scales, behaviors NIET | Construct & its definition | Development process a) how was construct developed;  b) item generation and selection;  c) pilot test |
| NR (number and type of problems discussed in primary care consultations) | Procter | 2014 | UK | 13 patients (GP visitors) for tool development; 60 patients (GP visitors) to assess reliability; 169 patients for the final study. 2 providers (GPs) for tool development; 30 providers (GPs) to assess reliability; 30 providers for the final study | 27 items, 2 subscales: Issues (10, e.g., medication related), problems/diseases (17) | Complex consultations in general practice; defined as: 'Multiple issues requiring physician action in the form of a decision, diagnosis, treatment, or monitoring' | a) based on Flocke et al.'s method and existing framework; b) expert panel discussion; c) initial coding of 60 recordings |
| NR (Nurse Patient Touch) | Bottorff | 1994 | NR | 8 patients (cancer; 3/8 female). 32 providers (nurses caring for cancer patients) | 13 items, 2 subscales: Interactional context (6, e.g., proximity nurse-patient, nurse activity), Touch event (7, e.g., initiator, location of touch) | Nurse-patient touch, definition NR | a) ethological analysis, assessment of existing observational schedules; b-c) NR |
| NR (Patient active involvement in mental health interaction) | Bonfils | 2017 | USA | 166 patients (treated for severe mental illness; mean age = 44.2y, 57% male, 55% black); 4 providers (2 psychiatrists, 2 nurse practitioners in mental health) | 8 items | Patient involvement; defined as: the skills, knowledge, and confidence needed for patients to participate in managing a chronic illness | a) based on existing theory and more basic instruments; b-c) iterative development by the team, using pilot coding and refinement |
| NR (physicians' compliance-gaining strategies) | Smith | 2004 | USA | 37 patients (visiting a primary care physician; aged 40-60y; 59% female). 18 providers (primary health care providers; 15 male) | 57 items, 10 categories of behavior, 2 dimensions: Directness (Physician states desired action); and Completeness (extent to which a strategy includes a justification). Behavioral categories: Reinforcement, punishment, helping morality, power strategies, understanding, office visit, patient motivation, prior treatment, simple observations, prescriptions, other | Language used by physicians to influence patents; defined as: 'An utterance used when a physician tries to “get the patient to do something”' | a) based on literature regarding compliance gaining strategies; b) examination of published literature, transcripts of medical interviews, and methods used by other doctor–patient communication researchers; c) NR |

| Instrument name (acronym) | First author | Year | Country | Sample | Behaviors assessed: # items, (sub)scales, behaviors NIET | Construct & its definition | Development process a) how was construct developed;  b) item generation and selection;  c) pilot test |
| --- | --- | --- | --- | --- | --- | --- | --- |
| NR (Planning talk within motivational interviewing) | Copeland | 2017 | UK | 50 clients (participating in MI weight loss maintenance program; mean age 50 yrs, 86% female, 90% white British); Providers NR | 2 categories (goals, plans); Goals further specified using 2 criteria (specificity, commitment); For plans, 3 categories (past, continuing/hypothetical, future) and 2 criteria (specificity, commitment) | Discussion of plans and goals; Plan defined as: an action for the future that will help the person achieve a goal and there must be evidence that it was volitional. Goal defined as: future orientated and is a desired state that the person wants to achieve | a) systematic literature search of definitions of 'plans and goals' & thematic analysis within the research group; b) draft coding of MI sessions using definitions identified in the literature, selection of best fitting goals and plans; c) test and continuous refinement of the draft coding system on MI data (20 sessions by one author, 5 sessions by all authors); assessment by a group of 11 researchers outside the development group, who received brief training and provided oral feedback. This resulted in final refinement and clarification |
| NR (safety-netting communication behaviors in primary care encounters) | Edwards | 2019 | UK | 32 patients (Mean age =48 yrs (range 20-83); 53% female; 84% white). 19 providers (GPs; Mean age =47 yrs (range 32-62); 10/19 female; all white) | # items NR, 4 subscales: administrative codes; safety netting contextual codes (4 codes); safety-netting advice codes (14 codes); an additional optional set of problem contextual codes | Safety-netting communication behaviors; defined as: 'Information shared with a patient or their carer designed to help them identify the need to seek further medical help if their condition fails to improve, changes, or if they have concerns about their health (Adapted from Roland et al, 2014)' | a) review of published definitions and adaptation of existing one; b) literature review, search of clinical guidelines and seminal work, consultation of existing codes of related tool, discussion with research team and patient group; c) 2 coders independently coded 93 randomly sampled consultations to test the codebook, and generate and modify codes in iterative process of coding and discussion. Input was then sought on further refinement from 5 members of the public to represent the patient perspective |
| Instrument name (acronym) | First author | Year | Country | Sample | Behaviors assessed: # items, (sub)scales, behaviors NIET | Construct & its definition | Development process a) how was construct developed;  b) item generation and selection;  c) pilot test |
| NR (the process and content of general practice consultations involving the prescription of antibiotic agents) | Cockburn | 1987 | Australia | 177 patients (in primary care receiving antibiotics). 56 providers (primary care) | 12 items, 3 subscales: Informational aspects (4; doctor's information gathering, doctor’s information transfer, patient’s information transfer, patient’s information gathering, social exchange, content of information), Mutuality (2; doctor's efforts at collaboration, patient's suggestions and ideas), Strategies to improve knowledge and compliance (4) | Successful consultation; defined as: 'Patient-centered and involving discussion and agreement between doctor and patient' | a-c) NR |
| NR (transparency in patient-provider communication) | Robins | 2011 | USA | 259 patients (55% female; Mean age = 46.6 yrs (range 18-86); 77% White, 6% Mixed, 3% Asian, 2% Hispanic, ≤ 2% other). 33 providers (primary care physicians; 52% female; Mean age = 43.9 yrs (range 36-59); 76% White, 15% Asian, 3% Mixed, 3% Hispanic) | 9 items, 2 subscales: Process (agenda, framing, meta comment about physical action, physical exam, orchestration); Medical content (explicating diagnostic reasoning and proposed treatment plans in lay terms, providing rationales for treatment/management, demystifying medical language and concepts, sharing the physician’s perspective about how he or she personally would interpret/respond to information) | Transparency in patient-provider communication; defined as: 'The ways in which physicians are explicit about their actions and opinions on matters concerning the health of the patient, the encounter, procedures, and the relationship with the patient' | a) literature review, weekly meetings among group of researchers with expertise in relevant disciplines; b) 4 researchers reviewed 105 interviews independently, followed by review and consensus of identified categories and definitions; c) the final coding scheme and sample of 45 transparency utterances was given to a 5th researcher to coding, Cohen's kappa was calculated |
| NR (verbal interpersonal clinical problem-solving skills) | Pridham | 1980 | USA | 5 patients (3 children, 1 prenatal, 1 adult patient with acute illness). 5 providers (3 family medical residents, 2 nurse clinicians) | 48 items, 16 subscales. Phases: scanning, formulating; appraising, developing willingness/readiness to problem solve, planning, implementing, evaluating. Items assessed during 10-second intervals | Interactive clinical problem-solving; defined as: 'Interactions that delineate issues of concern, formulate goals, specify problems and identify desirable actions that the client is willing to take' | a) developed based on model of interpersonal probem solving by Boyd et al; b) iterative process of empirical tests based on observational data in different medical settings; c) NR |
| NR (workflow task coding scheme) | Asan | 2015 | USA | 18 patients (visiting primary care clinics; age range 25-65 yrs). 6 providers (primary care physician; 4/6 male; Mean age = 45.5 yrs) | # items NR | Interaction styles with EHRs; defined as: 'The amount of time the physician spent typing and gazing at the computer in the visits. Workflow tasks; defined as : 'a validated list of workflow tasks performed by primary care physicians during patient clinic visits' | a) tasks list was a validated list of workflow tasks performed by primary care physicians by Wetterneck et al., 2011; b) based on the validated list, two coders coded each video capturing a primary care visit according to the tasks that occurred. Start and stop times for each task were obtained this way; c) NR |
| Instrument name (acronym) | First author | Year | Country | Sample | Behaviors assessed: # items, (sub)scales, behaviors NIET | Construct & its definition | Development process a) how was construct developed;  b) item generation and selection;  c) pilot test |
| Observing patient involvement (OPTION-12) | Butow | 2010 | Australia, New Zealand, Switzerland, Germany, Austria | N=55 (early stage breast cancer; Mean age=52.5 yrs, range 31-81 yrs, 100% female); N=20 (n=3 surgical, n=6 radiation, n=11 medical oncologists; Mean age=47 yrs, range, 33-62 yrs, 9/20 female) | *see Elwyn et al., 2005* | *see Elwyn et al., 2005* | a-c) n/a |
| Observing patient involvement (OPTION-12) | Elwyn | 2003 | UK | N=186 patients (various conditions, majority of encounters regarded respiratory, musculoskeletal, dermatological, or psychological problems; Mean age=43.3, SD=20.6, range, 4 months-83 yrs, 68% female), N=21 general practitioners (Mean age=38 yrs, 38% female) | *see Elwyn et al., 2005* | *see Elwyn et al., 2005* | a) literature on existing measures of related concepts and competences relevant to patient involvement in decision making, and assessment of clinical practice; b) item formulation involving patients and clinicians; c) pilot-testing of 18- and 16-item versions on simulated consultations, leading to item refinement and scale development in 3 iterative cycles |
| Observing patient involvement (OPTION-12) | Weiss | 2008 | UK | N=123 (various clinical issues including chronic disease reviews, acute injuries, infections, illnesses, or post- surgical assessments); N=12 general practitioners (Mean age=42 yrs, 33% female) | *see Elwyn et al., 2005* | *see Elwyn et al., 2005* | a-c) n/a |

| Instrument name (acronym) | First author | Year | Country | Sample | Behaviors assessed: # items, (sub)scales, behaviors NIET | Construct & its definition | Development process a) how was construct developed;  b) item generation and selection;  c) pilot test |
| --- | --- | --- | --- | --- | --- | --- | --- |
| Observing patient involvement (OPTION-12) (Italian) | Goss | 2007 | Italy | N=235 patients (various, e.g., musculoskeletal, respiratory, cardiovascular, gastrointestinal, psychological, dermatological, genitourinary problems, metabolic problems; Mean age=45 yrs, range, 18–70 yrs, 69% female); N=6 general practitioners (Mean age=46 yrs, SD=6, 0% female) | *see Elwyn et al., 2005* | *see Elwyn et al., 2005* | a-c) n/a |
| Observing patient involvement in decision making (OPTION-5) | Barr | 2015 | USA | 311 patients (65% chest pain, 35% osteoporosis; range mean age, 54.5-70.2 yrs, range age, 27-87 yrs; 73% female); clinicians NR | *see Elwyn et al., 2013* | *see Elwyn et al., 2013* | a-c) n/a |
| Observing patient involvement in decision making (OPTION-5) | Elwyn | 2013 | NA; see development process | n/a; see development process | 5 items: justify the work, forming a team, describing the options, preference elicitation, preference integration | Essential requirements of SDM when providers make an effort to involve patients in decisions, composed of: justifying deliberative work, describing options, information exchange, preference elicitation, and preference integration | a) literature review; b-c) n/a |
| Observing patient involvement in decision making (OPTION-5) | Stubenrouch | 2016 | Netherlands | 60 patients (oncology (n=45) and vascular surgery (n=15), 47-77 yrs, 39 female); 37 providers (oncology (n=38) and vascular surgery (n=15); 38-66 yrs, 15 male) | *see Elwyn et al., 2013* | *see Elwyn et al., 2013* | a-c) n/a |
| Observing patient involvement in decision making (OPTION-5) | Vortel | 2016 | Canada | 27 patients (women with pregnancies at increased risk for genetic conditions; age in yrs, 20-24: 4%, 25-29: 19%, 30-34: 30%, 35-3: 33%, 40-45: 11%, >45: 4%, 100% female); 8 genetic counsellors | *see Elwyn et al., 2013* | *see Elwyn et al., 2013* | a-c) n/a |
| Instrument name (acronym) | First author | Year | Country | Sample | Behaviors assessed: # items, (sub)scales, behaviors NIET | Construct & its definition | Development process a) how was construct developed;  b) item generation and selection;  c) pilot test |
| Observing patient involvement in decision making (OPTION-5) (Chinese) | Chen | 2020 | China | 209 patients (various chronic diseases, mean age=64.4 yrs, 56% female); 10 GPs (mean age=42.4 yrs, 100% female) | *see Elwyn et al., 2013* | *see Elwyn et al., 2013* | a-c) n/a |
| Observing patient involvement in decision making (OPTION-5) (German) | Kölker | 2018 | Germany | 79 patients (primary care, mean age=54.7 yrs, n=51 female); 24 clinicians (n=11 GPs, n=8 internists, n=3 orthopedics, n=2 psychiatrist; mean age=49.4 yrs; 11 female) | *see Elwyn et al., 2013* | *see Elwyn et al., 2013* | a-c) n/a |
| Observing patient involvement in decision making (OPTION-12 revised) | Elwyn | 2005 | UK | N=186 patients (various conditions, majority of encounters regarded respiratory, musculoskeletal, dermatological, or psychological problems; Mean age=43.3, SD=20.6, range, 4 months-83 yrs, 68% female); N=21 general practitioners (Mean age=38 yrs, 38% female) | 12 items: identify the decision, introduce equipoise, assess preferred decision making approach, list options, explain pros and cons, explore patient expectations, explore patient concerns, check patient understanding, offer opportunities for questions, elicit preferred level of involvement, indicate need for decision-making stage, indicate need to review decision | Extent to which clinicians involve patients in decision-making processes; definition NR | a) n/a b) Labels of response categories of existing coding scheme (Elwyn et al., 2003) were revised based on user feedback, with a shift from an attitudinal to a magnitude-based scale c) n/a |
| Observing patient involvement in decision making (OPTION-12 revised) | Kasper | 2011 | Germany | 76 patients (multiple sclerosis decising on immunotherapy; subsample of larger sample described elsewhere); 4 physicians (not further specified) | *see Elwyn et al., 2005* | *see Elwyn et al., 2005* | a-c) n/a |

| Instrument name (acronym) | First author | Year | Country | Sample | Behaviors assessed: # items, (sub)scales, behaviors NIET | Construct & its definition | Development process a) how was construct developed;  b) item generation and selection;  c) pilot test |
| --- | --- | --- | --- | --- | --- | --- | --- |
| Observing patient involvement in decision making (OPTION-12 revised) | Stubenrouch | 2016 | Netherlands | 60 patients (in oncology (n=45) and vascular surgery (n=15), 47-77 yrs, 39 female); 37 providers (in oncology (n=38) and vascular surgery (n=15); 38-66 yrs, 15 male) | *see Elwyn et al., 2005* | *see Elwyn et al., 2005* | a-c) n/a |
| Observing patient involvement in decision making (OPTION-12 revised (German) | Hirsch | 2012 | Germany | 40 patients (indication for discussion of cardiovascular risk and preventive measures; 65% female); 15 general practitioners (range age, 44-56 yrs, 33% female) | *see Elwyn et al., 2005* | *see Elwyn et al., 2005* | a-c) n/a |
| Observing patient involvement in decision making (OPTION-12 revised) (German) modified | Keller | 2013 | Germany | 40 patients (cardiovascualr prevention; range age, 35-78 yrs, 65% female); 15 general practitioners (range age, 44-56 yrs, 33% female) | *see Elwyn et al., 2005* | *see Elwyn et al., 2005* | a-c) n/a |
| ON-Code | Alby | 2021 | Italy | 19 patients (various cancers; 10 native Italians: Mean age =64.4 yrs, 8 female and 9 non-natives: Mean age =43.11 yrs, all female). 3 providers (oncologists) | 15 items, 7 subscales: Doctor activities per consultation stage (opening, anamnesis, cancer presentation, cancer diagnostic assessment, treatment recommendation, outline of future actions, closing); Patient’s (and companion's) initiatives; Doctor-patient misalignments; Interruptions per consultation stage; Accountability and participants' expressions of trust; Markers of uncertainty in doctor’s talk; Markers of emotions in doctor’s talk. Items assessed in video-recordings transcribed according to conversation analytic conventions | Doctors’ patient-centered communication practices, in particular when there are linguistic and cultural differences between the speakers; definition NR | a) communicative dimensions grounded in emic notions of patient-centered practices, evidence and definitions from the literature; b-c) NR |
| Instrument name (acronym) | First author | Year | Country | Sample | Behaviors assessed: # items, (sub)scales, behaviors NIET | Construct & its definition | Development process a) how was construct developed;  b) item generation and selection;  c) pilot test |
| OnePass Coding System | McMaster | 2015 | USA | 1 of 2 standardized patient "characters" played by 6 actors (1 patient was a smoker considering quitting, the other was overweight and concerned about developing diabetes, both aged 40–60, both female) | 23 items (19 questions, 4 ratios): Set agenda, open questions, affirm client, reflective listening, action reflections, evoke change talk, elicit importance, elicit confidence, address values, respond to affect, roll with resistance, avoid advice, provide menu, support autonomy, explore/guide/choose, summarize, arrange follow-up, demonstrate MI, overall conduct; talk time, reflection ratio, open–closed ratio, complex simple | Motivational interviewing; defined as: 'A person-centered, directive method of talking about change, which uses specific techniques such as open questions and reflective listening' | a-c) NR |
| Paediatric Dental Triadic Interaction Coding Scheme (PaeD-TrICS) | Yuan | 2019 | UK | 6 standardized patients (both aged 40–60, both female). 5 providers (3/5 dentists, 2 extended dental duty nurses; 4/5 male) | 45 items; 2 subscales: Verbal dental professional/parent behaviors (e.g., social talk, information giving, praise, reassurance, offer for questions). Non-verbal behaviors (e.g., touch directing, procedure demonstration). Child behaviors (e.g., speech yes/no/other, dental talk, crying/groaning, laugh, hide face/mouth, agreement, shakes head) | Verbal and non-verbal interaction within a triad of dental professional-child -parent; definition NR | a) preview of 4 video observations and familiarize with related instrument (SABICS); b) supplement an existing related coding instrument (SABICS) with field observation notes, literature evidence and clinical/behavioral evidence; c) the draft coding scheme was pilot-tested on 4 randomly selected observations; At the final stage the PaeD-TrICS was discussed with the whole research team to ensure the representation of the full range of potential behaviors of interest before implementing the coding process |
| Parental Engagement Scale (PES) | Kearney | 2011 | USA | 38 patients (congenital conditions, genetic disorders, other; age, <1 year, n=18, 1–3 yrs, n = 5, 3–5 yrs, n=5, 6–12 yrs (n=4), 13-18 yrs, n=6; 42% female ); Pediatric advanced care providers (N = NR) | # items NR. 3 dimensions: 1. Information-centered dialogue, 2. Insightful participation, 3. Achievement of a collaboratively agreed-upon plan | Parental engagement (i.e., a psycho-behavioral construct denoting effective participation) in decision making and planning for seriously ill children during pediatric palliative care consultations | a) literature review and deductive conceptual reasoning; b) content analysis of consultation narratives, 2 researchers refined construct by grouping categories and identifying observable indicators of behavior for each category, and 3 clinical experts in pediatric palliative care checked content validity; c) 2 researchers applied scoring |
| Instrument name (acronym) | First author | Year | Country | Sample | Behaviors assessed: # items, (sub)scales, behaviors NIET | Construct & its definition | Development process a) how was construct developed;  b) item generation and selection;  c) pilot test |
| Patient Involvement and Collaboration in Emergency care Teams (PIC-ET) tool | Dubois | 2023 | Sweden | Patients n/a; Providers NR (17 video-recorded simulated emergency scenarios) | 22 items, 5 dimensions (relationship, sharing power, information exchange, safe and caring environment, social circumstances) | Patient Participation and collaboration. Patient participation definition: Common elements include empathy, respect, engagement, relationship, communication, shared decision-making, holistic focus, individualized focus, and coordinated care | a) literature review; b) observable behaviors identified and grouped by two authors from published observation tools, qualitative studies, (group) interviews, and simulated emergency team scenarios; c) pilot coding by 2 raters on two recorded emergency team scenarios, resulting in final modifications |
| Patient-centered Behaviour Coding Instrument (PCBI) | Zandbelt | 2005 | Netherlands | 323 patients (visiting internal medicine out-patient clinic; Mean age =53 yrs; 58% female). 15 providers (physicians at general internal medicine; Mean age =38 yrs; 53% male) | 19 items, 2 subscales: facilitating behaviors (11, e.g. emotional reflection, verbal encouragement), inhibiting behaviors (8 items, e.g. premature advice, trivializing); subject area of each expression (medical, psychosocial, other) also coded | Patient-centered behavior; defined as: 'Communication in which the patients’ point of view is actively sought by the physician' | a) literature review and expert discussions; b) through consensus-meetings about codes, descriptions of behaviors were refined; c) 15 consultations were coded and discussed by two coders to refine coding rules and instructions |
| Patient-Centered Communication Coding System (PCCCS) | Ledoux | 2013 | USA | 30 patients and their parents (children with childhood obesity; Mean age =34 yrs (parent) /14 yrs (child); 100% female (parent) /83% female (child); 83% Hispanic). 5 providers (health advisors receiving PCC training; Mean age =7.4 yrs) | 16 items, 3 subscales: Positive PCC (6, broad open-ended questions); Negative PCC (6, e.g., interruptions); Overall communication style (4) | Patient-centered communication; defined according to Person-Centered Theory, 'which posits that change occurs when patients who are anxious ambivalent for change are met with therapist expression of empathy, genuineness, and unconditional positive regard' | a) adapted from a validated Motivational Interviewing (MI) coding system, the MI Treatment Integrity (MITI); b) the authors based adaptations on Person-Centered Theory, the Expert Committee recommendations for PCC review of a sub-sample of audio files by co-investigators for common communication techniques employed by the HAs, and feedback from an expert panel of child obesity prevention researchers; c) final revisions were made during coder training |

| Instrument name (acronym) | First author | Year | Country | Sample | Behaviors assessed: # items, (sub)scales, behaviors NIET | Construct & its definition | Development process a) how was construct developed;  b) item generation and selection;  c) pilot test |
| --- | --- | --- | --- | --- | --- | --- | --- |
| Pediatric Emergency Discharge Interaction Coding Scheme (PEDICS) | Curran | 2017 | Canada | 329 patients and their parents (visiting pediatric emergency department). 17 providers (8/17 physicians, 9 research nurses) | 41 items, 11 subscales: Introduction (5), tests (5), medications given in ED (3) , discharge (4), diagnosis (4), treatment plans (5), medication for home (8), social concerns (2), follow-up (3), clarification (1), conclusion (1). Items assessed in intervals within a 4 hrs observation block | Discharge communication; defined as: 'The exchange of information to inform caregivers about the diagnosis of their child, the treatments received in the ED and plan for follow-up after discharge' | a) literature review; b) interdisciplinary research team reviewed identified behaviors and developed and revised a coding scheme; c) a research assistant shadowed 3 providers to test how behaviors occurred in practice |
| Pediatric Oncology Prognostic Communication (POPC) codebook | Kaye | 2021 | USA | 17 patients and their parents (children with advanced cancer; 9/17 aged >11; 11/17 female; 15/17 white). 6 providers (pediatric oncologists; 3/6 female; all white; 2/6 <5 yrs in practice) | 6 items, no subscales: assessing prognostic understanding, prognostic uncertainty, disease changing for the worse, best- and worst-case scenarios, survival time, curability (3 sub-codes: curability, clarity, population vs. individual) | Communication strategies to share prognostic information; definition NR | a) literature, and standards and guidelines for communication with adult (cancer) patients; b) 6 a priori codes were identified; c) 5 researchers independently pilot tested the codebook across a series of medical dialogue recordings until consensus was reached, and met to modify the code book to improve dependability, confirmability, and credibility of independent codes |
| Perioperative Child–Adult Medical Procedure Interaction Scale (P-CAMPIS) | Caldwell-Andrews | 2005 | NR | 45 patients and their parents (children undergoing elective surgery and general anesthesia; 66% male; Mean age =5.34 yrs (range 2-12); 73% White, 10% African American, 7% Hispanic, 10% other). Providers (various medical staff, including anesthesiologist and nurses) | 40 items, 4 subscales: Adult-to-adult communication (5), adult-child communication (18), either (1), child vocalizations (9), nonverbal behavior (7) | Verbal and nonverbal interactions between children, parents, and medical personnel in the perioperative setting; definition NR | a) repeated observations of 15 of video recordings, to identify recurring verbal and nonverbal behaviors that occurred during the children’s, parents' and medical personnel's interactions, assessment of codes from the existing CAMPIS (modify, delete, retain), discussion with multidisciplinary team including experts in anesthesiology, pediatrics, child psychiatry, child psychology, and child development; b) a preliminary list of codes, examples and explanations were developed; c) 10 transcriptions were used to explore the fit of the preliminary codes |
| Instrument name (acronym) | First author | Year | Country | Sample | Behaviors assessed: # items, (sub)scales, behaviors NIET | Construct & its definition | Development process a) how was construct developed;  b) item generation and selection;  c) pilot test |
| Person Centered Behavior Inventory (PCBI) | Lann-Wolcott | 2011 | USA | 20 patients (long-term care residents with dementia; age range 69-97 yrs (M =82.9); 15 White females, 4 White males, 1 African American male). 52 providers (62% certified nursing assistants, 38% other staff; 83% female; 68% white, 30% African American, 6% other; Mean age = 31.6 yrs (range 21-54); mean yrs experience =7.5 | 19 items, 2 subscales: Verbal categories (11; e.g., shows approval, back-channel responses, and giving choices) and nonverbal categories (8; e.g., resident-directed eye gaze, adjusting to resident’s pace, and proximity). Items assessed in blocks of 30-second intervals within full interaction | Person-centered caregiving in gerontology; defined as: '(a) being in relation, which refers to the importance of promoting the resident’s relationships, especially with caregivers; (b) being in a social world, which refers to the importance of recognizing and affirming the resident’s “goals in life,” which are often embodied in her/his life history; (c) being in place, which refers to the context of caregiving, including organizational aspects; and (d) being with self, which refers to the importance of recognizing the resident’s values | a) literature review (reported elsewhere, for original version only); b) literature review and pilot study (reported elsewhere, for original version only); c) NR |
| Physician Recommendation Coding System (PhyReCS) | Scherr | 2017 | USA (presumed) | 257 patients (men undergoing prostate biopsies; Mean age =63.2 yrs; 70% white, 26% black, 4% other). 47 providers (Mean age =33.2 yrs; 80% male; 68% white, 7% white, 25% other) | 1 item | The strength of physician recommendations during appointments; defined as 'How physicians portrayed each treatment option during the clinical appointment as a whole' | a) NR; b) subset of transcriptions used to develop, in an iterative process, a 5-point scale. The boundaries of each recommendation score were determined through repeated application and discussion; c) NR |
| Problem, Analysis or Assessment, and Solution (Pas® System) | Van Mil | 1997 | Netherlands | n/a | 15 items, 3 subscales (Patient questions (5; e.g., 'difficulties with ...'); provider analysis/assessment of the nature of the question (6; e.g., choice of treatment), provider solution offered (4; e.g., referral) | Patient questions about medication and pharmacist assessment of question and solution offered; definition NR | a) NR; b) 36 pharmacists were asked to describe questions asked by their patients about medicines, together with their answers. 60 question-answer complexes were received and analyzed, focusing on patient problems, pharmacists' assessment, and solutions offered. The resulting coding system was then discussed with the pharmacists and adjusted based on their suggestions; c) NR |

| Instrument name (acronym) | First author | Year | Country | Sample | Behaviors assessed: # items, (sub)scales, behaviors NIET | Construct & its definition | Development process a) how was construct developed;  b) item generation and selection;  c) pilot test |
| --- | --- | --- | --- | --- | --- | --- | --- |
| Process of Interactional Sensitivity Coding in Healthcare (PISCH) | Sabee | 2015 | USA | 52 patients (58 female; 67% White, 14% Hispanic, 15% Black, 4% Asian). 52 providers (n=30 primary care physicians and n=22 endocrinologists; 77% male; yrs of practice range: 3-31) | # items NR. Exchanging information, fostering relationships, managing uncertainty, or meta-communication, recognizing and responding to emotions, making decisions, and enabling self-management. Items assessed in 'a segment of transcript that includes at least 3 contiguous turns, has an identifiable activity, starts and stops with an identifiable change in the activity, and in which each participant must receive a response that is not a receipt token at least once' | Interactional Sensitivity; Defined according to Trees (2000) as the 'active involvement in a conversation by a relational partner in which he or she responds appropriately to his or her partner’s needs in a smooth, synchronized manner' (p. 243); Includes three dimensions: (a) conversational involvement, or attentiveness to the conversation; (b) appropriateness of response given the context of, the situation, and (c) interactional synchrony, or synchronous, coordination of interpersonal communication; in direct reference to an interactional partner, and suggests that partners might be aware of and centered on each other in an interaction | a) the authors defined the characteristics of interactional sensitivity between physicians and patients; identified activities where patients and providers are collaboratively engaged when interacting; operationalized patient-centered communication existing conceptual framework and interviews with cancer patients and experts; b) unmotivated looking was used to develop a PCC coding scheme from a sample of medical visits, which was then quantitatively coded; experts reviewed the scheme to test face validity, intercoder reliability was assessed for 10% of the interactions among multiple coders; c) NR |
| Refined Cue Utilization and Engagement in Dementia (CUeD) | Liu | 2020 | USA | 25 patients (Dementia; Mean age =84.6 yrs (range: 64–96); 60% female; all White, 92% non-Hispanic). 29 providers (Nursing home General staff; Mean age =34.9 yrs (range: 19–79); 83% female; 79% non-Hispanic, 72% White) | # items NR. Original CUED codes: positive and negative staff and resident verbal behaviors; Refinement: addition of staff nonverbal behaviors; staff negative behaviors, resident nonverbal behaviors. # items NR, 4 parts: Resident intake process (Structure of intake episode, Structure of mealtime); Caregiver & Resident verbal behaviors (Positive behaviors, Negative behaviors); Caregiver nonverbal behaviors (Modification of resident functional/cognitive ability, Modification of care approaches, Modification of dining environment, Negative behaviors); Resident nonverbal behaviors (Chewing or swallowing difficulty, Functional impariment, Resistive behaviors, Positive/Neutral behaviors) | Dyadic behaviors during mealtime interactions; whereby an 'intake episode' is defined as: 'the process of getting a bite of solid food or a drink of liquid food from the plate/tray/cup, putting it into the mouth and chewing and swallowing it' | a) NR; b) nonverbal behaviors added to the original measure (CUED) using literature reviews and an existing framework (the person-centered mealtime care philosophy); c) NR |
| Instrument name (acronym) | First author | Year | Country | Sample | Behaviors assessed: # items, (sub)scales, behaviors NIET | Construct & its definition | Development process a) how was construct developed;  b) item generation and selection;  c) pilot test |
| Resident-centered Assessment of Interactions with Staff and Engagement tool (RAISE) | Snow | 2018 | USA | Patients (aged care, in short-term revalidation or long-term care). Providers (aged care staff) | # items NR. For staff or resident, coding of: who was near the observation target individual, specific staff activities, specific resident activities, quality of staff-resident interaction from the perspective of PCC best clinical practices, emotional tone of a staff-resident interaction, and the observation target’s social role in the interaction. Additional coding of level of resident engagement. Items assessed in 5sec. time samples within total of 20min. of interaction | Person-centered care in staff-resident interactions; whereby interaction was defined in accordance with communication theory (Benokraitis, 2015). Emotional tone was defined in accordance with the two dimensional model of emotion (Plutchik, 2001; Schacter & Singer, 1962). PCC best clinical practices, particularly dementia care communication practices, were defined in accordance with dementia care principles (Smith et al., 2011) | a) needs assessment (ethnographic observations), literature review of existing quantitative survey instruments, and open ended-ended interviews and closed-ended surveys, and structured observation tools assessing PCC constructs; b) iterative meetings to develop nine specific observation variables for the preliminary RAISE; c) eight chronological but not necessarily contiguous piloting and refinement cycles. Cycles comprised one or two facility visits, with instrument revision taking place after each visit |
| Rochester Participatory Decision-Making Scale (RPAD) | Shields | 2005 | USA (presumed) | 5 standardized patients (3/5 female). 100 providers (primary care physicians; Mean age=45 yrs; 77% male) | 9 items, no subscales | Participatory decision making; defined as: 'A deliberative model in which physicians elicit and respect patients’ values, but physicians also offer expertise and recommendations, sometimes using persuasion to adopt healthier options if there is not initial consensus.Thus, participatory decision making consists of 2 processes: expert problem solving and decision making' | a) based on previous literature; b) by incorporating items proposed by Braddock et al. (1999); c) pilot testing on 10 audio recordings. Items that were never coded, were discontinued. New items were added, followed by another round of pilot testing |
| Rochester Participatory Decision-Making Scale (RPAD) (Chinese) | Guo | 2020 | China | 200 patients (diagnosis NR; Mean age=32.23 yrs, range, 18-49); 10 gynecologists (range age, 27-57 yrs, 8/10 female) | *See Shields et al., 2005* | *See Shields et al., 2005* | a-c) n/a |

| Instrument name (acronym) | First author | Year | Country | Sample | Behaviors assessed: # items, (sub)scales, behaviors NIET | Construct & its definition | Development process a) how was construct developed;  b) item generation and selection;  c) pilot test |
| --- | --- | --- | --- | --- | --- | --- | --- |
| Roter Interaction Analysis System (RIAS) | Roter | 2002 | USA | NR | 40 mutually exclusive and exhaustive categories of talk assigned to each speaker in the medical encounter, with a handful of codes unique to a provider or patient. Each code is assigned to a discrete statement that captures a complete thought and to which a classification may be assigned. These statements may vary in length from a single word to a simple sentence but is limited to conveying a single thought or item of interest. RIAS category codes are defined by form and content (task or affective) | Medical exchange, divided into socio-emotional and task-focused elements. Physicians' task behaviors defined as 'the performance of medical functions (e.g., data gathering, tests, procedures), physical exam, and patient education and counseling'. Physicians' socio-emotional behaviors defined as 'exchanges with explicit socio-emotional content related to building social and emotional rapport'. Patients' task behaviors defined as: 'question-asking and information giving ("need to know and understand")'. Patient social-emotional behaviors defined as: 'expressions of concern, optimism, empathy, laughter/joking, an social chit-chat' | a) derived from Bales’ Interaction Process System, b-c) NR |
| SDM-18 | Salyers | 2012 | USA | 186 patients (consumers in community mental health centers; Mean age 43.6 yrs; 52% male; 54% Caucasian, 39% African American, 7% other). 8 providers (5 psychiatrists, 3 nurse practitioners) | 15 items, 3 subscales (basic decisions (2); intermediate (5); complex (9)). Consumer’s role in decision making, consumer’s context, the clinical nature of the decision, alternatives, non-medication alternatives, pros and cons, uncertainties or the likelihood of success, assessment of the consumer’s understanding, consumer’s desire to involve others in the decision, and assessment of the consumer’s preferences. Additional ratings: who initiated each element, level of agreement b/w patient and provider | Shared Decision-Making; defined as: 'A collaborative process between a provider and a consumer of health services that entails sharing information and perspectives and coming to an agreement on a treatment plan' | a) a codebook was developed from published descriptions, codebook developed by Braddock and colleagues was reviewed; b) a team of a psychologist, psychiatrist, health communication expert, and two research assistants read transcripts, applied codes, and engaged in an iterative process that included consensus discussions and the addition of new elements and modifications to the rating system; c) NR |

| Instrument name (acronym) | First author | Year | Country | Sample | Behaviors assessed: # items, (sub)scales, behaviors NIET | Construct & its definition | Development process a) how was construct developed;  b) item generation and selection;  c) pilot test |
| --- | --- | --- | --- | --- | --- | --- | --- |
| Segue framework | Makoul | 2001 | USA | 16 patients (assumed; video sample) and 46 patients (assumed; audio sample); 16 providers (assumed; video sample; from GP and rehabilitation medicine) 46 providers (assumed; audio sample; from various specialties) | 25 items + 7 optional items; 17 focused on content, 8 focused on form; 5 sub-categories: Set the stage, Elicit information, Give information, Understand the patient's perspective, End the encounter | Medical communication tasks; definition NR | a) research and education literature; b) prioritization exercise performed by GPs and residents in training, as well as interviews with patients c) the preliminary list was used for 2 yrs in education and refined (items added and removed); checks with (resident/) physicians, students, standardized patients, instructors and researchers indicated that tasks were understood as intended; finally, coders applied the framework to 500 videotaped primary care interactions to check if all behaviors are performed and relevant: 20/23 tasks were observed in a majority of encounters |
| Siminoff Communication Content & Affect Program (SCCAP) | Siminoff | 2011 | USA (presumed) | 1800 patients and/or family members, divided in 3 samples (Sample 1: N=420 (early stage breast cancer); Sample 2: N=1200 (family of deceased patients, tissue requests); Sample 3: N=180 patients (early stage breast cancer) and N=139 family members). Providers (only reported for sample 3: N=39 oncologists) | 29 items, 3 subscales: Verbal: communication types (12, i.e., influence, confirmation, disconfirmation); Content themes (12 options; e.g., treatment, prognosis). Nonverbal: expressions of speech and affect (6) | Instrumental and Relational communication; 'Communication content' defined as: 'denoting generally agreed upon meanings in the shared (i.e., verbal) language of the interactants'. 'Relational communication' defined as: 'reflecting identifiable affective qualities of interaction, embedded in verbal messages and nonverbal cues expressed by the interactants' | a) using various communication theories; b) only reported for one dimension of the instrument: using a previously validated typology as a guide, the authors listened to audiotapes of health care interactions and identified relevant confirmation, disconfirmation messages; c) NR |

| Instrument name (acronym) | First author | Year | Country | Sample | Behaviors assessed: # items, (sub)scales, behaviors NIET | Construct & its definition | Development process a) how was construct developed;  b) item generation and selection;  c) pilot test |
| --- | --- | --- | --- | --- | --- | --- | --- |
| St Andrews Behavioural Interaction Coding Scheme (SABICS) | Zhou | 2012 | UK | 55 patients for applicability and reliability testing (children; age range 3-5 yrs; 51% female) and 36 patients for discriminant validity testing (initially anxious children; age rage 3-5 yrs; 61% male). 19 providers for applicability and reliability testing (all female dental nurses) and 6 providers for discriminant validity testing (all female nurses) | 48 items, 3 subscales: protocol behaviors (6, e.g. introduction, open mouth check); nurse behavior (29; e.g. instruction, information-giving); child behavior (13, e.g., shakes head, says yes/no) | Nurse-child interaction in terms of encouraging cooperative child behavior; defined as: 'Dental staff’s encouragement-centered interaction approach that supports the effective communication in pediatric dentistry' | a-b) 10 team meetings to develop behavioral code definitions and select relevant behavior from literature and field observations; c) initial coding scheme first tested on 10 interactions; Iterative process applied to refine instrument further based on coding interactions |
| State Space Grids | Gainforth | 2019 | UK | 6 patients (Smokers wanting to quit). 2 providers (Behavioral support practitioners) | # items NR. Practitioner verbal behavior (e.g., ‘facilitate goals setting’). Client statements (e.g., ‘sets goals’). | Behavioral support provider patient interaction; defined as: 'Practitioners’ delivery of and clients’ response to Behavioral Change Techniques; Involves multiple, potentially interacting, component behavior change techniques (BCTs) and modes of delivering these techniques. BCTs are defined as: 'observable, irreducible, and replicable ‘active ingredients’ of an intervention that aim to alter or redirect mechanisms of action to change behavior' (Michie & Johnston, 2011; Michie et al., 2016)' | a) literature review; b) to develop the coding frameworks, the smoking cessation taxonomy was adapted to account for the verbal statements made by practitioners (Michie et al., 2011) and a framework for reliably categorizing clients’ verbal statements in smoking cessation consultations was adapted (Gainforthet al., 2016); c) NR |
| Taxonomy of Verbal Response Modes (VRM) | Stiles | 1979 | USA | 52 patients (Primary care conditions; 73% (16-45y), 27% (46-75y); 60% black). 19 providers (General medicine/primary care; 18/19 male, 18/19 white) | *See Stiles et al., 1979* | *See Stiles et al., 1979* | a) an existing theoretical taxonomy of interaction categories called 'verbal response modes'; b) based on the theoretical taxonomy; c) NR |
| Instrument name (acronym) | First author | Year | Country | Sample | Behaviors assessed: # items, (sub)scales, behaviors NIET | Construct & its definition | Development process a) how was construct developed;  b) item generation and selection;  c) pilot test |
| Taxonomy of Verbal Response Modes (VRM) | Stiles | 1982 | USA | 115 patients (all female adults; 86% (16-45y), 14% (46-75y); 58% black, 42% white). 14 providers (9/14 house officers, 5/14 attending physicians; 4/14 female) | 8 items, no subscales: Disclosure, question, edification, acknowledgement, advisement, interpretation, confirmation, reflection. For each of these response modes, both the grammatical form and the intended function are coded | Verbal interactions/exchanges; NR, reported elsewhere (Stiles 1978) | a-c) n/a |
| Verona Coding Definitions of Emotional Sequences (VR-CoDES) | Eide | 2011 | Norway | 12 patients (fibromyalgia or chronic musculoskeletal pain; Mean age =43.6 yrs (range: 26–60); 10/12 female). Providers (NS subsample from N=5 clinical nurse specialists; 4 women) | *See Zimmermann et al., 2011* | *See Zimmermann et al., 2011* | a-c) n/a |
| Verona Coding Definitions of Emotional Sequences (VR-CoDES) | Vatne | 2010 | Norway | 11 patients (leukemia (n=7), lymphoma (n=2), sarcoma (n=2); Mean age =8.4 yrs (range: 6-12); 8 female). Providers (nurse, physician) | *See Zimmermann et al., 2011* | *See Zimmermann et al., 2011* | a-b) NR, c) the two coders and a communications expert examined the differences between the coding of the two coders based on the coding of 15 consultations, and the nature of the statements that were a source of disagreement were discussed in-depth and specified. It was concluded that there was a need for child-specific definitions and specifications of the VR-CoDES manual which would involve additions to, rather than changes in, the original manual |
| Verona Coding Definitions of Emotional Sequences (VR-CoDES) | Wright | 2012 | UK | 13 patients (in dentistry; 8/13 female; all 16 yrs and older, 7/13 aged > 45yrs). 2 providers (1 male dentist, 1 female hygienist (plus dental assistants)) | *See Zimmermann et al., 2011* | *See Zimmermann et al., 2011* | a) developed and validated in previous research; b) developed and validated in previous research; c) NR |

| Instrument name (acronym) | First author | Year | Country | Sample | Behaviors assessed: # items, (sub)scales, behaviors NIET | Construct & its definition | Development process a) how was construct developed;  b) item generation and selection;  c) pilot test |
| --- | --- | --- | --- | --- | --- | --- | --- |
| Verona Coding Definitions of Emotional Sequences (VR-CoDES) | Zimmermann | 2011 | Italy (multiple countries involved in development) | 20 patients (psychiatric outpatients); providers NR | 8 items. 7 for patient elicited expressions of cues, and 1 for concerns | Cues and concerns; 'Concern' defined as: 'An unambiguous expression of an unpleasant current or recent (negative) emotion where the emotion is explicitly verbalized, either with a stated issue of importance for the patient; or without a stated issue'. 'Cue' is defined as: 'A verbal or nonverbal hint which suggests an underlying unpleasant emotion but lacks clarity'. 'Health provider-elicited cues/concerns are' defined as: 'Coherently and logically connected with the previous health provider turn'. 'Patient-elicited cues/concerns' defined as: 'Initiatives of the patient to direct the health provider’s attention, sometimes unwittingly, to emotionally loaded issues, hitherto in the visit ignored or insufficiently discussed or explored, without being expected to do so' | a) through literature review, consensus meetings, and coding exercises; b) through alternating group discussions, individual and group coding exercises; c) a training package for coders was prepared and an inter-rater reliability test was carried out on 20 psychiatric outpatient consultations |
| Verona Coding Definitions of Emotional Sequences (VR-CoDES) (Chinese) | Yin | 2019 | China | 2 simulated patients (graduate psychology students). 75 providers (fourth-year medical students; 55% female) | *See Zimmermann et al., 2011* | *See Zimmermann et al., 2011* | a-c) NR |
| Verona Coding Definitions of Emotional Sequences for client cues and concerns (VR-CoDES-CC) | Vijfhuizen | 2017 | Australia | 24 clients (pet owners; 80% canines, 20% felines); 15 providers (veterinary medicine; age range: 18–50 yrs; 12/15 female) | *See Zimmermann et al., 2011* | *See Zimmermann et al., 2011* | a-b) NR; c) a pilot study on 6 consultations was performed to determine any necessary modifications to the original VR-CoDES. Based on discussing challenges in coding, new notations were introduced to identify the perspective and direction of cues and concerns. Also, reminders were created to assist with coding (without making changes to the codes), based on frequently encountered expressions that were challenging to categorize |
| Instrument name (acronym) | First author | Year | Country | Sample | Behaviors assessed: # items, (sub)scales, behaviors NIET | Construct & its definition | Development process a) how was construct developed;  b) item generation and selection;  c) pilot test |
| Verona Coding Definitions of Emotional Sequences for health provider responses (VR-CoDES-P) | Del Piccolo | 2011 | Italy | 104 patients (psychiatric disorders); 16 providers (9/16 psychiatrists, 7/16 residents; 9/16 male; mean age = 39 yrs) | 17 items (categorized along 2 dimensions: Explicit/Non-explicit, Providing/reducing space) | Provider responses to explicit (concern) or subtle (cue) expression of negative emotion. 'Concern' defined as: 'Explicit naming of negative emotion'. 'Cue' defined as: 'Use of more ambiguous or vague expressions of negative emotion' | a-b) NR; c) a pilot study on 6 consultations was performed to determine any necessary modifications to the original VR-CoDES. Based on discussing challenges in coding, new notations were introduced to identify the perspective and direction of cues and concerns. Also, reminders were created to assist with coding (without making changes to the codes), based on frequently encountered expressions that were challenging to categorize |
| Verona Coding Definitions of Emotional Sequences for health provider responses (VR-CoDES-P) | Vijfhuizen | 2017 | Australia | 24 clients (pet owners; 80% canines, 20% felines); 15 providers (veterinary medicine; age range: 18–50 yrs; 12/15 female) | *See Zimmermann et al., 2011 & Del Piccolo et al., 2011* | *See Zimmermann et al., 2011 & Del Piccolo et al., 2011* | a) position paper describing development process, small group worked on conceptual framework; b) provider talk system was organized around 3 axes; c) pilot feasibility study, updated framework was discussed and tested again on 3 transcripts and one video by Verona Network |
| Verona Coding Definitions of Emotional Sequences for health provider responses (VR-CoDES-P) | Wright | 2012 | UK | 13 patients (in dentistry; 8/13 female; all 16 yrs and older, 7/13 aged > 45yrs). 2 providers (1 male dentist, 1 female hygienist (plus dental assistants)) | *See Zimmermann et al., 2011 & Del Piccolo et al., 2011* | *See Zimmermann et al., 2011 & Del Piccolo et al., 2011* | a) developed and validated in previous research; b) developed and validated in previous research; c) NR |
| Verona Coding Definitions of Emotional Sequences for health provider responses (VR-CoDES-P) (Chinese) | Yin | 2019 | China | 2 simulated patients (graduate psychology students). 75 providers (fourth-year medical students; 55% female) | *See Zimmermann et al., 2011 & Del Piccolo et al., 2011* | *See Zimmermann et al., 2011 & Del Piccolo et al., 2011* | a-c) NR |
| Instrument name (acronym) | First author | Year | Country | Sample | Behaviors assessed: # items, (sub)scales, behaviors NIET | Construct & its definition | Development process a) how was construct developed;  b) item generation and selection;  c) pilot test |
| Verona Medical Interview Classification System (VR-MICS) | Del Piccolo | 2004 | Italy | 238 patients (Mean age = 45 yrs). 6 providers (GPs; Mean age =46 yrs; all male; mean experience =17 yrs) | 18 items, 8 subscales: Data gathering (e.g., Physician closed-ended and open-ended questions; Patient asking for opinion); Data gathering and relationship building (e.g., checking, transitions); Relationship building (e.g., Reassurance); Information giving (e.g., giving Instruction); Cues, Statements (e.g., social, Psychological); Patient speech act categories (e.g., positive talk, bid for repetition); Physician speach act categories | Verbal interaction including speech acts and content between physicians and emotionally distressed patients with a medical problem; defined as: 'descriptions of what is “done” by the speaker and/or what is said' | a-c) NR |
| Verona Medical Interview Classification System (VR-MICS) | Del Piccolo | 2005 | Italy / UK | Patients (presenting with new health problem; age range: 18–74 yrs). 12 providers (6 Italian GPs: all male; aged 35 -55 yrs; all ≥4 yrs experience; and 6 UK GPs: all male; aged 35-55 yrs; ≥4 yrs experience) | *See Del Piccolo et al., 2004* | *See Del Piccolo et al., 2004* | a-c) n/a |
| Vr-COPE | McDermott | 2021 | UK / USA | 55 clients (visiting veterinary surgeon with their pet, 28 from UK, 27 from USA); providers NR (veterinary surgeons) | 10 items, no subscales: agenda, worries/needs, psychosocial impact, active listening, empathy & support, client point of view, client expectations, structuring, shared understanding/decision making, veterinary surgeon-pet interaction | Relationship centered communication, defined as: a sharing of dialogue and decision-making, characterized by the use of open questions, active listening, making eye contact, including the client’s thoughts and opinions, and demonstrating empathy and understanding of the client’s perspective | a) based on existing measure; b) existing Vr-Cope measure for clinician-patient communication was adapted regarding phrasing, and by adding a 10th element (“Veterinary Surgeon-Pet Interaction”); c) two raters coded 2 pilot consultations, and provided feedback on their experience |

**Note: 1. Articles reporting about the development or (further) development of more than one coding system, are included once for each coding system.**
