# Supplementary material for: Systematic review of observational coding systems to assess patient-healthcare professional interactions
Source: Patient Educ Couns. Author manuscript; Available in PMC 2026 Aug 16. (PMC13477582; doi:10.1016/j.pec.2025.108718)
Supplement: Supplementary file 3 [file NIHMS2172109-supplement-3.docx]

Supplementary Table 1c. General characteristics of included coding systems, ordered by interactional focus (N=98)

| Instrument name (acronym) | | First author | Year^a^ | Validation studies | Construct | Target population | Sample(s) and size, setting | Target 'actor' | Behaviors assessed: # items, domains (if applicable) | Response type(s)^b^ |
| --- | --- | --- | --- | --- | --- | --- | --- | --- | --- | --- |
| **BEHAVIOR CHANGE COUNSELING** | | | |  |  |  |  |  |  |  |
| Assessment of Brief Behavioural Change Counselling’ tool (ABC tool) | Fouche | | 2020 | n/a | Behavior change counseling (brief); definition provided | Primary care providers | NR (standardized patients). | Provider | 22 items; 5 domains: ask, advise, assess, assist, arrange. | Frequency |
| NR (behavior change therapy delivery and receipt) | Hoekstra | | 2022 | n/a | Behavior change counseling: Behavior change technique delivery and receipt; definition provided. | Adults with physical disabilities, counselors delivering physical activity behavior change counseling | 14 patients & 1 provider; chronic spinal cord injuries. | Provider, client | 94 items, 13 domains (e.g., Support strategies; see Supplementary Table 1). | Frequency |
| Indicators of Health Behavior Change (IHBC) | Potempa | | 2023 | n/a | Behavior change counseling: Change talk, insight/pattern recognition, self-efficacy/agency, sustainability, resiliance; definitions provided. | NR | 59 patients & providers NR; health coaching. | Patient | # items NR, 5 domains: Change talk, Insight/pattern recognition, Self-efficacy and independent agency, Sustainability, Resilience. | Frequency, evaluation |
| NR (client responses to practitioner-delivered behaviour change techniques) | Gainforth | | 2016 | n/a | Behavior change counseling: Client verbal statements in response to behavioural change techniques; Definition provided for 'Client statement'. | Smokers, practitioners delivering behaviour support interventions for smoking cessation | 18 patients & providers NR; behavior change. | Patient | 58 items, 5 domains: 'Client' BCTs, Behaviour maximizing self-regulatory capacity or skills, Adjuvant activities, General aspects of the interaction & Statements unrelated to smoking or smoking cessation. | Frequency |
| Health Coaching Index (HCI) | Sohl | | 2021 | n/a | Behavior change counseling: Health coaching skills; definition provided. | Patients visiting health coaches, health coaches (in training) | Standardized patients NR & 42 providers; health coaching. | Provider | 18 items, 4 domains: Long-term vision, designing goals & actions, and self-monitoring, Use a self-discovery process, Match information to client interest and needs, Have a client-centered/mindful approach. | Evaluation |

| Instrument name (acronym) | | | First author | Year^a^ | Validation studies | Construct | Target population | Sample(s) and size, setting | Target 'actor' | Behaviors assessed: # items, domains (if applicable) | Response type(s)^b^ |
| --- | --- | --- | --- | --- | --- | --- | --- | --- | --- | --- | --- |
|  | Motivational Interviewing Skills Code (MISC) | Moyers | | 2003 | Lord 2015 | Behavior change counseling: Motivational interviewing skills; definition provided. | Individuals for whom behavior change is desirable, providers counseling behavioral change. | Range, 19-50 patients & 4-NR providers; 2 different samples; motivational interviewing. | Provider, patient | 46 items 7 domains: Global assessment of provider, Global assessment of patient, Global assessment of interaction, Therapist behaviors, Client behaviors, Therapist adherence to MI method & Talk time ratio. | Frequency, evaluation, duration |
|  | Motivational Interviewing Treatment Integrity (MITI) rating system | McMaster | | 2015 | Forsberg 2007; Forsberg 2008 | Behavior change counseling: Motivational interviewing; definition provided. | NR | Range, 6-60 (standardized) patients & range, NR-27 providers; 3 different samples; motivational interviewing. | Provider | 12 items; ‘Global’ scores & Behavior counts. | Frequency, evaluation |
|  | OnePass Coding System | McMaster | | 2015 | n/a | Behavior change counseling: Motivational interviewing; definition provided. | NR (tool developed to be applied in a "range of settings") | 6 simulated patients; providers NR; behavior change. | Provider | 23 items, 19 questions & 4 ratios (Talk time, Reflection ratio, Open-closed ratio & Complex simple). | Evaluation |
|  | Davis Observation Code (DOC) | Callahan | | 1991 | n/a | Behavior change counseling: Verbal physician behaviours relating to disease prevention, health education, health promotion, compliance checking; definition NR. | Primary care patients and physicians | 60 patients & 31 residents. | Provider, Patient | 20 items. | Frequency |
|  | 5 As for Physical Activity | Carroll | | 2011 | n/a | Behavior change counseling; definition provided | Patients with low physical activity and their treating clinicians | 135 patients & 28 physicians; Internal/family medicine. | Providers, patients | 5 items. | Frequency |
|  | 5a-DOC | Lawson | | 2009 | n/a | Behavior change counseling; definition provided | Primary care outpatient visits and primary care clinicians | Development: 186 patients & 5 physicians; Evaluation: 739 patients & 28 physicians; Primary care. | Provider | 5 items. | Frequency |
|  | Behaviour Change Counselling Index (BECCI) | Lane | | 2005 | n/a | Behavior change counseling; definition provided | Patients undergoing and practitioners delivering/training for behavior change counseling. | 183 patients & providers NR; 9 different samples (details NR); medical setting NR. | Provider | 11 items, 4 domains: agenda setting and permission seeking; the how and why of change in behavior; the consultation as a whole; and talk about targets. | Evaluation |
| Instrument name (acronym) | | | First author | Year^a^ | Validation studies | Construct | Target population | Sample(s) and size, setting | Target 'actor' | Behaviors assessed: # items, domains (if applicable) | Response type(s)^b^ |
|  | **DECISION MAKING** | | |  |  |  |  |  |  |  |  |
|  | NR (ambivalence and reluctance in palliative care ) | Gupta | | 2023 | n/a | Decision making: Ambivalence; definition provided. | Patients with serious illness, palliative care clinicians | 70 patients and caregivers & 22 providers; palliative care. | Patient, patient's caregiver | 3 items. | Frequency |
|  | Kin interaction coding system (KINcode) | Laidsaar-Powell | | 2015 | n/a | Decision making: Communication and decision-making behaviors; definition NR. | Oncology patients and family members, oncologists | 72 patients & 59 family members & 18 providers; oncology. | Provider, patient, patient family | 70 items, 4 domains: Family member global role, Family member's behaviors, Oncologist behaviors towards family member, Patient behaviors towards family member. | Frequency |
|  | NR (decision-making around limiting life supporting treatment in neonatal intensive care) | Shaw | | 2023 | n/a | Decision making: Critical care decision making, specifically (1) the first decision point, (2) aspects of any subsequent decision point(s),. and (3) how parents responded; definition NR. | Parents of critically ill children in neonatal care; physicians in neonatal care | Parents of 21 babies; 14 providers; neonatal intensive care. | Provider, patient's parent(s) | 22 items, 3 domains: First decision point, Subsequent decision points, Parents' responses. | Frequency, evaluation |
|  | Brief Decision Support Analysis Tool (DSAT-10) | Stacey | | 2008 | n/a | Decision making: Decision support; definition provided | Generic | 6 standardized patients & 38 providers; various medical settings. | Provider | 10 items relating to 5 elements: decision-making status, knowledge, values/preferences, others' involvement in decision, next steps. | Frequency |
|  | Detail of Essential Elements and Participants in Shared Decision Making (DEEP-SDM) | Clayman | | 2012 | n/a | Decision making: Essential elements of shared decision making; defintion provided. | Patients presented with therapeutic choice and any health professionals presenting choice. | 20 patients & providers NR; advanced breast cancer. | Provider, patient | # items NR. | Frequency, evaluation |
|  | Observing patient involvement (OPTION-5) | Elwyn | | 2013 | Barr 2015; Vortel 2016; Stubenrouch 2016; Kölker 2018; Chen 2020. | Decision making: Essential requirements of SDM when providers make an effort to involve patients in decisions. Definition NR except behaviorally. | Provider and patient making a decision (generic) | Range, 27-311 patients & 8-37 providers; 6 different samples; various settings. | Provider | 5 items. | Evaluation |

| Instrument name (acronym) | | | First author | Year^a^ | Validation studies | Construct | Target population | Sample(s) and size, setting | Target 'actor' | Behaviors assessed: # items, domains (if applicable) | Response type(s)^b^ |
| --- | --- | --- | --- | --- | --- | --- | --- | --- | --- | --- | --- |
|  | Decision Analysis System for Oncology (DAS-O) | Brown | | 2011 | n/a | Decision making: Establishing a shared decision making framework, and providing clear and unbiased information about standard treatments and clinical trials; definition NR. | Oncology patients and oncologists | 143 patients & 46 providers; early-stage breast cancer. | Provider, Patient | 56 items, 5 domains: Establishing the physician-patient team, Following a consultation pathway, Providing information about standard treatments and clinical trials, Promoting clarity, Avoiding coercion. | Frequency, evaluation |
|  | Observing patient involvement in decision making (OPTION-12 revised) | Elwyn | | 2005 | Elwyn 2003; Goss 2007; Weiss 2008; Butow 2010; Kasper 2011; Hirsch 2012; Keller 2013; Stubenrouch 2016 | Decision making: Extent to which clinicians involve patients in decision-making processes; definition NR. | Provider and patient making a decision (generic) | Range, 40-235 patients & range, 4-37 providers; 7 different samples; various medical settings. | Provider | 12 items. | Evaluation |
|  | Elements of Informed Decision Making (IDM) | Braddock | | 1999 | Weiss 2008; Leader 2012 | Decision making: Informed decision making; definition provided. | Providers and patients making decisions in routine care | Range, 123-1057 patients & range, 12-124 providers; 3 different samples; various medical settings. | Provider | 7 items assessing 7 elements (e.g., Patient's role in decision making) & 3 items assessing decision complexity (basic, intermediate, complex). | Frequency, evaluation |
|  | NR (verbal interpersonal clinical problem-solving skills) | Pridham | | 1980 | n/a | Decision making: Interactive clinical problem-solving; definition provided. | Primary care patients; primary care providers (e.g. family physicians, social workers, nurses). | 5 patients & 5 providers; various medical settings. | Provider, patient | 48 items, 16 domains & 7 Phases: Scanning, Formulating; Appraising, Developing willingness/readiness to problem solve, Planning, Implementing & Evaluating. | Frequency |
|  | Parental Engagement Scale (PES) | Kearney | | 2011 | n/a | Decision making: Parental engagement in decision making and planning for seriously ill children during pediatric palliative care consultations. Definition provided for 'parental engagement'. | Families of pediatric palliative patients and providers | 38 patients; providers NR; pediatric advanced care. | Parent of patient | # items NR. 3 domains: Information-centered dialogue, Insightful participation & Achievement of a collaboratively agreed-upon plan. | NR |

| Instrument name (acronym) | | | First author | Year^a^ | Validation studies | Construct | Target population | Sample(s) and size, setting | Target 'actor' | Behaviors assessed: # items, domains (if applicable) | Response type(s)^b^ |
| --- | --- | --- | --- | --- | --- | --- | --- | --- | --- | --- | --- |
|  | Rochester Participatory Decision-Making Scale (RPAD) | Shields | | 2005 | Guo 2020 | Decision making: Participatory decision making; definition provided. | NR | Range, 5-200 (standardized) patients & 10-100 providers; 2 different samples; various medical settings. | Provider | 9 items. | Evaluation |
|  | Content Coding for Contextualization of Care (4C) | Weiner | | 2014 | n/a | Decision making: Physician attention and responsiveness to patient context when planning care; definition provided. | NR | Patients NR & providers NR; internal medicine. | Provider | # items NR, 10 contextual domains. Contextual Factors, Contextual Red Flags, Contextual Probes, Contextualized Plans of Care relating to 1 of 10 domains. | Frequency |
|  | Decision Support Analysis Tool (DSAT) | Guimond | | 2003 | Butow 2010 | Decision making: Providers' use of decision support and related communication skills during clincial encounters; definition NR. | Provider and patient making a decision (generic) | Range, 34-55 patients & range, 20-34 physicians; 2 different samples; various medical settings. | Provider | 22 items, 6 categories of decision support skills & 4 categories of communication skills. | Frequency |
|  | Mappin'SDM | Kasper | | 2012 | Kienlin 2017 | Decision making: Shared decision-making behaviors and results (extent of actual involvement achieved). Definition of SDM NR. Definitions for 'behaviors' and 'results' provided. | NR | Range, 32-40 patients & range, 10-23 providers; 2 different samples; various medical settings. | Provider, patient, dyad. | 15 items, each scored 3 times (for provider, patient, and dyad). | Evaluation |
|  | NR (coding frame for shared decision-making) | Singh | | 2010 | n/a | Decision making: Shared decision-making behaviors; definition provided. | Patients with cancer; cancer specialists | 63 patients & providers NR; oncology. | Provider | 20 items, 6 domains: Establishing the problem, Doctor-patient relationship, Research evidence, Patient perspective, Decision making &Time issues. | Evaluation. |
|  | SDM-18 | Salyers | | 2012 | n/a | Decision making: Shared decision-making; definition provided. | Psychiatric patients and providers | 186 patients & 8 providers; mental health care. | Provider, patient | 15 items, 3 domains (Basic decisions, Intermediate decisions & Complex decisions). | Frequency, evaluation |
|  | Physician Recommendation Coding System (PhyReCS) | Scherr | | 2017 | n/a | Decision making: The strength of physician recommendations during appointments; definition provided. | Patients confronted with & providers involving patients in a preference-sensitive treatment choice. | 257 patients & 47 providers; prostate cancer screening. | Provider | 1 item | Evaluation |

| Instrument name (acronym) | | | First author | Year^a^ | Validation studies | Construct | Target population | Sample(s) and size, setting | Target 'actor' | Behaviors assessed: # items, domains (if applicable) | Response type(s)^b^ |
| --- | --- | --- | --- | --- | --- | --- | --- | --- | --- | --- | --- |
|  | Advance Care Planning Communication Assessment Tool (ACP-CAT) | Yuen | | 2020 | n/a | Decision making: Advance care planning communication skills. Definition provided | Clinicians and trainees conducting or learning to conduct ACP conversations | 2 simulated patients & 29 residents; advanced cancer /Internal Medicine. | Provider | 22 items, 6 subscales (5 for ACP-steps, 1 general communication skills) and 2 summative items. | Frequency, evaluation |

|  | **EMOTIONS** |  | |  |  |  |  |  |  |  |  |
| --- | --- | --- | --- | --- | --- | --- | --- | --- | --- | --- | --- |
|  | Verona coding definitions of emotional sequences (VR-CoDES) | Zimmermann | | 2011 | Vatne 2010; Eide 2011; Wright 2012; Yin 2019; Vijfhuizen 2017 | Emotions: Provider responses to explicit (concern) or subtle (cue) expression of negative emotion. Definitions provided for 'Concern' and 'Cue'. | Patients and providers (not further specified) | Range, 2-20 (simulated) patients; range, NR-75 providers; 6 different samples; various medical settings. | Provider | 8 items, 2 domains (Cues & Concerns). | Frequency |
|  | Measure of Patient-Centered Communication (MPCC) | Shields | | 2005 | n/a | Emotions: Physicians' use of emotion words; definition NR. | NR | 2 Standardized patients & 50 patients & providers; primary care. | Provider | # items NA. Relative total number of emotion words spoken by the physician. | Frequency |
|  | Verona Coding Definitions of Emotional Sequences for health provider responses (VR-CoDES-P) | Del Piccolo | | 2011 | Wright 2012; Vijfhuizen 2017; Yin 2019 | Emotions: Provider responses to explicit (concern) or subtle (cue) expression of negative emotion. Definitions provided for 'Concern' and 'Cue'. | NR | Range, 2-104 (simulated) patients/clients; 2-75 providers; 4 different samples; various medical settings. | Provider | 17 items, 2 dimensions: Explicit/Non-explicit & Providing/reducing space. | Frequency |
|  | **EMPATHY** |  | |  |  |  |  |  |  |  |  |
|  | Empathic and Potential Empathic opportunity (E-PE-O) method | Eide | | 2004 | n/a | Empathy: Empathic opportunities (EO) and potential empathic opportunities (PEO); definitions provided. | Patients NR; oncology providers | 39 patients & 4 providers; Oncology. | Provider, Patient | # items NR: Empathic opportunity, Potential empathic opportunity. | Frequency |
|  | Empathic Communication Coding System (ECCS) | Bylund | | 2002 | n/a | Empathy: Empathic communication, specifically patients' empathic opportunities and physicians' responses to these empathic opportunities; definitions provided. | Patients NR, physicians | 100 patients & 20 providers; Internal medicine. | Provider, patient | 9 items, 2 domains (Patient's empathic opportunity, Physician empathy rating). | Frequency, evaluation |
| Instrument name (acronym) | | | First author | Year^a^ | Validation studies | Construct | Target population | Sample(s) and size, setting | Target 'actor' | Behaviors assessed: # items, domains (if applicable) | Response type(s)^b^ |
|  | **GENERAL COMMUNICATION** | | | |  |  |  |  |  |  |  |
|  | NR (Global Consultation Rating Scale (GCRS) adapted for pediatric subspecialties) | Coburn | | 2020 | n/a | General communication: Adequate provider communication according to Cambridge-Calgary guidelines; definition provided. | Patients, patients' caregivers and providers in pediatric sub-specialties | 99 patients & 18 providers; chronic kidney disease. | Provider | #items NR, 12 domains (e.g., Patient’s perspective; see Supplementary Table 1). | Evaluation |
|  | NR (cognitive strategies used by general practitioners in diagnostic reasoning) | Donner-Banzhoff | | 2017 | n/a | General communication: Cognitive strategies, specifically: inductive foraging, descriptive questioning, triggered routines, hypothesis testing; definitions provided. | Patients visiting GPs/primary care physicians; GPs/primary care physicians | 134 patients & 12 providers; general practice. | Provider, patient | # items NR. | Frequency |
|  | Manchester Observation Code (MOC) for genetic counseling | Liede | | 2000 | n/a | General communication: Communication behavior specific to genetic counseling interviews; definition NR. | People visiting for genetic counseling, genetic counselors | 38 clients /children & 39 providers; genetic counseling. | Provider | 20 items, 4 domains. For counselor expressions: grammatical form, Purpose, Subject, Cue source. | Frequency |

|  | NR (number and type of problems discussed in primary care consultations) | Procter | | 2014 | n/a | General communication: Complex consultations in general practice; definition provided. | Patients visiting a GP; GPs | Development: 13 patients & 2 providers; Reliability testing: 60 patients & 30 providers; Testing: 169 patients & 30 providers; GP. | Provider, patient | 27 items, 2 domains: Issues & Problems/diseases. | Frequency |
| --- | --- | --- | --- | --- | --- | --- | --- | --- | --- | --- | --- |
|  | Liverpool-MAAS | Enzer | | 2003 | n/a | General communication: Consultation competence; definition provided. | Patients visiting a GP and GPs. | Development: 4 patients & 4 providers; Evaluation: 71 patients & 8 providers; general practice. | Provider | 95 items, 6 subscales: Exploring, History taking, Attitudes, Presenting solutions, Structuring interview, Interpersonal skills, Communicative skills. | Evaluation |
|  | NR (coding scheme to identify the content of general practitioner communication) | Di Caccavo | | 2000 | n/a | General communication: Content of general practitioner verbal communication behaviors; definition NR. | General practice patients with psychological complaints; providers (not further specified) | 75 patients & providers NR; psychological symptoms /general practice. | Provider | # items NR, 16 domains (e.g., Mental symptom description; see Supplementary Table 1) . | Frequency |
| Instrument name (acronym) | | | First author | Year^a^ | Validation studies | Construct | Target population | Sample(s) and size, setting | Target 'actor' | Behaviors assessed: # items, domains (if applicable) | Response type(s)^b^ |
|  | Dementia Competent Social Interaction (DSI) and Culturally Competent Social Interaction (CSI) Coding Schemas | Kim | | 2012 | n/a | General communication: Dementia competent and culturally competent social interactions; definition NR. | Korean-American older adults with dementia and direct-care staff | 32 patients & 6 providers; dementia. | Provider | 27 items (DSI) and 17 items (CSI). For DSI: 12 Competent behaviors, 15 Incompetent behaviors. For CSI: 8 Competent behaviors, 9 Incompetent behaviors. | Frequency |
|  | Roter Interaction Analysis System (RIAS) | Roter | | 2002 | n/a | General communication: Dynamics of resource exchange between patients and providers through the medical dialogue; definition NR except behaviorally. | Generic | NR | Provider, patient | 40 items. | Frequency, duration. |
|  | Siminoff Communication Content & Affect Program (SCCAP) | Siminoff | | 2011 | n/a | General communication: Instrumental and Relational communication; definitions provided for 'Communication content' and 'Relational communication'. | NR, but instruments reported to be 'adaptive to multiple health communication contexts' | Range, 180-1200 patients and/or family members & range, NR-39 providers; 3 different samples; oncology. | Provider | 29 items, 3 domains: Verbal communication types, Verbal content themes & Non-verbal expressions of speech and affect. | Frequency, evaluation |
|  | Segue framework | Makoul | | 2001 | n/a | General communication: Medical communication tasks; definition NR. | NR; For providers: 'Different doctors in different settings'. | Range, 16-46 patients & range, 16-46 providers; 2 different samples; various medical settings. | Provider | 25 items & 7 optional items, 2 domains (Content & Form) and 5 sub-domains (Set the stage, Elicit information, Give information, Understand the patient's perspective & End the encounter). | Frequency |
|  | Chronic Pain Coding System (CPCS) | Henry | | 2016 | n/a | General communication: Patient-centered communication, negotations; patient assessments of their physical pain and pain treatment; definition NR except behavorally. | NR | 45 patients & 33 providers; chronic pain. | Provider, patient | 7 major patient/companion utterance themes; 22 subscales. 6 major clinician utterance themes; 21 subscales. | Frequency |
|  | Four Habits Coding Scheme (4-HCS) | Krupat | | 2006 | Jensen 2010; Catani 2018; Bellier 2020 | General communication: Provider Communication skills; definition NR. | Patients and providers (not further specified) | Range, 4-497 (standardized) patients & range, 50-200 providers; 4 different samples; various medical settings. | Provider | 23 items, 4 subscales: Invest in the beginning, Elicit patient’s perspective, Demonstrate empathy, Invest in the end. | Frequency, evaluation |
| Instrument name (acronym) | | | First author | Year^a^ | Validation studies | Construct | Target population | Sample(s) and size, setting | Target 'actor' | Behaviors assessed: # items, domains (if applicable) | Response type(s)^b^ |
|  | NR (the process and content of general practice consultations involving the prescription of antibiotic agents) | Cockburn | | 1987 | n/a | General communication: Successful consultation; definition provided. | Patients with prescriptions for new courses of antibiotic agents; GPs | 177 patients & 56 providers; primary care. | Provider, patient | 12 items, 3 domains: Informational aspects, Mutuality & Strategies to improve knowledge and compliance. | Frequency |
|  | Verona Medical Interview Classification System (VR-MICS) | Del Piccolo | | 2004 | Del Piccolo 2005 | General communication: Verbal interaction including speech acts and content between physicians and emotionally distressed patients with a medical problem; defined as: 'descriptions of what is “done” by the speaker and/or what is said'. | Emotionally distressed patients with medical problem; physicians | Range, NR-238 patients & range, 6-12 providers; 2 different samples; general practice. | Provider, patient | 18 items, 8 domains (e.g., Relationship building; see Supplementary Table 1). | Frequency |
|  | Taxonomy of Verbal Response Modes (VRM) | Stiles | | 1979 | Stiles 1982 | General communication: Verbal interactions/exchanges; definition NR (reported elsewhere). | Patients in medical interviews; providers conducting medical interviews | Range, 52-115 patients & 14-19 providers; 2 different samples; various medical settings. | Provider, patient | 8 items, coding of both grammatical form and intended function. | Frequency, evaluation |
|  | Cancode interaction analysis system (Cancode) | Dent | | 2005 | n/a | General communication: Cancer-specific consultation interaction; definition NR. | Cancer patients and oncologists | 1 simulated patient & 10 providers; oncology. | Provider | 4 domains: Source, Content, Function, Emotion. Items: Verbal, Nonverbal. | Frequency, duration |
|  | **INFORMATION GIVING** | | |  |  |  |  |  |  |  |  |
|  | NR (Complex oral information transfer) | Nordfalk | | 2019 | n/a | Information giving: Complex information transfer; definition provided. | Complex information transfer situations | 34 standardized patients & 17 providers; Multiple Sclerosis. | Provider, patient | 2 items/domains: Count-COPIN (units of provided information), Count-PROPIN (units of information recalled by patient). | Frequency |
|  | Pediatric Emergency Discharge Interaction Coding Scheme (PEDICS) | Curran | | 2017 | n/a | Information giving: Discharge communication; definition provided. | Parents of children visiting the emergency department; pediatric emergency department physicians and nurses | 329 children and parents & 17 providers; emergency medicine. | Provider | 41 items, 11 domains: Introduction, Tests, Medications given in ED, Discharge, Diagnosis, Treatment plans, Medication for home, Social concerns, Follow-up, Clarification & Conclusion. | Frequency, duration |

| Instrument name (acronym) | | | First author | Year^a^ | Validation studies | Construct | Target population | Sample(s) and size, setting | Target 'actor' | Behaviors assessed: # items, domains (if applicable) | Response type(s)^b^ |
| --- | --- | --- | --- | --- | --- | --- | --- | --- | --- | --- | --- |
|  | Medical Interaction Process System (MIPS) | Ford | | 2000 | Ford, 2004 | Information giving: Information transfer in terms of content and relationship in oncologist-cancer patient encounters; definition NR. | Cancer patients, oncologists | Range, 6-20 patients & 5-NR providers; 2 different samples; oncology/NR. | Provider, patient | # items NR. 15 content categories (topic addressed), 16 dependent modes of exchange (process/function of utterance), 14 independent modes, 7 global affective (paralinguistic) categories & 7 non-verbal (kinesic) categories. | Frequency, evaluation |
|  | Pediatric Oncology Prognostic Communication (POPC) codebook | Kaye | | 2021 | n/a | Information giving: Communication strategies to share prognostic information; definition NR. | Children with cancer; pediatric oncologists | 17 children and parents & 6 providers; pediatric oncology. | Provider | 6 items. | Frequency, duration |
|  | Multi-Dimensional Analysis of Patient Outcome Predictions (MD.POP) | Ménard | | 2018 | n/a | Information giving: Verbal discussion of patient outcomes during medical encounters; definition for 'patient outcomes' provided. | NR | 1 simulated patient & 40 providers; oncology. | Provider, patient | 6 items. | Frequency |
|  | NR (Automatic annotation of information-giving in clinical conversations) | Mayfield | | 2014 | n/a | Information-giving; definition provided. | Patients and providers in clinical encounters | 415 people & 45 providers; HIV. | Provider, patient | 3 items. | Frequency |
|  | **NON-VERBAL COMMUNICATION** | | | |  |  |  |  |  |  |  |
|  | Nonverbal communication in Doctor–Elderly Patient Transactions (NDEPT) | Gorawara-Bhat | | 2007 | n/a | Non-verbal communication; definition provided. | Elderly patients, providers NR | 50 patients & providers NR. | Provider | 16 items, 3 domains: Static attributes, Dynamic attributes & Kinesic attributes. | Frequency, evaluation |
|  | NR (Nurse Patient Touch) | Bottorff | | 1994 | n/a | Non-verbal communication; Nurse-patient touch; definition NR. | Patients (not further specified); nurses | 8 patients & 32 providers; oncology nursing. | Provider, patient | 13 items, 2 domains: Interactional context, Touch event. | Frequency, evaluation, duration |
| Instrument name (acronym) | | | First author | Year^a^ | Validation studies | Construct | Target population | Sample(s) and size, setting | Target 'actor' | Behaviors assessed: # items, domains (if applicable) | Response type(s)^b^ |
|  | NR ("Strength of connectional silence" taxonomy) | Bartels | | 2016 | n/a | Non-verbal communication; Silence present during interactions, specifically: connectional silence, invitational silence, neutral silence, disengaged silence; definition provided. | Advanced cancer patients, oncologists | 124 patients (+caregivers) & 41 providers; oncology. | Provider | 10 broad dimensions – 5 lexical & 5 musical. | NA |
|  | Nonverbal Accommodation Analysis System (NAAS) | D'Agostino | | 2011 | n/a | Non-verbal communication; definition NR. | Patients and physicians within medical consultations | 45 patients & providers NR; oncology. | Provider, patient | 10 items. | Frequency, duration |
|  | **PATIENT/CAREGIVER ACTIVATION** | | | |  |  |  |  |  |  |  |
|  | NR (physicians' compliance-gaining strategies) | Smith | | 2004 | n/a | Patient/caregiver activation: Language used by physicians to influence patents; definition provided. | Patients and providers (not further specified) | 37 patients & 18 providers; primary health care. | Provider | 57 items, 10 behavior categories (see Supplementary Table 1), 2 domains (Directness & Completeness). | Frequency |
|  | St Andrews Behavioural Interaction Coding Scheme (SABICS) | Zhou | | 2012 | n/a | Patient/caregiver activation: Nurse-child interaction in terms of encouraging cooperative child behaviour; definition provided. | Children aged 3-5 in a community-based dental context; dental nurses | Range, 36-55 patients & 6-19 providers; 2 different samples; dentistry. | Provider, patient | 48 items, 3 domains: Protocol behaviors, Nurse behaviour & Child behaviour. | Frequency, duration. |
|  | NR (Patient active involvement in mental health interaction) | Bonfils | | 2017 | n/a | Patient/caregiver activation: Patient involvement; definition provided. | Patients with severe/chronic mental health issues and providers in mental healthcare | 166 patients & 4 providers; mental health. | Patient | 8 items | Frequency |
|  | Active Patient Participation Coding (APPC) scale | Street | | 2005 | n/a | Patient/caregiver activation: Patient participation (patients) & partnertship building & supportive talk (providers). Definitions provided. | NR | 9 patients & 9 providers; medical setting NR. | Provider, patient | 5 items, 2 subscales: Patient participation & providers' patient-centered communication. | Frequency |
|  | Patient Involvement and Collaboration in Emergency care Teams (PIC-ET) tool | Dubois | | 2023 | n/a | Patient/caregiver activation: Patient participation and collaboration. Definition providd for 'patient participation'. | Emergency care teams | Patients NA & providers NR; emergency situations. | Provider | 22 items, 5 domains: Relationship, Sharing power, Information exchange, Safe and caring environment & Social circumstances. | Frequency, evaluation |
|  | NR (caregiver activation in advanced cancer care) | Dingley | | 2017 | n/a | Patient/caregiver activation: Caregiver activation during home hospice care; definition provided. | Caregivers of (cancer) patients in hospice care, hospice nurses | 60 patients and caregivers; providers NR. | Patient's caregiver | 9 items, 2 domains: *Content* & *Processes*. | Frequency |

| Instrument name (acronym) | | | First author | Year^a^ | Validation studies | Construct | Target population | Sample(s) and size, setting | Target 'actor' | Behaviors assessed: # items, domains (if applicable) | Response type(s)^b^ |
| --- | --- | --- | --- | --- | --- | --- | --- | --- | --- | --- | --- |
|  | **PATIENT-CENTERED COMMUNICATION** | | | | |  |  |  |  |  |  |
|  | ON-Code | Alby | | 2021 | n/a | Patient-centered communication: Doctors’ patient-centered communication practices, in particular when there are linguistic and cultural differences between the speakers; definition NR. | Cancer patients; oncologists | 19 patients & 3 providers; oncology. | Provider, patient, patient's companion | 15 items, 7 domains (e.g., Markers of uncertainty in doctor's talk; see Supplementary Table 1). | Evaluation |
|  | Patient-centered Behaviour Coding Instrument (PCBI) | Zandbelt | | 2005 | n/a | Patient-centered communication: Patient-centered behaviour; definition provided. | NR | 323 patients & 15 providers; internal medicine. | Provider | 19 items, 2 domains (Facilitating behaviors, Inhibiting behaviors) & Subject area of each expression also coded. | Frequency |
|  | Behavioral Health Treatment Model for patients with Medically Unexplained Symptoms (BHTM-MUS) | Grayson-Sneed | | 2018 | n/a | Patient-centered communication: Patient-centered skills and BHTM-MUS skills derived from motivational interviewing; definition NR. | Patients with MUS and comorbid depression and/or opioid misuse, and medical clinicians. | 12 simulated patients & 161 providers; medical setting NR | Provider | 33 items, 7 domains (see Supplementary Table 1). | Frequency |
|  | Evidence-Based Patient-Centered Interviewing Method | Grayson-Sneed | | 2017 | n/a | Patient-centered communication: Basic patient-centered skills, i.e. data-gathering and emotion-handling; definition NR, except behaviorally. | NR | 12 simulated patients & 127 providers; medical setting NR. | Provider | 33 items, 6 subscales: Setting the agenda, Physical story, Personal story, Emotional story, Indirect patient-centered skills, General skills. | Frequency |
|  | Resident-centered Assessment of Interactions with Staff and Engagement tool (RAISE) | Snow | | 2018 | n/a | Patient-centered communication: Person-centered care in staff-resident interactions; Definitions provided for 'Interaction', 'Emotional tone', and 'PCC best clinical practices'. | Aged Care Patients and staff | Patients NR & providers NR; aged care. | Providers | # items NR. | Frequency, evaluation |
|  | Global Behavior Scale (GBS) | Lann-Wolcott | | 2011 | n/a | Patient-centered communication: Person-centered caregiving in gerontology; definition provided. | Long-term care residents with dementia and their caregivers | 20 patients & 52 providers; Dementia care. | Provider | 11 items, no subscales. | Evaluation |
| Instrument name (acronym) | | | First author | Year^a^ | Validation studies | Construct | Target population | Sample(s) and size, setting | Target 'actor' | Behaviors assessed: # items, domains (if applicable) | Response type(s)^b^ |
|  | Person Centered Behavior Inventory (PCBI) | Lann-Wolcott | | 2011 | n/a | Patient-centered communication: Person-centered caregiving in gerontology; definition provided. | Long-term care residents with dementia; their caregivers | 20 patients & 52 providers; dementia care. | Provider | 19 items, 2 domains: Verbal categories & Nonverbal categories. | Frequency, duration |
|  | Patient-Centered Communication Coding System (PCCCS) | Ledoux | | 2013 | n/a | Patient-centered communication; definition provided. | Children/parents dealing with pediatric obesity; their providers/health advisors | 30 children and parents & 5 providers; childhood obesity. | Provider | 16 items, 3 domains: Positive PCC, Negative PCC & Overall communication style. | Frequency, evaluation |
|  | **RELATIONSHIP CENTERED COMMUNICATION** | | | | |  |  |  |  |  |  |
|  | Caring Behavior Coding Scheme (CBCS) | Martensson | | 2021 | n/a | Relationship centered communication: Patient and provider caring and non caring behavior; definition provided. | All patients and providers | Patients NR & providers NR; nursing. | Provider | # items NR; Example items: Maintaining belief, knowing, being with, doing for, enabling. | Frequency, duration |
|  | Health Professional-Geriatric Patient Interaction Behavior Rating Code | Adelson | | 1982 | n/a | Relationship centered communication: Relational style of patients and providers; definition NR. | Geriatric patients; healthcare professionals | Patients NR & 36 providers; geriatric nursing. | Provider | # items NR. | Frequency |
|  | Vr-COPE | McDermott | | 2021 | n/a | Relationship centered communication: Relationship centered communication; definition provided. | Veterinary surgeon-client communication | 55 clients & providers NR; veterinary care. | Provider | 10 items. | Evaluation |
|  | Process of Interactional Sensitivity Coding in Healthcare (PISCH) | Sabee | | 2015 | n/a | Relationship centered communication: Interactional Sensitivity; definition provided. | Patients with type 2 diabetes /patients in general; physicians treating these patients. | 52 patients & 52 providers; various medical settings. | Provider, patient | # items NR. | Frequency |
|  | **OTHER** |  | |  |  |  |  |  |  |  |  |
|  | Coding System for Staff-Patient Interactions (CSSPI) | Kolk | | 1989 | n/a | Other: Adherence to contingency management procedures; definition NR. | Pediatric psychiatric patients and staff | 24 patients & 31 providers; mental health. | Provider, Patient | 11 items, 3 subscales: Staff antecedent, Patient behavior, Staff consequence. | Frequency, evaluation |
|  | State Space Grids | Gainforth | | 2019 | n/a | Other: Behavioral support in provider patient interacton; definition provided. | Clients in smoking cessation behavioural support interactions; behavioral support practitioners | 6 patients & 2 providers; behavior change. | Provider, patient | # items NR, 2 domains: Practitioner verbal behaviour & Client statements. | Frequency, duration. |
| Instrument name (acronym) | | | First author | Year^a^ | Validation studies | Construct | Target population | Sample(s) and size, setting | Target 'actor' | Behaviors assessed: # items, domains (if applicable) | Response type(s)^b^ |
|  | Moffitt Accrual Analysis System (MAAS) | Albrecht | | 1999 | n/a | Other: Clinical trial accrual interactions; definition provided. | Cancer patients eligible for a clinical trial, oncologists | 48 patients & 12 providers; medical oncology. | Provider | # items NR. Global judgements, MD responsiveness to PT concerns, MD directed toward signed consent, Conformity to legal consent form, Sharing of floor time, Amount info given, MD information orientation, opinion-based MD information orientation. | Frequency, evaluation |
|  | MEDICODE | Richard | | 2006 | n/a | Other: Content of medication discussion; definition provided. | Patients visiting a GP, GPs | 462 patients & 40 physicians; general practice. | Provider, patient | 40 items, 4 domains: General information, Knowledge of the drug, Discussion of prescription, Effects of the drug. | Frequency |
|  | NR (Planning talk within motivational interviewing) | Copeland | | 2017 | n/a | Other: Discussion of plans and goals; Definitions provided for 'Plans' and 'Goals'. | Clients and counselors engaging in MI for behavior change. | 50 clients & providers NR; weight loss. | NR | # items NR, 2 domains (Goals & Plans); Goals: 2 sub-domains (Specificity, Commitment), Plans: 3 sub-domains (Past, Continuing/hypothetical, Future) & 2 criteria (Specificity, Commitment). | Frequency, evaluation |
|  | Refined Cue Utilization and Engagement in Dementia (CUeD) | Liu | | 2020 | n/a | Other: Dyadic behaviors during mealtime interactions; definition provided for 'intake episode'. | Dementia patients living in a nursing home; nursing home staff | 25 patients & 29 providers; dementia care. | Provider, patient | # items NR, 4 domains: Resident intake process, Caregiver & resident verbal behaviors, Caregiver nonverbal behaviors & Resident nonverbal behaviors. | Frequency, duration |
|  | NR (goal communication in palliative care decision-making consultations) | Gramling | | 2015 | n/a | Other: Explicit verbal goal expression in palliative care consultations; Definition provided for 'goals'. | NR | 72 patients and their families & providers NR. End-of-life care. | Provider, patient | 11 items, 3 domains: Patient goal expression, Provider goal expression & Other aspects of conversation . | Frequency |
| Instrument name (acronym) | | | First author | Year^a^ | Validation studies | Construct | Target population | Sample(s) and size, setting | Target 'actor' | Behaviors assessed: # items, domains (if applicable) | Response type(s)^b^ |
|  | NR (workflow task coding scheme) | Asan | | 2015 | n/a | Other: Interaction styles with EHRs; definition provided. | Primary Care Patients; physicians | 18 patients & 6 providers; primary care. | Provider | # items NR. | Frequency, duration |
|  | Problem, Analysis or Assessment, and Solution (Pas® System) | Van Mil | | 1997 | n/a | Other: Patient questions about medication and pharmacist assessment of question and solution offered; definition NR. | Patients asking questions to pharmacists about medication; pharmacists responding to these questions | NA | Provider, patient | 15 items, 3 domains: Patient questions, Provider analysis/assessment of the nature of the question & Provider solution offered. | Frequency |
|  | eSEGUE | Assis-Hassid | | 2015 | n/a | Other: Patient-doctor-computer communication; definition provided. | Patients and providers in primary care | NA | Provider | 23 items, 5 dimensions: Set the stage, Elicit information, Give information, Understand the patient’s perspective, End the encounter. | NR |
|  | NR (safety-netting communication behaviors in primary care encounters) | Edwards | | 2019 | n/a | Other: Safety-netting communication behaviors; definition provided. | Patients in primary care; primary care providers | 32 patients & 19 providers; general practice. | Provider, patient | # items NR, 4 domains: Administrative codes, Safety netting contextual codes, Safety-netting advice codes & Problem contextual codes (optional). | Frequency |
|  | NR (coding of patient-provider self-care communication and EMR usage) | Arar | | 2006 | n/a | Other: Self-care management practices; definition NR. EMR usage; definition provided. | NR | 50 patients & 6 providers; chronic illness /primary care. | Provider, patient | # items NR. | NR |
|  | Analysis System for Self-Efficacy Training (ASSET) | Zinken | | 2008 | n/a | Other: Self-efficacy enhancement techniques; Definition provided. | Patients with Type 1 or 2 diabetes, and health professionals applying self-management interventions. | 12 patients & 4 providers; type 1 diabetes. | Provider | 13 items, 6 domains; Self-efficacy driven verbal techniques & and patient's response to those techniques. | Frequency, duration |

| Instrument name (acronym) | | | First author | Year^a^ | Validation studies | Construct | Target population | Sample(s) and size, setting | Target 'actor' | Behaviors assessed: # items, domains (if applicable) | Response type(s)^b^ |
| --- | --- | --- | --- | --- | --- | --- | --- | --- | --- | --- | --- |
|  | NR (transparency in patient-provider communication) | Robins | | 2011 | n/a | Other: Transparency in patient-provider communication; definition provided. | Primary care patients and physicians | 259 patients & 33 providers; primary care. | Provider | 9 items, 2 domains: Process & Medical content. | Frequency |
|  | NR (metaphor use) | Macagno | | 2019 | n/a | Other: Use and problematic understanding of metaphors; Definitions provided for 'Metaphors' and 'Problematic understanding'. | Patients; health care providers (not further specified) | 13 patients & 6 providers; diabetes. | Provider, patient | # items NR. | Frequency |
|  | Behavioral Test of Interpersonal Skills (BTIS) | Olson | | 1991 | n/a | Other: Active listening; definition provided | NR | Patients NR & 41 providers; medical setting NR. | Provider, patient. | 3 subscales: Feeling, Content , 'Don't feel'. | Frequency |
|  | Paediatric Dental Triadic Interaction Coding Scheme (PaeD-TrICS) | Yuan | | 2019 | n/a | Other: Verbal and non-verbal interaction within a triad of dental professional-child -parent; definition NR. | Paediatric dental primare care patients and their caregivers; dental health professionals | 6 standardized patients & 5 providers; dentistry. | Provider, patient, patient's parent/carer | 45 items; 2 domains: Verbal dental professional/parent behaviors & Non-verbal behaviors. Child behaviors. | Frequency, duration. |
|  | Perioperative Child–Adult Medical Procedure Interaction Scale (P-CAMPIS) | Caldwell-Andrews | | 2005 | n/a | Other: Verbal and nonverbal interactions between children, parents, and medical personnel in the perioperative setting; definition NR. | Children undergoing anesthesia; providers working in the perioperative environment | 45 patients and parents & providers NR; pediatric surgery. | Provider, patient, patient's parent | 40 items, 4 domains: Adult-to-adult communication, Adult-child communication, Either, Child vocalizations & Nonverbal behavior. | Frequency |

**Notes:**

^a^ Year published for initial development/validation paper; ^b^ Frequency, evaluation and/or duration - whereby frequency can refer to presence/absence, amount of occurrences, timing, and/or sequence of behaviors; evaluation can refer to the extent and/or quality of an observed behavior; and duration to the length of time during which a specific behavior occurs**; ^c^** n/a, not applicable; NR, Not Reported; #, number; ^d^ Definitions –if provided– are displayed in Appendix B/ Supplementary Table 1.
