# Supplementary material for: Systematic review of observational coding systems to assess patient-healthcare professional interactions
Source: Patient Educ Couns. Author manuscript; Available in PMC 2026 Aug 16. (PMC13477582; doi:10.1016/j.pec.2025.108718)
Supplement: Supplementary file 4 [file NIHMS2172109-supplement-4.docx]

Appendix D / Supplementary Table 2a. Information about coders (number, training, procedure) who applied the coding schemes, and assessment of intra- and inter-rater reliability conducted

| Instrument name (acronym) | First author | Year | Match intended-included sample | Coders a) N raters; b) training details; c) rating methods (independent, in consensus, other) | Intra-rater reliability Type of statistic (P-value) (N) | Inter-rater reliability Type of statistic (P-value) (N) |
| --- | --- | --- | --- | --- | --- | --- |
| 5 As for Physical Activity | Carroll | 2011 | Subset | a) 2; b) NR; c) independent | NR | Cohen's kappa=1.0 (ask), .62 (assess), .81 (advise), .72 (assist), 1.0 (arrange) |
| 5a-DOC | Lawson | 2009 | Subset | a) 2; b-c) NR | NR | Range Cohen's kappa, .58-1.0 for 4/5 of the behaviors |
| Active Patient Participation Coding (APPC) scale | Street | 2005 | Unclear | a) 2; b) trained (not specified); c) independent | NR | Range Cohen’s kappa, .80-.93 |
| Advance Care Planning Communication Assessment Tool (ACP-CAT) | Yuen | 2020 | Subset | a) 6; b) 2 hour training including instruction and practice using video-recorded encounters; c) pairs of raters compared, discussed and resolved discrepancies | NR | Overall ICC=.82 (pre-training) and .83 (post-training); range ICC for individual items, .67-.97, except for item 19 (ICC=.07) |
| Analysis System for Self-Efficacy Training (ASSET) | Zinken | 2008 | Yes | a) 4; b) comprehensive manual handed out in advance of training. Coders received a short version of coding tool during 3 h training; c) coded using linguistic annotation software program ELAN | NR | Cohen’s kappa=.71 for super-categories (e.g., self-efficacy strategy) and .70 for sub-categories (e.g., verbal behavioral technique), percentage agreement=82.4% (N, NR; 4 raters) |
| Assessment of Brief Behavioural Change Counselling’ tool (ABC tool) | Fouche | 2020 | Unclear | a) 6; b) all received training in BBCC, no training on use of the tool; c) all interactions were coded by all 6 coders | Range r_P_, .478-.860 for the 5 steps and r_P_=.899 for overall scores (*p*<.001) (N=33) | ICC=.714 (first scoring) and .813 (second scoring) (N=197) |
| Behavioral Health Treatment Model for patients with Medically Unexplained Symptoms (BHTM-MUS) | Grayson-Sneed | 2018 | Unclear | a) 2; b) undergraduate students received 30 h of training; c) independent, followed by consensus | NR | Total scale: Cohen’s kappa=.87, percentage agreement, 95.7%; Educating and Informing: kappa=.76, Motivating: kappa=.84, Treatment Statements: kappa=.86, Commitment and Goals: kappa=.83, Negotiates Plan: kappa=.83, Patient-Centered Skills: kappa=.97, Patient- Centered Emotional Skills: kappa=.90, range percentage agreement over subscales, 85-100% (N=33) |
| Behavioral Test of Interpersonal Skills (BTIS) | Olson | 1991 | No | a) 2; b-c) NR | Nurses' behavior: range kappa, .85-1.0; Patients' behavior: range kappa, .85-.90 (NOTE: unclear if this refers to intra- or interrater reliability) | Nurses' behavior: range Kappa, .85-1.0; Patients' behavior: range Kappa, .85-.90 (NOTE: unclear if this refers to intra- or interrater reliability) |
| Behaviour Change Counselling Index (BECCI) | Lane | 2005 | Yes | a) 3; b) trained in BCC; c) 1 coder rated data sets 1-3, 2 different coders independently rated set 4 (unclear who rated data sets 5-9). | Rater 1: ICC=.66 (smoking cessation) and .90 (diabetes), rater 2: ICC=.60 (smoking cessation) and .87 (diabetes) (N=24 per setting, 1 rater) | ICC=.79 (smoking cessation) and .93 (diabetes consultations) (N=24 per setting) |
| Brief Decision Support Analysis Tool (DSAT-10) | Stacey | 2008 | Yes | a) 5; b) trained in applying original DSAT instrument, including completing online decision support tutorial; raters assessed encounters and sought consensus until they reached 85% agreement for three consecutive ratings; c) independent | NR | Total sample, kappa=.550 (95% CI, .490-.610); consultations of trained nurses, kappa=0=.617 (95% CI, .434-.800), ICC=.759; consultations of untrained nurses, kappa=.489 (95% CI, .415-.564), ICC=.587 |
| Cancode interaction analysis system (Cancode) | Dent | 2005 | No | a) 2 available but unclear if used in current study; b) NR; c) coder 1 recoded 10 randomly selected consultations 3 months after initial coding for intra-rater reliability, coder 2 coded 10 randomly selected consultations for inter-rater reliability. | Weighted kappa for audio/video=.86/.79 (Content) and .80/.58 (Function) (N=10 per mode, 1 rater) | Weighted kappa for audio=.59 (Content) and .50 (Function) (N=10) |
| Instrument name (acronym) | **First author** | **Year** | **Match intended-included sample** | **Coders a) N raters; b) training details; c) rating methods (independent, in consensus, other)** | **Intra-rater reliability Type of statistic (P-value) (N)** | **Inter-rater reliability Type of statistic (P-value) (N)** |
| Caring Behavior Coding Scheme (CBCS) | Martensson | 2021 | Unclear | a) 2; b) coders trained in use of coding system and software, until coders obtained Kappa of .80 or above; c) independent | NR | Cohen’s kappa=.82 (range, .77–.88) (N=7) |
| Chronic Pain Coding System (CPCS) | Henry | 2016 | Unclear | a) 2; b) NR; c) independent | NR | ICC=.72 (pain utterances), range Cohen's kappa (all subcategories), 0.0-.88 and (major categories) .5–.7 |
| Coding System for Staff-Patient Interactions (CSSPI) | Kolko | 1989 | Yes | a) 2; b) 10 h didactic training and practice coding sample videos, 8 weekly meetings w/ 2 senior authors, 5 h supervised training until both had achieved 80% reliability compared to criterion tapes; c) NR | NR | Patient behavior: range percentage agreement, 64-86%, range r_P_, .67-.94 (*p*<.001); Staff consequence: range percentage agreement, 71-100%, range r_P_, .60-1.0 (*p*'s<.01) (N=18 observation intervals); Correctness of staff consequences: range percentage agreement, 80-94%, range r_P_, .68-.92, Incorrectness of staff consequences: range percentage agreement, 67-91%, range r_P_, .70-.86 (6 staff behaviors) |
| Content coding for contextualization of care (4C) | Weiner | 2014 | Unclear | a) 2; b) coders involved in development of coding scheme; c) independent | NR | Cohen's kappa=.76 (provider probes) (*p*<.001); percentage agreement=94% (contextual factor identified/revealed), 85% (care plan contextualized) (*p*'s<.001) |
| Davis Observation Code (DOC) | Callahan | 1991 | Yes | a) 2; b) NR; c) independent | NR | Percentage agreement=82.7% (N=32) |
| Decision Analysis System for Oncology (DAS-O) | Brown | 2011 | Yes | a) 2; b) coders trained on five transcripts from another study by experienced coder, until high level of agreement was established; c) independent | Mean Cohen's kappa=.648, 5 subscales: range, .493-.744 (Cohen's kappa) and .596-.950 (percentage agreement) (N=5 transcripts in Australia-New Zealand sample and N=12 transcripts in Swi-Ger-Aus sample) | Mean Cohen's Kappa=.584; 5 subscales: range, .486-.644 (Cohen's kappa) and .695-.855 (percentage agreement) (N=6 transcripts in Australia-New Zealand sample and N=12 transcripts in Swi-Ger-Aus sample) |
| Decision Support Analysis Tool (DSAT) | Butow | 2010 | Yes | a) 3; b) trained on 5 transcripts until high level of agreement with independent highly trained coder was established; c) independent | NR | NR |
| Decision Support Analysis Tool (DSAT) | Guimond | 2003 | Yes | a) 8; b) 3 h training followed by practice with training transcript, follow-up session to discuss issues/questions, final training consisting of transcript coding that had to meet pre-determined 80% agreement with expert panel coding; c) independent | NR | Decision support: percentage agreement, 75%, Cohen's kappa=.58; Communication skills: percentage agreement, 76%, kappa=.68 (N=34) |
| Dementia Competent Social Interaction (DSI) and Culturally Competent Social Interaction (CSI) Coding Schemas | Kim | 2012 | Yes | a) 2; b) 7 h training program, social interaction behavioral code definitions were clarified w/ examples, practiced until interrater reliability with PI was Cohen’s kappa≥.80; c) NR | NR | Range Cohen’s kappa, .73-.83 (Dementia competent social interaction, DSI) and .69-.82 (Culturally competent social interaction, CSI), range percentage agreement, 75-84.62% (DSI) and 76.92-84.21% (CSI) |
| Detail of essential elements and participants in shared decision making (DEEP-SDM) | Clayman | 2012 | Subset | a) 2; b) NR; c) independent, followed by consensus | NR | NR |

| Instrument name (acronym) | First author | Year | Match intended-included sample | Coders a) N raters; b) training details; c) rating methods (independent, in consensus, other) | Intra-rater reliability Type of statistic (P-value) (N) | Inter-rater reliability Type of statistic (P-value) (N) |
| --- | --- | --- | --- | --- | --- | --- |
| Elements of Informed Decision Making (IDM) | Braddock | 1999 | Yes | a) 4; b) trained over several weeks through detailed definitions of elements, joint coding and discussion, and independent coding of pilot study audiotapes; c) independent | Decision complexity, range Cohen's kappa, .53-.66 (N, 5% of 1057 audiotapes, 4 raters) | Overall agreement on presence of decision, 73%, Cohen's kappa=.44; kappa for decision complexity=.58; range kappa for presence/absence of elements, .19-.61 (N, 10% of N=1057) |
| Empathic and Potential Empathic opportunity (E-PE-O) method | Eide | 2004 | Unclear | a) 2; b) coders first analyzed together a consultation to achieve consensus in scoring; c) both coders coded all consultations | NR | Total percentage agreement, 56% (272 EO + PEO) |
| Empathic Communication Coding System (ECCS) | Bylund | 2002 | Unclear | a) 1 for empathic opportunity, 2 for physician responses; b) coders watched 10 encounters to identify empathic opportunities and achieve consensus; c) independent, followed by consensus | NR | Percentage agreement, 80% (occurrence of empathic opportunity) (N=10), Cohen’s kappa=.73 (responses to N=249 empathic opportunities) |
| eSEGUE | Assis-Hassid | 2015 | n/a | n/a | NR | NR |
| Evidence-Based Patient-Centered Interviewing Method | Grayson-Sneed | 2017 | Unclear | a) 2; b) undergraduate students received 30 h of training; c) independent, followed by consensus | NR | Total scale: Cohen's kappa=.90, percentage agreement, 97.5%; Personal Story items: Percentage agreement, 99.3%; Agenda Setting: kappa=.94, Physical Story and Indirect Patient-Centered Skills: kappa=1.0, Emotional Story: kappa=.86, General Patient-Centered Skills: kappa=.86 (N=25) |
| Four Habits Coding Scheme (4-HCS) | Catani | 2018 | Yes | a) 3; b-c) NR | NR | NR |
| Four Habits Coding Scheme (4-HCS) | Jensen | 2010 | Yes | a) 4; b) 3 coders completed 12 h coding course with developer of 4HCS and 3 Four Habits Model teachers, consisting of watching, coding, and discussing encounters, based on 4HCS codebook. Raters completed a second similar 6 h training session 10 weeks later. Fourth coder trained in 1 session; c) independent | NR | Total score r_P_ and ICC>.70 for all pairs of raters. Across items within single videos, r_P_ was never below .60 after the first 50 videos. At item and habit level r_P_ remained unsatisfactory for some rater pairs mostly due to low variance on some items. |
| Four Habits Coding Scheme (4-HCS) | Krupat | 2006 | Yes | a) 2); b) raters trained using randomly selected tapes, it took 8-10 h training per coder to reach acceptable levels of reliability; c) independent | NR | r_P_=.70 (Habit 1), .80 (Habit 2), .71 (Habit 3), .69 (Habit 4), .72 (overall) (N=13) |
| Four Habits Coding Scheme (4-HCS) / Physician–patient relationship competence assessment scale | Bellier | 2020 | No | a) 4; b) training consisted of independently coding 5 consultations and discussing the ratings; c) independent | Absolute-agreement ICCs (scores from all raters match exactly). Range average ICC across subscales, .76-.9, overall average ICC, .91; For 2 most experienced raters only: individual ICC=.89 | Absolute-agreement ICCs (scores from all raters match exactly). Range average ICC across subscales, .68-.84 (three out of four habits fulfilled criterion of .70), overall average ICC, .82; For 2 most experienced raters only: individual ICC=.83 |
| Global Behavior Scale (GBS) | Lann-Wolcott | 2011 | Yes | a) 2; b-c) NR | NR | NR |
| Health Coaching Index (HCI) | Sohl | 2021 | Subset | a) 3; b) 4-6 independent raters scored 6 coaching sessions, and discussed thier codes; c) independent. | NR | Total score, ICC=.81; subscales, range ICC, .48-.86 |
| Health Professional-Geriatric Patient Interaction Behavior Rating Code | Adelson | 1982 | Yes | a) 5; b) observers read the code, studied definitions and examples of target behaviors, and discussed questions with the trainer. Observers rated 4 interactions simultaneously with the trainer and discussed discrepancies, and achieved an average agreement rate with the trainer of 82% across categories; c) NR | NR | Perfect agreement across all categories > 85% (N=22) |
| Indicators of Health Behavior Change (IHBC) | Potempa | 2023 | Unclear | a) 2; b) NR; c) 10% double coding | NR | Overall: ICC=.89, across constructs: range ICC, .876-.922 (N, 10% of N=59) |
| Instrument name (acronym) | **First author** | **Year** | **Match intended-included sample** | **Coders a) N raters; b) training details; c) rating methods (independent, in consensus, other)** | **Intra-rater reliability Type of statistic (P-value) (N)** | **Inter-rater reliability Type of statistic (P-value) (N)** |
| Informed Decision Making Measure (IDM) | Leader | 2012 | NR | a) 4 (2 per audio-recording); b) coders received an IDM coding manual and had extensive discussions and training with one of the investigators. Coders practiced with preexisting training audio file set, until they achieved at least 80% accuracy in identifying presence/absence of each IDM element in 50 different training audiotapes; c) independent | NR | Total score: mean ICC across rater pairs=.81 (95% CI, .75-.86 with normal approximation, .67-.88 with bootstrap), range ICC across pairs, .68-.94; individual items: range Cohen's kappa, .31-.86 (N=132) |
| Informed Decision Making Measure (IDM) | Weiss | 2008 | Yes | a) 1; b) trained expert in IDM (not further specified); c) each consultation rated once | NR | NR |
| Kin interaction coding system (KINcode) | Laidsaar-Powell | 2015 | Yes | a) 2; b) NR; c) single coding, except for random 20% of transcript which was independently double-coded | Individual items: range Cohen's kappa, .44-1.0, M_kappa_=.75 (4 role items), M_kappa_=.82 (all other items) (N, 20% of N=72) | Range Cohen's kappa, .46-1.0 (all items), M_kappa_=.74 (4 role items), .81 (all other items) (N, 20% of N=72 consultations) |
| Liverpool-MAAS (LIV-MAAS) | Enzer | 2003 | Yes | a) 5 (test sample), 3 (final sample); b) collaborative training, working through and discussing all items, followed by one-on-one tutorial with investigator, 5 h total; c) independent | NR | Range ICC, .69-.91 (test sample), .71 (2 raters)-.78 (3 raters) (final sample) |
| Manchester Observation Code (MOC) for genetic counseling | Liede | 2000 | Yes | a) 1, and 10 for 3 out of 21 videos; b) 10 minutes training; c) NR | Cohen's kappa=.79 (N=3, 1 rater) | Overall: Cohen's kappa=.70; 4 domains: range kappa, .70-.82 (N=3, 10 raters) |
| Mappin'SDM | Kasper | 2012 | Unclear | a) 3; b) NR; c) rating in random order, all videos double-coded. | NR | Mean r_P_=.74 (dyad), .81 (patient) and .87 (doctor) |
| Mappin'SDM (Norwegian) | Kienlin | 2017 | Yes | a) 2; b) ompleted curriculum-based coder training; c) independent | NR | Averages over the 11 items in each of the 3 scales: mean T=.77, range T, .57-.93 (MAPPINdoctor); mean T=.82, range T, .61-1.0 (MAPPINpatient); mean T=.77, range T, .61-.96 (MAPPINdyad); range percentage agreement over items, 66%-100% (mean, MAPPINdoctor: 79%, patient: 86%, dyad: 82%) |
| Measure of Patient-Centered Communication (MPCC) | Shields | 2005 | Unclear | a) 3; b) NR; c) computer coding. The coding by humans was only for the purpose of validating the automated computer coding. | NR | Spearman Brown's prophecy formula=.79 (overall), .67 (component 1), .89 (component 2), .43 (component 3) (N, NR) |
| Medical Interaction Process System (MIPS) | Ford | 2000 | Yes | a) 3; b) previously trained in interaction coding (not specified). Pilot coding of 2-3 videotapes together and 1 videotape independently, all codes discussed in various rounds; c) independent | NR | ICC Sample 1: range, .91-1.0 (patient & clinician content codes), .62-.99 (patient & clinician modes), .91-.96 (patient & clinician affective global categories) (N=20); Sample 2: range, .54-1.0 (patient & clinician content codes), .47-1.0 (patient & clinician modes), .68-.86 (patient & clinician affective global categories), .54-.72 (patient & clinician kinesic global categories) (N=6) |
| Medical Interaction Process System (MIPS) | Ford | 2004 | Yes | NR | NR | NR |
| MEDICODE | Richard | 2006 | Yes | a) 4; b) 2-4 weeks training; c) independent | Range percentage agreement, 87-100%, range Cohen's kappa, .01-1.0 (N, 7.6% of N=52) | Range percentage agreement, 75-100%, range Cohen's kappa, .01-.79 (N, 12.3% of N=422) |
| Moffitt Accrual Analysis System (MAAS) | Albrecht | 1999 | Subset | a) 4; b-c) NR | NR | Mean Cohen’s kappa for the 73 checklist items=.67 (SD=0.16; range, .30-0.0), mean kappa across the 17 global judgment items=.64 (SD= 0.11; range, .53-.82) (N, 15% of N=48) |
| Instrument name (acronym) | **First author** | **Year** | **Match intended-included sample** | **Coders a) N raters; b) training details; c) rating methods (independent, in consensus, other)** | **Intra-rater reliability Type of statistic (P-value) (N)** | **Inter-rater reliability Type of statistic (P-value) (N)** |
| Motivational Interviewing Skills Code - modified (MISC 2.1) | Lord | 2015 | Yes | a) 3; b) hour-long meetings and coding homework spread over several weeks, using 11 session recordings; c) independent | 3 methods used to compute reliability: Agreement by 1. Words, 2. Matching whole utterances, 3. Matching split utterances. range Cohen's kappa Method 1, .731-.829, multi-kappa=.774; Method 2, .801-.865, multi-kappa=.831, Method 3: .762-.820, multi-kappa=.786 (N=26, 3 raters) | 3 methods were used to compute reliability: Agreement by 1. Words, 2. Matching whole utterances, 3. Matching split utterances. Range Cohen's kappa Method 1, .687-.715, multi-kappa=.700; Method 2, .760-0.783, multi-kappa=.771, Method 3, .705-.722, multi-kappa=.714 (N=31, 3 raters) |
| Motivational Interviewing Skills Code (MISC) | Moyers | 2003 | Yes | a) 2; b) upper-level psych. grad. students w/ 40 h of training coding tapes simultaneously with expert, then independently and double-coded by expert, and finally independently with random sample double-coded by expert; c) independent | NR | ICC global assessments: range, .3895-.7912 (provider), .2524-.7583 (patient), .5829-.6315 (interaction); ICC behaviors: range, -.0441-1.0 (provider), .5314-0.8653 (patient) (N=50) |
| Motivational Interviewing Treatment Integrity (MITI) rating system | McMaster | 2015 | Unclear | a) 3; b) extensively trained in motivational interviewing; coders were sent audio samples directly and allowed to proceed with coding as they chose; c) independent | NR | Range Fleiss's kappa, –.708-.63, Md_kappa_=.284 (across all 3 raters); Raters 1 vs. 2: range Cohen's kappa, –0.94-.371, Md_kappa_= –.164; Raters 1 vs. 3: range Cohen's kappa, –.262-.885, Md_kappa_=.524; Rater 2 vs. 3: range Cohen's kappa, –.458-.422, Md_kappa_= –.074; Behavior counts: kappa=.89 (open questions), .82 (closed questions), .89 (associated reflection to question ratio) |
| Motivational Interviewing Treatment Integrity Code (MITI) rating system (Swedish) | Forsberg | 2007 | Yes | a) 8; b) five 6 h training days, then 3 h sessions and booster sessions; c) first 20 min of each session blindly coded by 2 coders in variable pairs | NR | Across 2 reliability tests: Cronbach's alpha=.89/.96; ICC=.61/.81; range ICC for subscales, .42-.79 |
| Motivational Interviewing Treatment Integrity Code (MITI) rating system (Swedish) | Forsberg | 2008 | Yes | a) 7; b) NR; c) first 20 min of each session were blindly coded by 2 coders in variable pairs | NR | NR |
| Multi-Dimensional Analysis of Patient Outcome Predictions (MD.POP) | Ménard | 2018 | Unclear | a) 2; b) trained using 4 encounters; c) independent | NR | Cohen's kappa=.83 (identification of speech segments as POP or not), range Cohen's kappa, .92-.94 (coding the POP dimensions of each POP identified) (N=20) |
| Nonverbal Accommodation Analysis System (NAAS) | D'Agostino | 2011 | Subset | a) 2; b) NR; c) 2 raters coded 10 randomly selected consultations, 1 rater coded the rest | range r_P_, .82-1.0 across the 10 NAAS behaviors (*p*<.01) (10 consultations) | Range r_P_, .81-.96 (*p*<.01) |
| Nonverbal communication in doctor–elderly patient transactions (NDEPT) | Gorawara-Bhat | 2007 | Yes (Patients), Unclear (Providers) | a) 2; b) 40 h of training by senior coder, distributed over 3-weeks; c) independent | NR | Cohen's Kappa=.957 (static attributes), .871 (dynamic attributes), .402 (kinesic attributes) (N=50) |
| Instrument name (acronym) | **First author** | **Year** | **Match intended-included sample** | **Coders a) N raters; b) training details; c) rating methods (independent, in consensus, other)** | **Intra-rater reliability Type of statistic (P-value) (N)** | **Inter-rater reliability Type of statistic (P-value) (N)** |
| NR ("Strength of connectional silence" taxonomy) | Bartels | 2016 | Yes | a) 3; b) NR; c) in consensus | NR | Cohen’s kappa=.64 (N=67 silences) |
| NR (ambivalence and reluctance in palliative care ) | Gupta | 2023 | Yes | a) 5; b) 4-week training c) in consensus | NR | ICC=.88 across all codes |
| NR (Automatic annotation of information-giving in clinical conversations) | Mayfield | 2014 | Yes | n/a | NR | Cohen's kappa of automatic versus manual annotation=.573 (N=40) |
| NR (behavior change therapy delivery and receipt) | Hoekstra | 2022 | Subset | a) 2; b) NR; c) independent. After each 2-3 transcripts, reliability measures were calculated and disagreements resolved. Minor changes to coding scheme were made if needed. | NR | Bias-adjusted kappa (PABAK)=.98 (counselor statements), .98 (client statements) |
| NR (Caregiver Activation in Advanced Cancer Care) | Dingley | 2017 | Yes | a) 3; b) NR; c) independent coding of 15% of the total recordings | NR | NR |
| NR (Characterize client responses to practitioner-delivered behaviour change techniques) | Gainforth | 2016 | Yes | a) 2; b) researchers with prior experience of coding interactions and involved in development of present scheme; c) independent (presumed) in 3 waves (5 transcripts/wave) with inter-rater reliability calculated after each wave and followed by discussion and adaptations to coding framework | NR | Percentage agreement and PABAK (prevalence and bias-adjusted kappa) statistic across waves: 75.1% and .77 respectively (N=15)  *Note:* PABAK adjusts for (1) coders sharing bias in use of categories and (2) high prevalence of negative agreement |
| NR (coding frame for shared decision-making) | Singh | 2010 | Yes | a) 2; b) NR; c) single coding | Full scale, percentage agreement=95% (N, 20% of N=63, 1 rater) | Full scale, percentage agreement, 90% (20% of N=63) |
| NR (coding of Patient-provider self-care communication and EMR usage) | Arar | 2006 | Unclear | a) 2; b) NR; c) coded by 1 coder. All transcribed videotapes checked for accuracy by a second trained research assistant. | Cohen's kappa=.90 (N=5, 2 raters) | Cohen's kappa=.65 (self-care categories) (N=10) |
| NR (coding scheme to identify the content of general practitioner communication) | Di Caccavo | 2000 | Yes | a) 2; b) NR; c) independent | NR | Kappa=.72, range, .62-.90 (N=12) |
| NR (Cognitive strategies used by General Practitioners in diagnostic reasoning) | Donner-Banzhoff | 2017 | Yes | a) 2; b) NR; c) Independent, selection of transcribed material discussed within the full team. | NR | Percentage agreement, 84% (N=3 consultations, N=3 interviews with general practitioners) |
| NR (Complex oral information transfer) | Nordfalk | 2019 | Unclear | a) 3; b) NR; c) independent, followed by group discussion to resolve disagreements; process repeated 4 times; next 10% of statements independently coded to calculate reliability. | NR | ICC=.761 (COPIN), .723 (PROPIN) |

| Instrument name (acronym) | First author | Year | Match intended-included sample | Coders a) N raters; b) training details; c) rating methods (independent, in consensus, other) | Intra-rater reliability Type of statistic (P-value) (N) | Inter-rater reliability Type of statistic (P-value) (N) |
| --- | --- | --- | --- | --- | --- | --- |
| NR (Decision-making around limiting life supporting treatment in neonatal intensive care) | Shaw | 2023 | Yes | a) 2; b) CA experts; c) first agreement was sought on what counted as decision point, then independent coding | NR | Range Cohen's Kappa, .24-.81 |
| NR (Global Consultation Rating Scale (GCRS) adapted for pediatric subspecialties) | Coburn | 2020 | Yes | a) 2; b) raters used audio from original GCRS training videos to achieve reliability with each other and with master codes provided by GCRS developers. Scores were discussed, GCRS manual adapted if needed; c) 20% of tapes were double-coded | NR | Agreement r=.74 (SD=0.16), range, .44 (patient’s perspective)-1.0 (problem exploration); percentage agreement, 75.24% (SD=11.53), range, 57.10–100% |
| NR (Goal communication in palliative care decision-making consultations) | Gramling | 2015 | Unclear | a) 2; b) 30 h over a two-week period; c) independent (presumed) | NR | Cohen’s kappa=.82 (identifying goal expressions), .78 (emotion expression), .64 (prognosis expression, treatment options), .63 (existential suffering) (20% of N=72) |
| NR (Metaphor use) | Macagno | 2019 | Yes | a-b) NR; c) independent | NR | Percentage agreement, 99.1%, range, 93-100%; Krippendorff's Alpha=.89, range, .768-1.0 (35% of N=46) |
| NR (number and type of problems discussed in primary care consultations) | Procter | 2014 | Yes | a) Phase 1: 5 coders, Phase 2: 2 coders, Phase 3: 1 coder; b) No training; c) Phase 1: group discussion; phase 2: independent coding | NR | ICC=.93 (number of problems recorded), mean percentage agreement, 96.5% (ICPC disease areas), 90% (issue types) (60 consultations) |
| NR (Nurse Patient Touch) | Bottorff | 1994 | Yes | a) 3; b) raters introduced to observation schedule, coding procedures, and representative examples of videotaped data. Next, they coded videotaped segments jointly and independently, disagreements were resolved, and assisted in revising definitions if necessary. Training continued until acceptable level of agreement was reached (Cohen's kappa statistic); c) independent | Range Cohen's kappa, .31-0.99; value for verbal message with touch was poor (value NR); generalizability coefficient for duration of touch (acceptable for 2/3 coders, value NR) (N=3, 3 raters) | Cohen's kappa: range, .38-.91 (interactional context), .18-.71 (touch events); Proportion agreement: range, .69-.83 (touch events); Generalizability coefficients: range, .45-1.0 (for all categories with sufficient observations) (N=8 interactions, 3 raters) |
| NR (Patient active involvement in mental health interaction) | Bonfils | 2017 | Yes | a) 4; b) NR; c) independent, each 5th transcript coded by all | NR | Range Gwet's agreement coefficient, .46-.84 for individual items |
| NR (physicians' compliance-gaining strategies) | Smith | 2004 | Yes | a) 3; b) coders trained in use of coding system and software; c) two-phase coding procedure: first identification and consensus seeking about identification of utterances, then application of 1/57 codes to each utterance | NR | Range Cohens' kappa, .63-.65, range percentage agreement, 61.8-65.9% (N=37) |
| NR (Planning talk within motivational interviewing) | Copeland | 2017 | Yes (Patients), Unclear (Providers) | a) 10 for reliability analysis, 3 for inter-rater reliability; b) 45 min presentation with examples, followed by practice coding; c) for reliability: 1 transcript and 15 example goals/plans coded independently. For inter-rater reliability: 2 or 3 sessions independently coded by each, 15% of total data | NR | Percentage agreement, 61% |
| NR (Safety-netting communication behaviors in primary care encounters) | Edwards | 2019 | Yes | a) 2; b) joint development, same coders involved in eventual coding; c) independent for N=32 (10%), followed by consensus, next 1 rater coded rest of consultations | NR | Cohen's kappa=1.0 at consultation level (presence/absence of safety-netting advice) (N=32 consultations), .77 at problem level (presence/absence of safety-netting advice) (N=55 problems). ICC=.88 (occurrences of safety-netting advice), .73 (medical problem) |
| Instrument name (acronym) | **First author** | **Year** | **Match intended-included sample** | **Coders a) N raters; b) training details; c) rating methods (independent, in consensus, other)** | **Intra-rater reliability Type of statistic (P-value) (N)** | **Inter-rater reliability Type of statistic (P-value) (N)** |
| NR (the process and content of general practice consultations involving the prescription of antibiotic agents) | Cockburn | 1987 | Yes | a-c) NR | NR | Cohen's kappa NR, *p*<.05 for every code (20% of N NR) |
| NR (transparency in patient-provider communication) | Robins | 2011 | Yes | a) 4; b) NR; c) 1st phase: 4 researchers reviewed 105 interviews independently, followed by consensus, then 1 researcher completed remaining 158 interviews | NR | Cohen's Kappa=.84 (N=45 transparency utterances) |
| NR (Verbal interpersonal clinical problem-solving skills) | Pridham | 1980 | Unclear | a) 3; b) NR; c) independent by 2 coders, third coder reviewed codes and scored for agreement. | NR | Percentage agreement: range, 73.9-92.2%, range r_P_, .47-.90, range r_S_, .61-.90 (N=5) |
| NR (Workflow task coding scheme) | Asan | 2015 | Yes | a) 2; b) trained with practice videos until kappa≥.6; c) independent (presumed). Coders coded 1 video/week (4 in total) to check and maintain required reliability. | NR | Range reliability scores, .63-.76. Reliability scores were calculated at one-second levels: if two coders coded the start of an event in the period of X +/- 1, it was counted as an agreement. |
| Observing patient involvement in decision making (OPTION^12item^ revised) | Butow | 2010 | Yes | a) 3; b) trained on 5 transcripts until a high level of agreement with independent highly trained coder; c) independent | NR | NR |
| Observing patient involvement in decision making (OPTION^12item^ revised) | Weiss | 2008 | Yes | a) 1; b) trained expert in OPTION (not further specified); c) each consultation rated once | NR | NR |
| Observing patient involvement in decision making (OPTION^12item^ revised) (German) modified | Keller | 2013 | Yes | a) 4; b) extensive training including calibration session for each rater pair to reach consensus; c) independent | NR | Raters 1/3: ICC range, .08-.61, item-level weighted kappa range, .11-.44, total score r_P_=.69 (*p*=.002); Raters 2/4: ICC range, .03-.56, item-level weighted kappas range, -.05-.38, total score r_P_=.56 (*p*=.013) (N=20) |
| Observing patient involvement in decision making (OPTION^12item^ revised) (Italian) | Goss | 2007 | Yes | a) 2; b) based on original OPTION manual and training with 7 audiotaped consultations from the UK provided by scale developer; c) independent | Total score: rater 1, ICC=.94, rater 2: ICC=.83 (N=30) | ICC=.85, item-level range, .46-.82 (N=30 consultations) |
| Observing patient involvement in decision making (OPTION^12item^) | Elwyn | 2003 | Yes | a) 2; b-c) NR | Generalizability coefficient=.66 (N=21) | Cohen's kappa=.66, ICC=.62 (N=186) |
| Observing patient involvement (OPTION^5item^) | Barr | 2015 | Yes | a) 2; b) previously trained in applying OPTION^12item^; independently applied coding using OPTION^5item^ development paper and manual to 2 subsequent sets of 5 consultations, and discussed disagreements; c) independent | r_P_=.93 (95% Credibility interval .83- .97) (N=22, 1 rater) | Range percentage agreement, 75.5-90.1%, range Cohen's kappa, .21-.64 (items 1-5) (*p*'s<.0001); overall ICC accuracy=.66 (95% Credibility Interval, .51-.91), overall ICC consistency=.70 (95% Credibility Interval, .56-.94) (N=311) |
| Observing patient involvement (OPTION^5item^) | Elwyn | 2013 | NR | NR | NR | NR |
| Instrument name (acronym) | **First author** | **Year** | **Match intended-included sample** | **Coders a) N raters; b) training details; c) rating methods (independent, in consensus, other)** | **Intra-rater reliability Type of statistic (P-value) (N)** | **Inter-rater reliability Type of statistic (P-value) (N)** |
| Observing patient involvement (OPTION^5item^) | Stubenrouch | 2016 | Yes | a) 2; b) trained using original manuals and 7 virtual consultations from OPTION instrument website; then independently coded 5 consultations using OPTION^12item^. This was repeated until kappa≥.6); c) independent | NR | Unweighted Cohen's kappa=.76 (N=60), range unweighted Cohen's kappa, .69-.85 across settings (N=15 each) |
| Observing patient involvement (OPTION^5item^) | Vortel | 2016 | Yes | a) 2; b) NR; c) independent | NR | Krippendorf's alpha=.85 (N=27 consultations) |
| Observing patient involvement (OPTION^5item^) (German) | Kölker | 2018 | Yes | a) 2; b) 6 h training, in which raters independently assessed 5 audio recordings and 2 video recordings; results discussed and consensus reached using user manual; c) independent | Total score: ICC=.83, range item-level, ICC=.45-.86 (N=79, 1 rater) | Overall ICC=.82; range item-level ICC, .45-.77 (N=79) |
| Observing patient involvement (OPTION^5item^) (simplified, Chinese) | Chen | 2020 | Yes | a) 2; b) NR; c) independent | Total score: ICC=.945 (95%CI, .904-.965) (*p*<.001), item-level: range weighted Cohen's kappa, .469-.883 (*p*'s<.001) (N=209, 1 rater) | total score ICC=.859 (95% CI, .724-.917) (*p*<.001), range item-level weighted Cohen's kappa, .376-.649 (*p*'s<.001) (N=209) |
| Observing patient involvement in decision making (OPTION^12item^ revised (German) | Hirsch | 2012 | Yes | a) 4; b) extensive training followed by calibration sessions in pairs; c) independent | NR | raters 1-3/2-4 (sum scores): r_P_=.68/.82, mean weighted kappa=.32/.18 (N=40) |
| Observing patient involvement in decision making (OPTION^12item^ revised) | Elwyn | 2005 | Yes | a) 2; b) NR; c) independent | Total scale: rater 1, ICC=.82; rater 2, ICC=.65 (N=21) | ICC=.77 (range, .11-.98), mean Cohen's kappa=.66 (N=186) |
| Observing patient involvement in decision making (OPTION^12item^ revised) | Kasper | 2011 | Yes | a) 2; b) raters were trained by principle author of scale, on 50 consultations and included comprehensive explanation of each judgment based on manual. Raters rated videorecorded consultation again in case of disagreement; c) independent for subset of N=26 consultations | r_S_=.94 (N=15) | r_S_=.83 (N=26) |
| Observing patient involvement in decision making (OPTION^12item^ revised) | Stubenrouch | 2016 | Yes | a) 2; b) trained using original manuals and 7 virtual consultations from OPTION instrument website; then independently coded 5 consultations using OPTION^5item^. This was repeated until kappa≥0.6); c) independent | NR | Unweighted Cohen's kappa=.68 (N=60), range kappa, .63-.72 across settings (N=15 each) |
| ON-Code | Alby | 2021 | Yes | a) 2; b) supervised training using manual and rating 5 video recordings not used in study; c) independent | NR | range ICC, .70-.98 (N=19) |
| OnePass coding system | McMaster | 2015 | Unclear | a) 3; b) extensively trained in motivational interviewing; coders were sent coding manual and 2 uncoded practice tapes to familiarize themselves with coding system; coding skills were not formally assessed; c) independent | NR | Range Fleiss’s kappa, -.195-.841, Md=.728 (across all 3 raters); Rater 4 vs. 5: range Cohen’s kappa, -.84-.84, Md=.57; Raters 4 vs. 6: range Cohen’s kappa, -.81-.78, Md=.61; Raters 5 vs. 6: range Cohen’s kappa, .37-.0.99, Md=.68 |
| Paediatric Dental Triadic Interaction Coding Scheme (PaeD-TrICS) | Yuan | 2019 | Yes | a) 2; b) 1 coder trained by other coder based on a coding manual; c) 1 calibration session, then independent | Cohen's kappa=.90 | Cohen's kappa=.83 |
| Parental Engagement Scale (PES) | Kearney | 2011 | Yes | a) 2; b) NR; c) independent | NR | 80% (type of statistic NR) |

| Instrument name (acronym) | First author | Year | Match intended-included sample | Coders a) N raters; b) training details; c) rating methods (independent, in consensus, other) | Intra-rater reliability Type of statistic (P-value) (N) | Inter-rater reliability Type of statistic (P-value) (N) |
| --- | --- | --- | --- | --- | --- | --- |
| Patient Involvement and Collaboration in Emergency care Teams (PIC-ET) tool | Dubois | 2023 | Unclear | a) 2; b) half-day training and test rating using 2 scenarios; c) NR | NR | Mean Cohen's kappa=.52, range ICC, .10-1.0 |
| Patient-Centered Behaviour Coding Instrument (PCBI) | Zandbelt | 2005 | Yes | a) 4; b) 15 recordings used for pilot-testing were used as ‘‘reference-tapes’’ for training; c) in consensus | NR | Range ICC, .22-.93 (relative agreement), .22-.92 (absolute agreement) |
| Patient-Centered Communication Coding System (PCCCS) | Ledoux | 2013 | Subset (authors report sample bias, with 83% Hispanic children) | a) 3; b) 24 h of training, led by principal investigator to learn identify utterances and apply codes. Coders practiced on eleven 5-minute excerpts of training audio files and discussed codes; c) 2 coders coded>20% of sessions (n=36) | ICC across sessions and within families: range, .45-.89, mean ICC=.75 (N, NR) | ICC: range, .34-.97, weighted kappa, M=.77 on behaviors/utterances; ICC=0-.67 on global ratings |
| Pediatric Emergency Discharge Interaction Coding Scheme (PEDICS) | Curran | 2017 | Yes | a) 2; b) NR; c) after every 2 inter-rater observation blocks, coders met with research team to review discrepancies and make modifications as needed | NR | Kappa scores≥.61 for 30 of 41 codes, all others<.61 |
| Pediatric Oncology Prognostic Communication (POPC) codebook | Kaye | 2023 | Yes | a) 4; b) NR; c) independent | NR | NR |
| Perioperative Child–Adult Medical Procedure Interaction Scale (P-CAMPIS) | Caldwell-Andrews | 2005 | Yes | a-b) NR; c) independent | NR | Mean Cohen's kappa=.87 (verbal codes, adult vocalizations), .92 (verbal codes, child vocalizations), .88 (nonverbal codes) (N=20) |
| Person Centered Behavior Inventory (PCBI) | Lann-Wolcott | 2011 | Yes | a) 2; b) NR; c) independent | NR | NR |
| Physician Recommendation Coding System (PhyReCS) | Scherr | 2017 | Subset | a) 6 (lead coder+5 assistants); b) research assistants received 30 h of guided practice over period of 2-3 weeks; c) research assistants double-coded random subset of 50 previously unseen transcripts to calculate reliability. Research assistants single-coded remaining encounters (n= 207). All coding was finished within 3 weeks, minimizing possibility of coder drift. | NR | Krippendorf’s alpha=.89 (95% CI, .86-.91 across 3 treatments); range, .64-.94 per treatment (N=50) |
| Problem, Analysis or Assessment, and Solution (Pas® System) | Van Mil | 1997 | n/a | n/a | NR | NR |
| Process of Interactional Sensitivity Coding in Healthcare (PISCH) | Sabee | 2015 | Unclear | a) 2; b) NR; c) in consensus | NR | Range Cohen’s kappa, .46-.72, range Scott's pi, .44-.72 (N=52, 2 raters) |

| Instrument name (acronym) | First author | Year | Match intended-included sample | Coders a) N raters; b) training details; c) rating methods (independent, in consensus, other) | Intra-rater reliability Type of statistic (P-value) (N) | Inter-rater reliability Type of statistic (P-value) (N) |
| --- | --- | --- | --- | --- | --- | --- |
| Refined Cue Utilization and Engagement in Dementia (CUeD) | Liu | 2020 | Yes | a) 4; b) trained through coding gold standard videos using coding manual developed based on refined CUED; then in multiple rounds each coded randomly selected videos independently and coding challenges were discussed; c) independent | NR | Part I: range percentage agreement, 95.93%-99.17% (*p*'s<.001, ± 1s tolerance), range Cohen’s kappa, .95-.99 (*p*'s<.001, 95% CI, .91-.99, ±1s tolerance); Part II: range percentage agreement, 94.51%-97.60% (*p*'s<.001, ± 1s tolerance), range Cohen’s kappa, .94-.97 (*p*'s<.001, 95% CI, .93-.98, ±1s tolerance); Part III: range percentage agreement, 93.63-96.70% (*p*'s<.001, ± 1s tolerance), range Cohen’s kappa, .93-.96 (*p*'s<.001, 95% CI, .92-.97, ±1s tolerance) (N=22) |
| Resident-centered Assessment of Interactions with Staff and Engagement tool (RAISE) | Snow | 2018 | Yes | a) 3 ; b) raters conducted co-ratings with first author; c) raters conducted observation sessions individually and in pairs (16 observation sessions on average per visit; range, 8–40). Observations took place in public areas. Raters completed field notes after observation sessions | NR | All Fleiss Kappas for all eight RAISE variables≥.70; agreement for codes aggregated into sub-groups>.90 |
| Rochester Participatory Decision-Making Scale (RPAD) | Shields | 2005 | Unclear | a-b) NR; c) unclear | Unclear | Unclear |
| Rochester Participatory Decision-Making Scale (RPAD) (Chinese) | Guo | 2020 | Subset | a-c) NR | Overall retest reliability coeﬃcient=.922, range retest reliability per item, .503-.952 | NR |
| Roter Interaction Analysis System (RIAS) * | Roter | 2002 | Unclear | Unclear | NR | Inter-rater correlation coefficient r, range for the categories, .68-1.0 (physicians), .60-1.0 (patients); range correlation for the clusters, .86-.99 (physicians), .76-.99 (patients); range correlations coefficients for global rating scales, .55-1.0 (physicians), .75-.86 (patients) (N=25) |
| SDM-18 | Salyers | 2012 | Yes | a) 7; b) NR; c) independent, followed by consensus | NR | Range percentage agreement, 67-100%, range Gwet’s agreement coefficient, .51–.97; range percentage agreement on who initiated initiated each element, 73-100%, range Gwet’s agreement coefficient, .66-.97; percentage agreement on level of agreement between consumer and provider regarding the decision, 100% |
| Segue framework | Makoul | 2001 | Yes | a) 2; b) criterion run: author and research assistant; test run: 2 naive coders who participated in 2 h training including videotaped examples of each task; c) independent, followed by consensus | Cohen's kappa=.99, range, .83-1.0 | Video sample: criterion run, range Cohen's kappa, .88-1.0, test run, M_kappa_=.93, range kappa, .33-1.0; audio sample, M_kappa_=.87 |
| Siminoff Communication Content & Affect Program (SCCAP) | Siminoff | 2011 | Subset | a) Sample 1: n=2, Sample 2: n=5; Sample 3: NR; b-c) NR | NR | Sample 1: .92, range, .80-.95, Sample 2: .92-1.0 (test statistic NR) |
| St Andrews Behavioural Interaction Coding Scheme (SABICS) | Zhou | 2012 | Yes | a) 2; b) NR, coders were instrument developers; c) independent | Range Cohen's kappa, .78-.88 | Range Cohen's kappa, .58-.66 |
| Instrument name (acronym) | **First author** | **Year** | **Match intended-included sample** | **Coders a) N raters; b) training details; c) rating methods (independent, in consensus, other)** | **Intra-rater reliability Type of statistic (P-value) (N)** | **Inter-rater reliability Type of statistic (P-value) (N)** |
| State Space Grids | Gainforth | 2019 | Yes | a) 2; b) 2 researchers w/ prior experience of coding smoking cessation behavioral support interactions using BCT taxonomies; c) Phase 1: 2 researchers coded transcribed segments; Phase 2: agreed codes for both practitioner and client were entered into coding software. | NR | Percentage agreement, 68.58% (provider), 77.39% (client) |
| Taxonomy of Verbal Response Modes (VRM) | Stiles | 1979 | Yes | a) 4; b) each coded and received feedback on 2 training transcripts; c) triple coding of each transcript. Agreement on form and intent of each utterance if 2/3 coders agreed. If all disagreed, form or intent assigned to "disagreement" category. | NR | Mean two-out-of-three agreement on codes for "form" of utterances, 99% and "intent", 95%, mean agreement between any two coders, 85% (form), 75% (intent) |
| Taxonomy of Verbal Response Modes (VRM) | Stiles | 1982 | Subset | a) 22; b) 30 h training; c) varying triads for coding each transcript; coders met weekly to discuss coding issues. If one coder agreed with the other two on <70%, the transcript was coded a 4th time. | NR | Of 60,914 utterances, 1.5% (forms)/3.1% (intents) coded as disagreements (test statistic NR) |
| Verona coding definitions of emotional sequences (VR-CoDES) | Eide | 2011 | Unclear | a) 2; b-c) NR | NR | Cohen's kappa=.64 (N=20) |
| Verona Coding Definitions of Emotional Sequences (VR-CoDES) | Vatne | 2010 | Unclear | a) 2; b) NR; c) independent (n=5 transcripts) | NR | Cohen's kappa=.64 (cues), 1.0 (concerns) (N=5) |
| Verona Coding Definitions of Emotional Sequences (VR-CoDES) | Wright | 2012 | Yes | a) 2; b) instrument developer trained the other coder; c) independent | ICC= .77 (95% CI, .69-.84) | ICC=.77 (95% CI, .64-.86) |
| Verona coding definitions of emotional sequences (VR-CoDES) | Zimmermann | 2011 | Subset | a) multiple (not specified); b-c) NR | NR | Proportion agreement, 81.46%, Cohen’s kappa=.70 (*p*<.0001) (N=20) |
| Verona Coding Definitions of Emotional Sequences (VR-CoDES) - Chinese | Yin | 2019 | No | a) 2; b) training provided by 2 developers of the original VR-CoDES. Certification received; c) independent | NR | ICC=.79 |
| Verona Coding Definitions of Emotional Sequences for client cues and concerns (VR-CoDES- CC) | Vijfhuizen | 2017 | Unclear | a) 2; b) formally or self-trained using Verona Network on Sequence Analysis training package; c) independent | NR | Cue identification: agreement, 75.0%, Cohen's kappa=.50, cue specification: agreement, 88.3%, kappa=.82, perspective and direction of cue/concern, agreement, 83.3%, kappa=.74 , cue elicitation: agreement, 79.8%, kappa=.69 (*p*'s<.000) (N=6) |
| Verona Coding Definitions of Emotional Sequences for health provider responses (VR-CoDES-P) | Del Piccolo | 2011 | Subset | a) 2; b) NR; c) independent | NR | Percentage agreement, 92.9%, Cohen’s kappa=.90 (±0.04) (*p*<.0001) (N=2) |
| Instrument name (acronym) | **First author** | **Year** | **Match intended-included sample** | **Coders a) N raters; b) training details; c) rating methods (independent, in consensus, other)** | **Intra-rater reliability Type of statistic (P-value) (N)** | **Inter-rater reliability Type of statistic (P-value) (N)** |
| Verona Coding Definitions of Emotional Sequences for health provider responses (VR-CoDES-P) | Wright | 2012 | Yes | a) 2; b) instrument developer trained the other coder; c) independent | ICC=.93 (providing/reducing space, 95% CI, .90-.96) and .82 (explicit/non-explicit, 95% CI, .72-.88) | ICC=.88 (95% CI, .80-.93) |
| Verona Coding Definitions of Emotional Sequences for health provider responses (VR-CoDES-P) - Chinese | Yin | 2019 | No | a) 2; b) training provided by 2 developers of the original VR-CoDES. Certification received; c) independent | NR | NR |
| Verona coding definitions of emotional sequences to code health providers’ responses (VR-CoDES-P) | Vijfhuizen | 2017 | Unclear | a) 2; b) NR; c) independent | NR | Percentage agreement, 92.9%, Cohen’s kappa=.90 (±0.04) (*p*<.0001) (N=2) |
| Verona Medical Interview Classification System (VR-MICS) | Del Piccolo | 2004 | Subset | NR | NR | Cohen's kappa=0.93 (provider), 0.85 (patient) |
| Verona medical interview classification system (VR-MICS) | Del Piccolo | 2005 | Yes | a) 2; b) 2 week training; c) independent; 1 rater rated transcripts again after 3 months | Percentage agreement, 88.56% (N=30) | Percentage agreement, 82.38, Cohen's kappa=.85 (N=30) |
| Vr-COPE | McDermott | 2021 | Yes | a) 1; b-c) NR | NR | NR |

**Notes.**

*The original publication could not be retrieved. Information was based on Roter et al. (2002) and Ong et al. (1998)

Abbreviations: CI, Confidence interval; ICC, Intraclass correlation coefficient; NR, not reported; r_P_, Pearson's correlation coefficient; r_S_, Spearman's rho coefficient; T, test statistic T represents a modified Cohen’s kappa using theoretical assumptions rather than empirical data to estimate expected values
