# Supplementary material for: Systematic review of observational coding systems to assess patient-healthcare professional interactions
Source: Patient Educ Couns. Author manuscript; Available in PMC 2026 Aug 16. (PMC13477582; doi:10.1016/j.pec.2025.108718)
Supplement: Supplementary file 5 [file NIHMS2172109-supplement-5.docx]

Appendix E / Supplementary Table 2b. Evaluation of psychometric properties relating to validity, consistency, and cross-cultural adaptation of the coding schemes.

| Instrument name (acronym) | First author | Year | Content validity^a^ | Structural validity^b^ | Internal consistency^c^ | Cross-cultural  adaptation | Cross-cultural  validity | Criterion validity^d^ | Convergent validity^e^ | Discriminative validity^f^ | Known‐groups validity^g^ |
| --- | --- | --- | --- | --- | --- | --- | --- | --- | --- | --- | --- |
| 5 As for Physical Activity | Carroll | 2011 | NR | NR | NR | n/a | n/a | NR | NR | NR | NR |
| 5a-DOC | Lawson | 2009 | NR | NR | NR | n/a | n/a | NR | NR | NR | NR |
| Active Patient Participation Coding (APPC) scale | Street | 2005 | NR | NR | NR | n/a | n/a | NR | NR | NR | NR |
| Advance Care Planning Communication Assessment Tool (ACP-CAT) | Yuen | 2020 | NR | NR | NR | n/a | n/a | a) summative item 21 (overall impression of ACP communication skills) and 22 (overall ability to respond to emotion); b) item 21: r=.87, *p*<.001, item 22: r=.72, *p*<.001 | NR | NR | a) NR; b) before vs. after advance care planning communication training program; c) increase in overall scores pre- to post-training (Md=6-11; *p*<.001), and for all sub-scores except step 1 |
| Analysis System for Self-Efficacy Training (ASSET) | Zinken | 2008 | NR | NR | NR | n/a | n/a | NR | NR | a) yes; b) n/a; c) single self-efficacy orientated utterances shorter than non-self-efficacy utterances (*p*<.001) (hypotheses confirmed). As the program progresses less time was left for participants to talk (*p*<.05) (hypotheses not confirmed) | NR |
| Assessment of Brief Behavioural Change Counselling’ tool (ABC tool) | Fouche | 2020 | NR | NR | a) α; b) range, .778-.942 (5 dimensions), .612-.959 (individual items) | n/a | n/a | NR | NR | NR | NR |

| Instrument name (acronym) | First author | Year | Content validity^a^ | Structural validity^b^ | Internal consistency^c^ | Cross-cultural  adaptation | Cross-cultural  validity | Criterion validity^d^ | Convergent validity^e^ | Discriminative validity^f^ | Known‐groups validity^g^ |
| --- | --- | --- | --- | --- | --- | --- | --- | --- | --- | --- | --- |
| Behavioral Health Treatment Model for patients with Medically Unexplained Symptoms (BHTM-MUS) | Grayson-Sneed | 2018 | NR | NR | n/a | n/a | n/a | NR | NR | NR | NR |
| Behavioral Test of Interpersonal Skills (BTIS) | Olson | 1991 | Each patient statement coded using BTIS categories of patient situations. The patients presented mostly pain situations and no aggression situations, while BTIS percentages were divided more evenly between patient situations. | NR | n/a | n/a | n/a | NR | a) yes; b) correlations between patients opportunities and providers' responses; c) results NR (hypotheses not confirmed) | NR | NR |
| Behaviour Change Counselling Index (BECCI) | Lane | 2005 | Content validity repeatedly checked by obtaining structured feedback from N=9 experts in BCC, which led to adaptations in item selection | NR | a) α; b) .71 (baseline), .63 (final consultations). Mean inter-item correlation=.22 (baseline), .14 (final consultations) | n/a | n/a | NR | NR | NR | NR |
| Brief Decision Support Analysis Tool (DSAT-10) | Stacey | 2008 | NR | NR | NR | n/a | n/a | NR | NR | NR | NR |

| Instrument name (acronym) | First author | Year | Content validity^a^ | Structural validity^b^ | Internal consistency^c^ | Cross-cultural  adaptation | Cross-cultural  validity | Criterion validity^d^ | Convergent validity^e^ | Discriminative validity^f^ | Known‐groups validity^g^ |
| --- | --- | --- | --- | --- | --- | --- | --- | --- | --- | --- | --- |
| Cancode interaction analysis system (Cancode) | Dent | 2005 | NR | a) yes; b) 30; c) 2; d) 63%; e) .41-.92 | NR | n/a | n/a | NR | NR | NR | a) doctor verbal behavior (doctor-centeredness, provision of medical information, psycho-social exchange) changes in response to patient conditions (active, passive, intermediate), Cancode is sensitive to these changes; b) active patient vs. passive patient vs. patient who asks some questions and wants to make decision jointly; c) differences between patient types for “total verbal control” (F(2,29)=33.22, *p*=.000) and “total verbal support” (F(2,29)=9.58, *p*=.001) (principal component analysis, 10 verbal scores) (hypotheses confirmed) |
| Caring Behavior Coding Scheme (CBCS) | Martensson | 2021 | Content validity was examined by author of Swanson’s Theory of Caring, who confirmed that CBCS captures the theory content. 2 experienced nurses not involved in the study confirmed that CBCS adequately captured caring behaviors and that codes differentiated caring and non-caring interactions. | NR | NR | n/a | n/a | NR | NR | NR | NR |

| Instrument name (acronym) | First author | Year | Content validity^a^ | Structural validity^b^ | Internal consistency^c^ | Cross-cultural  adaptation | Cross-cultural  validity | Criterion validity^d^ | Convergent validity^e^ | Discriminative validity^f^ | Known‐groups validity^g^ |
| --- | --- | --- | --- | --- | --- | --- | --- | --- | --- | --- | --- |
| Chronic Pain Coding System (CPCS) | Henry | 2016 | NR | NR | a) α; b) .75 | n/a | n/a | NR | a) yes; b) baseline pain interference (SF-12 pain item), visit difficulty (physician assessment); c) Pain interference: longer pain discussions, incidence rate ratio (IRR)=1.51, 95% CI=1.12-2.04, *p*=.007 (hypotheses confirmed), negative patient assessments: IRR=1.34, 95% CI=0.90-2.02, *p*=.15 (hypotheses not confirmed), pain-related requests: IRR=1.32, 95% CI=1.04-1.76, *p*=.02 (hypotheses confirmed) ), Visit difficulty: patient resistance/disagreement, linear regression coefficient=0.54, 95% CI=0.18-0.90, *p*=.003 (hypotheses confirmed), pain-related requests, linear regression coefficient=0.11, 95% CI=-0.02-0.24, *p*=.11 (hypotheses not confirmed), longer pain discussions, linear regression coefficient=0.07, 95% CI=0.00-0.15, *p*=.049 (hypotheses confirmed) | NR | NR |
| Coding System for Staff-Patient Interactions (CSSPI) | Kolko | 1989 | NR | NR | NR | n/a | n/a | NR | NR | NR | NR |
| Content coding for contextualization of care (4C) | Weiner | 2014 | NR | NR | NR | n/a | n/a | NR | NR | NR | NR |
| Instrument name (acronym) | First author | Year | Content validity^a^ | Structural validity^b^ | Internal consistency^c^ | Cross-cultural  adaptation | Cross-cultural  validity | Criterion validity^d^ | Convergent validity^e^ | Discriminative validity^f^ | Known‐groups validity^g^ |
| Davis Observation Code (DOC) | Callahan | 1991 | NR | NR | NR | n/a | n/a | NR | a) no; b) chart audits based on same operational definitions of physician behaviors; c) range phi, .12-.49, *p*>.05 (N=49 visits) | NR | NR |
| Decision Analysis System for Oncology (DAS-O) | Brown | 2011 | Items, explanation and rationale for inclusion, characteristic examples, and development process were presented to multidisciplinary panel (N=27); consensus was reached on adequacy and completeness of items, explanations and examples. | NR | NR | n/a | n/a | NR | a) yes; b) OPTION^12item^ (revised), DSAT; c) correlation DAS-O - OPTION^12item^ (revised)=.73, DAS-O - DSAT, .58 (N=55 consultations) (hypotheses confirmed) | NR | NR |
| Decision Support Analysis Tool (DSAT) | Butow | 2010 | NR | NR | NR | n/a | n/a | NR | a) no; b) OPTION^12item^ (revised), DAS-O; c) r*P*=.594 (OPTION^12item^ revised), .585 (DAS-O) (*p*'s<.01) (N=55) | NR | NR |

| Instrument name (acronym) | First author | Year | Content validity (item pool relevance / comprehensiveness) | Structural validity a) reflective model; b) N; c) # factors; d) % variance explained; e) range factor loadings | Internal consistency a) type of scale; b) results | Cross-cultural  adaptation | Cross-cultural  validity | Criterion validity a) gold standard / comparator; b) results | Convergent validity a) clear hypotheses; b) comparator; c) results | Discriminative validity a) clear hypotheses; b) comparator; c) results | Known‐groups validity a) hypotheses; b) subgroups; c) results |
| --- | --- | --- | --- | --- | --- | --- | --- | --- | --- | --- | --- |
| Decision Support Analysis Tool (DSAT) | Guimond | 2003 | NR | NR | NR | n/a | n/a | NR | a) yes; b) Satisfaction with decision making process, Satisfaction with decision, Decisional Conflict Scale, Provider Decision Process Assessment Instrument (both for physician and patient); c) DSAT subscale decision support: PHYSICIAN outcome: Satisfaction with decision making process, r_P_=.39 (hypotheses confirmed); Satisfaction with decision, r_P_=-.02 (hypotheses not confirmed); Lower decisional conflict, r_P_=.06 (hypotheses not confirmed); PATIENT outcome: Satisfaction with decision making process, r_P_=.04 (hypotheses not confirmed); Satisfaction with decision, r_P_=.52 (hypotheses confirmed); Lower decisional conflict, r_P_=.36 (hypotheses confirmed); DSAT subscale communication skills: PHYSICIAN outcome: Satisfaction with decision making process, r_P_=.41 (hypotheses confirmed); Satisfaction with decision, r_P_=-.01 (hypotheses not confirmed), Lower decisional conflict, r_P_=.09 (hypotheses not confirmed); PATIENT outcome: Satisfaction with decision making process, r_P_=.06 (hypotheses not confirmed); Satisfaction with decision, r_P_=.52 (hypotheses confirmed), Lower decisional conflict, r_P_=.36 (hypotheses confirmed) | NR | a) more observed skills in physician-patient interactions with patients prepared for consultation using decision aid and accompanying worksheet vs. general education pamphlet; b) patients prepared for consultation using decision aid and worksheet vs. general education pamphlet; b) Decision support skills, Md=218 (decision aid) vs. 129 (pamphlet) (Wilcoxon W-test, *p*=.11); Communication skills, Md=223 (decision aid) vs. 134 (pamphlet) (Wilcoxon W-test, *p*=.10) (hypotheses confirmed) |

| Instrument name (acronym) | | First author | Year | Content validity^a^ | Structural validity^b^ | Internal consistency^c^ | Cross-cultural  adaptation | Cross-cultural  validity | Criterion validity^d^ | Convergent validity^e^ | Discriminative validity^f^ | Known‐groups validity^g^ |
| --- | --- | --- | --- | --- | --- | --- | --- | --- | --- | --- | --- | --- |
|  | Dementia Competent Social Interaction (DSI) and Culturally Competent Social Interaction (CSI) Coding Schemas | Kim | 2012 | Gerontological experts scored each item of DSI and CSI for relevance (0, not relevant to 4, very relevant). A content validity index was computed as alpha coefficient over relevance scores (0, lack of agreement to 1, complete agreement). CVI scores, .88 (DSI), .80 (CSI) | NR | NR | n/a | n/a | NR | NR | NR | NR |
|  | Detail of essential elements and participants in shared decision making (DEEP-SDM) | Clayman | 2012 | NR | NR | NR | n/a | n/a | NR | NR | NR | NR |
|  | Elements of Informed Decision Making (IDM) | Braddock | 1999 | NR | NR | NR | n/a | n/a | NR | NR | NR | NR |

| Instrument name (acronym) | First author | Year | Content validity^a^ | Structural validity^b^ | Internal consistency^c^ | Cross-cultural  adaptation | Cross-cultural  validity | Criterion validity^d^ | Convergent validity^e^ | Discriminative validity^f^ | Known‐groups validity^g^ |
| --- | --- | --- | --- | --- | --- | --- | --- | --- | --- | --- | --- |
| Empathic and Potential Empathic opportunity (E-PE-O) method | Eide | 2004 | NR | NR | NR | n/a | n/a | a) RIAS categories: 'shows concerns or worries', 'shows optimism', 'reassurance', 'asks for reassurance'; b) raters identified 29 EOs (N=39 consultations). Corresponding RIAS category was most often 'concerns', 'reassurance' was also scored. Raters identified 121 PEOs; 53 scored with RIAS as 'concerns', 29 as 'medical information', 11 as 'reassurance', 8 as 'asks for reassurance', and 16 as 'other psychosocial topics'. | NR | NR | NR |

| Instrument name (acronym) | First author | Year | Content validity^a^ | Structural validity^b^ | Internal consistency^c^ | Cross-cultural  adaptation | Cross-cultural  validity | Criterion validity^d^ | Convergent validity^e^ | Discriminative validity^f^ | Known‐groups validity^g^ |
| --- | --- | --- | --- | --- | --- | --- | --- | --- | --- | --- | --- |
| Empathic Communication Coding System (ECCS) | Bylund | 2002 | NR | NR | NR | n/a | n/a | NR | NR | NR | a) Hypothesis 1: female patients create greater number of empathic opportunities than male patients. Hypothesis 2: female patients name emotions in greater proportion of empathic opportunities than male patients. Hypothesis 3: female patients create more intense empathic opportunities than male patients. Hypothesis 4: female physicians’ responses to empathic opportunities are more empathic than male physicians’ responses; b) female vs. male patients, female vs. male physicians; c) Hypotheses 1-2 not confirmed, Hypothesis 3 (*p*<.05) and 4 (*p*<.01) confirmed: emotional intensity score, M=3.41 (female patients) vs. 3.14 (male patients), t=-2.08, *p*<.05, empathy score, M=3.27 (female physicians) vs. 2.90 (male physicians), t=-2.62, *p*<.01 |
| eSEGUE | Assis-Hassid | 2015 | Expert panel assessed item relevance (phase 1, N=48) and relevance and comprehensiveness (phase 2, N=8). Modifications were made after each round | NR | NR | n/a | n/a | NR | NR | NR | NR |
| Evidence-Based Patient-Centered Interviewing Method | Grayson-Sneed | 2017 | NR | NR | n/a | n/a | n/a | NR | NR | NR | NR |
| Instrument name (acronym) | First author | Year | Content validity^a^ | Structural validity^b^ | Internal consistency^c^ | Cross-cultural  adaptation | Cross-cultural  validity | Criterion validity^d^ | Convergent validity^e^ | Discriminative validity^f^ | Known‐groups validity^g^ |
| Four Habits Coding Scheme (4-HCS) | Catani | 2018 | 3 professors experienced in teaching communication skills assessed 4 videos of real consultations, and participated in a cognitive interview. They then participated in retrospective interview assessing comprehension, applicability and relevance of items and behavior categories, and provided suggestions for improvement | NR | NR | 5-step extensive, iterative process involving 4 translators, 8 experts, original measure developer, and authors | Used modified Delphi technique; no comparison made between samples | NR | NR | NR | NR |
| Four Habits Coding Scheme (4-HCS) | Jensen | 2010 | NR | NR | NR | n/a | n/a | NR | NR | NR | NR |
| Four Habits Coding Scheme (4-HCS) | Krupat | 2006 | NR | NR | a) α; b) Habits 1–4: .71, .51, .81, .61 | n/a | n/a | NR | a) no; b) 8 RIAS categories, back channel responses, non-verbal measures, patient post-visit evaluation domains; c) each Habit significantly correlated with ≥1 RIAS item (*p*<.05 to <.01), Habits 2-4 significantly correlated with back channel responses (*p*<.01), Habit 3 correlated significantly with each non-verbal measure (*p*<.01), correlations Habits and post-visit evaluation domain n.s. | NR | NR |

| Instrument name (acronym) | First author | Year | Content validity^a^ | Structural validity^b^ | Internal consistency^c^ | Cross-cultural  adaptation | Cross-cultural  validity | Criterion validity^d^ | Convergent validity^e^ | Discriminative validity^f^ | Known‐groups validity^g^ |
| --- | --- | --- | --- | --- | --- | --- | --- | --- | --- | --- | --- |
| Four Habits Coding Scheme (4-HCS) / Physician–patient relationship competence assessment scale | Bellier | 2020 | NR | NR | a) item-rest correlations, α; b) all but item 16 fulfilled internal consistency criterion (item-rest correlation>.40). α=.94 (range, .72-.88 across subscales). Removing item 16 from “Invest in the end” habit subscale improved α from .88 to .90. | Translated and cross-culturally adapted into French by language service, according to published guidelines for forward-backward translations | Confirmatory factor analysis of cross-cultural adaptation indicated unsatisfactory fit with internal 4-factor structure of original version (CFI=.79, SRMR=0.09, RMSEA=.12). Exploratory Factor Analysis using orthogonal rotation identified 4 factors with Eigenvalues>1.0, explaining 66.8% of variance; visual inspection suggested unidimensionality. EFA resulted in merging 3 habits (Invest in beginning, Elicit patient's perspective, Demonstrate empathy). | NR | a) no; b) physician–patient relationship competence assessment scale scores; c) most pairwise r_P_ between habit and physician–patient relationship competence assessment scale scores >.70 (range, .47-.92), indicating satisfactory convergent validity | NR | a) mean scores on 4-HCS are higher for medical consultations recorded during second vs. first semester; b) first and second semester video-recorded medical consultations; c) medical students recorded in second semester yielded higher scores compared to first semester recordings for all four habits (*p*<.001) (hypotheses confirmed) |

| Instrument name (acronym) | First author | Year | Content validity^a^ | Structural validity^b^ | Internal consistency^c^ | Cross-cultural  adaptation | Cross-cultural  validity | Criterion validity^d^ | Convergent validity^e^ | Discriminative validity^f^ | Known‐groups validity^g^ |
| --- | --- | --- | --- | --- | --- | --- | --- | --- | --- | --- | --- |
| Global Behavior Scale (GBS) | Lann-Wolcott | 2011 | NR | NR | a) α; b) .95 | n/a | n/a | NR | a) yes; b) PCBI; c) r_P_=0.67, *p*<.01 (hypotheses confirmed) | a) yes; b) Task-Centered Behavioral Inventory (TCBI); c) r_P_=-.56 (*p*<.01) (hypotheses confirmed) | NR |
| Health Coaching Index (HCI) | Sohl | 2021 | Subject matter experts (behavioral research/health coaching) reviewed original item pool and improved content validity of items. Cognitive interviews were conducted to refine items. | NR | NR | n/a | n/a | NR | a) yes; b) RIAS; c) all associations were small to moderate in expected direction, except for HCI and RIAS scores for patient-centeredness, open-ended questions and dominance (n.s.) | NR | NR |
| Health Professional-Geriatric Patient Interaction Behavior Rating Code | Adelson | 1982 | NR | NR | a) intercorrelations between behaviors included in coding measure; b) all but one (r=.31) intercorrelation significant (r=.37-.94; *p*<.05 to *p*<.001) | n/a | n/a | a) behavioral category frequencies correlated with supervisor ratings; b) correlations for all categories with supervisor ratings all significant (*p*'s<.001). Those most predictive were: engages patient in conversation, gives procedural information, smiles, asks for feedback, uses patient's name. | NR | NR | NR |

| Instrument name (acronym) | First author | Year | Content validity^a^ | Structural validity^b^ | Internal consistency^c^ | Cross-cultural  adaptation | Cross-cultural  validity | Criterion validity^d^ | Convergent validity^e^ | Discriminative validity^f^ | Known‐groups validity^g^ |
| --- | --- | --- | --- | --- | --- | --- | --- | --- | --- | --- | --- |
| Indicators of Health Behavior Change (IHBC) | Potempa | 2023 | Content validity was established with independent experts (nursing/behaviorist faculty members) not involved in development of indicators. | a) yes; b) 59 over 3 time points; c) 2; d-e) NR | NR | n/a | n/a | NR | NR | NR | NR |
| Informed Decision Making Measure (IDM) | Leader | 2012 | NR | NR | a) α; b) .80 | n/a | n/a | NR | NR | NR | NR |
| Informed Decision Making Measure (IDM) | Weiss | 2008 | NR | NR | NR | n/a | n/a | NR | a) yes; b) OPTION^12item^ (revised); c) r_S_=.14, Bland-Altman test of mean difference: mean difference score=1.11 (CI, 0.66–1.56), Limits of Agreement=-3.94 - 6.16 (hypotheses confirmed) | NR | NR |
| Kin interaction coding system (KINcode) | Laidsaar-Powell | 2015 | Coding system was cross-checked with other instruments for comprehensiveness | NR | NR | n/a | n/a | NR | NR | NR | NR |
| Liverpool-MAAS (LIV-MAAS) | Enzer | 2003 | NR | NR | NR | n/a | n/a | NR | NR | NR | NR |
| Manchester Observation Code (MOC) for genetic counseling | Liede | 2000 | NR | NR | NR | n/a | n/a | NR | NR | NR | NR |

| Instrument name (acronym) | First author | Year | Content validity^a^ | Structural validity^b^ | Internal consistency^c^ | Cross-cultural  adaptation | Cross-cultural  validity | Criterion validity^d^ | Convergent validity^e^ | Discriminative validity^f^ | Known‐groups validity^g^ |
| --- | --- | --- | --- | --- | --- | --- | --- | --- | --- | --- | --- |
| Mappin'SDM | Kasper | 2012 | NR | NR | NR | n/a | n/a | NR | a) yes; b) subjective judgements of SDM (patient-/doctor-reported); c) objective - subjective judgements, rP=.14, *p*=.4 (doctor/observer), r_P_=-.22, *p*=.19 (patient/observer) (n.s.) (hypotheses confirmed). Observer ratings for 3 different foci, r_P_=.64, *p*<.001 (doctor/patient), .96, *p*<.001 (doctor/dyad), .80, *p*<.001 (patient/dyad) (hypotheses confirmed). SDM performance and patient activity did not predict inter-relatedness of measurement perspectives (*p*'s n.s.) (hypotheses not confirmed) | NR | NR |

| Instrument name (acronym) | First author | Year | Content validity^a^ | Structural validity^b^ | Internal consistency^c^ | Cross-cultural  adaptation | Cross-cultural  validity | Criterion validity^d^ | Convergent validity^e^ | Discriminative validity^f^ | Known‐groups validity^g^ |
| --- | --- | --- | --- | --- | --- | --- | --- | --- | --- | --- | --- |
| Mappin'SDM (Norwegian) | Kienlin | 2017 | NR | NR | NR | Forward-backward translation, refinement and consensus in research panel including author of original instrument | NR | a) senior researcher experienced in SDM training provided dichotomous rating (SDM present/absent), analyses of average and item-level sensitivity and specificity; b) sensitivity: MAPPINdoctor=78 (range, 46-100%), MAPPINpatient=81 (range, 50-100%), MAPPINdyad=93 (range, 81-100%); specificity: MAPPINdoctor=91 (range, 78-100%), MAPPINpatient=90 (range, 63-100%), MAPPINdyad=80 (range, 75-100%) | a) no; b) OPTION^5item^; c) MAPPINdoctor: r_S_=.44, MAPPINpatient: r_S_=.38; MAPPINdyad: r_S_=.40 | NR | NR |
| Measure of Patient-Centered Communication (MPCC) | Shields | 2005 | NR | NR | NR | n/a | n/a | a) patients’ perceptions of relationship with physicians; b) physicians’ % emotion words related to actual patient ratings of physicians on autonomy support (β=.07, *p*<.05), knowledge of patient (β =.07, *p*<.05), patients’ trust in physician (β =.08, *p*<.01), patients’ satisfaction (β =.08, *p*<.05) | a) yes; b) MPCC human coding; c) correlation (unspecified): .14 (MPCC full scale), range, -.06-.19 (subscales) (n.s.) (hypotheses not confirmed) | NR | NR |
| Instrument name (acronym) | First author | Year | Content validity^a^ | Structural validity^b^ | Internal consistency^c^ | Cross-cultural  adaptation | Cross-cultural  validity | Criterion validity^d^ | Convergent validity^e^ | Discriminative validity^f^ | Known‐groups validity^g^ |
| Medical Interaction Process System (MIPS) | Ford | 2000 | NR | NR | NR | n/a | n/a | NR | a) no; b) comparable clinician/patient content categories of RIAS; c) range r_S,_ -.51-.80 (clinician modes/content categories), -.25-.87 (patient modes/content categories); range clinician global ratings, -.33-.45 (with RIAS clinician ratings), -.51-.20 (with RIAS patient ratings); range patient global ratings: -.44-.24 (with RIAS clinician ratings), -.43-.65 (with RIAS patient ratings). Authors report most relevant coefficients to exceed 0.50 yet not which ones. | NR | NR |

| Instrument name (acronym) | First author | Year | Content validity^a^ | Structural validity^b^ | Internal consistency^c^ | Cross-cultural  adaptation | Cross-cultural  validity | Criterion validity^d^ | Convergent validity^e^ | Discriminative validity^f^ | Known‐groups validity^g^ |
| --- | --- | --- | --- | --- | --- | --- | --- | --- | --- | --- | --- |
| Medical Interaction Process System (MIPS) | Ford | 2004 | NR | NR | NR | n/a | n/a | NR | NR | NR | a) skilled doctors exhibit more disclosure-promoting behaviors and fewer inhibitory behaviors than less skilled doctors; b) doctors whom senior lecturer considered more vs. less effective communicators; c) skilled doctors showed higher ratios of patient:disease focus and psychosocial:physical focus than less skilled doctors (*p*<.01), skilled doctors engaged in more checking and summarizing behavior, registering patient information, non-physical counselling, empathy and support, non-physical focused open and closed questions and reflective question asking than less skilled doctors (*p*<.01), skilled doctors scored higher on eye contact, patient centeredness and sensitivity than less skilled doctors (*p*<.01) (hypotheses confirmed) |

| Instrument name (acronym) | First author | Year | Content validity^a^ | Structural validity^b^ | Internal consistency^c^ | Cross-cultural  adaptation | Cross-cultural  validity | Criterion validity^d^ | Convergent validity^e^ | Discriminative validity^f^ | Known‐groups validity^g^ |
| --- | --- | --- | --- | --- | --- | --- | --- | --- | --- | --- | --- |
| MEDICODE | Richard | 2006 | NR | NR | n/a | n/a | n/a | NR | a) no; b) Medical Interview Satisfaction Scale; c) all r_P_ positive; significant r_P_ between discussions of Observed effects on symptoms in MEDICODE and effects of medication and health expectations questions (*p*<.427, *p*<.444, *p*<.05) and between discussions of instructions and information received and health expectations (*p*<.419, *p*<.500, *p*<.05). Discussion of side effects n.s. | NR | NR |
| Moffitt Accrual Analysis System (MAAS) | Albrecht | 1999 | NR | NR | NR | n/a | n/a | NR | a) yes; b) inter-correlations between MAAS scale items; c) range correlations between coder judgments concerning hierarchical rapport between physician and patient, connection/closeness between physician and patient, trust, and physician responsiveness to patient concerns, .69-.78 (*p*<.01), negative association between judgments presenting a primarily data-based style to judgments primarily based on their personal opinions, r=-.33 (*p*< .05) (hypotheses confirmed) | a) yes; b) RIAS; c) range correlations, -.27-.22 (n.s.) (hypotheses confirmed) | NR |
| Motivational Interviewing Skills Code - modified (MISC 2.1) | Lord | 2015 | NR | NR | NR | n/a | n/a | NR | NR | NR | NR |
| Instrument name (acronym) | First author | Year | Content validity^a^ | Structural validity^b^ | Internal consistency^c^ | Cross-cultural  adaptation | Cross-cultural  validity | Criterion validity^d^ | Convergent validity^e^ | Discriminative validity^f^ | Known‐groups validity^g^ |
| Motivational Interviewing Skills Code (MISC) | Moyers | 2003 | NR | NR | NR | n/a | n/a | NR | NR | NR | NR |
| Motivational Interviewing Treatment Integrity (MITI) rating system | McMaster | 2015 | NR | NR | NR | n/a | n/a | NR | NR | NR | NR |
| Motivational Interviewing Treatment Integrity Code (MITI) rating system (Swedish) | Forsberg | 2007 | NR | NR | NR | Forward-backward translation, with involvement of original instrument author | NR | NR | NR | NR | NR |
| Motivational Interviewing Treatment Integrity Code (MITI) rating system (Swedish) | Forsberg | 2008 | NR | a) no; b) 120; c) 3; d) 66.6%; e) .415-.914 | NR | n/a | n/a | NR | NR | NR | a) higher scores on MITI 1. in sessions completed after vs. before MI training and 2. in MI vs. advice group; b) sessions completed before vs. after MI training, MI vs. advice group; b) sessions before vs. after Mi-training: differences in Empathy (*p*=.028), Non-adherent statements (*p*=.043), Total reflections (*p*=.028); Complex reflections and MI spirit had changed in hypothesized direction but n.s.; Advice vs. MI groups: differences between groups for Empathy, MI spirit, Non-adherent utterances, and Simple, Complex, and Total reflections (*p*'s<.001) (hypotheses confirmed) |
| Multi-Dimensional Analysis of Patient Outcome Predictions (MD.POP) | Ménard | 2018 | NR | NR | NR | n/a | n/a | NR | NR | NR | NR |

| Instrument name (acronym) | First author | Year | Content validity^a^ | Structural validity^b^ | Internal consistency^c^ | Cross-cultural  adaptation | Cross-cultural  validity | Criterion validity^d^ | Convergent validity^e^ | Discriminative validity^f^ | Known‐groups validity^g^ |
| --- | --- | --- | --- | --- | --- | --- | --- | --- | --- | --- | --- |
| Nonverbal Accommodation Analysis System (NAAS) | D'Agostino | 2011 | NR | NR | n/a | n/a | n/a | NR | a) no; b) MIPS affective global rating codes and body language code of eye contact; c) range r_P,_ .295-.623 (*p*<.05 on 13/20 subscales) | NR | NR |
| Nonverbal communication in doctor–elderly patient transactions (NDEPT) | Gorawara-Bhat | 2007 | NR | NR | NR | n/a | n/a | NR | NR | NR | NR |
| NR ("Strength of connectional silence" taxonomy) | Bartels | 2016 | NR | NR | NR | n/a | n/a | NR | NR | NR | NR |
| NR (ambivalence and reluctance in palliative care ) | Gupta | 2023 | NR | NR | n/a | n/a | n/a | a) decision making; b) when encounters contained ambivalence between patients and loved ones, ratio of decisions made was lower (r=-0.29, *p*=.06). Association within-patient ambivalence or reluctance with decision making n.s. | NR | NR | NR |

| Instrument name (acronym) | First author | Year | Content validity^a^ | Structural validity^b^ | Internal consistency^c^ | Cross-cultural  adaptation | Cross-cultural  validity | Criterion validity^d^ | Convergent validity^e^ | Discriminative validity^f^ | Known‐groups validity^g^ |
| --- | --- | --- | --- | --- | --- | --- | --- | --- | --- | --- | --- |
| NR (Automatic annotation of information-giving in clinical conversations) | Mayfield | 2014 | NR | NR | NR | n/a | n/a | NR | NR | a) no; b) Comprehensive Analysis of the Structure of Encounters System (CASES) coding scheme; c) distinct patterns of information-giving ratio coded automatically occur during distinct processes (e.g., presentation) of consultation (manually coded using CASES), which seem intuitively correct differences (N=50) | NR |
| NR (behavior change therapy delivery and receipt) | Hoekstra | 2022 | NR | NR | NR | n/a | n/a | NR | NR | NR | NR |
| NR (Caregiver Activation in Advanced Cancer Care) | Dingley | 2017 | NR | NR | NR | n/a | NR | NR | NR | NR | NR |
| NR (Characterize client responses to practitioner-delivered behaviour change techniques) | Gainforth | 2016 | NR | NR | NR | n/a | n/a | NR | NR | NR | NR |
| NR (coding frame for shared decision-making) | Singh | 2010 | NR | a) assumed; b) 63; c) 3 (discussions of treatment, of evidence, of patient challenges), different from factors initially assumed; d) 44.6%; e) NR | a) α; b) .77 (total scale) | n/a | n/a | NR | a) yes; b) patient-reported achievement of involvement preference; c) interaction between involvement preference and SDM behavior on achievement of preference: odds ratio=1.45, 95% CI, 1.03–2.04 (hypotheses confirmed) | NR | NR |
| Instrument name (acronym) | First author | Year | Content validity^a^ | Structural validity^b^ | Internal consistency^c^ | Cross-cultural  adaptation | Cross-cultural  validity | Criterion validity^d^ | Convergent validity^e^ | Discriminative validity^f^ | Known‐groups validity^g^ |
| NR (coding of Patient-provider self-care communication and EMR usage) | Arar | 2006 | NR | NR | NR | n/a | n/a | NR | NR | NR | NR |
| NR (coding scheme to identify the content of general practitioner communication) | Di Caccavo | 2000 | NR | NR | NR | n/a | n/a | NR | NR | NR | NR |
| NR (Cognitive strategies used by General Practitioners in diagnostic reasoning) | Donner-Banzhoff | 2017 | NR | NR | NR | n/a | n/a | NR | NR | NR | NR |
| NR (Complex oral information transfer) | Nordfalk | 2019 | NR | NR | NR | n/a | n/a | NR | NR | NR | NR |
| NR (Decision-making around limiting life supporting treatment in neonatal intensive care) | Shaw | 2023 | NR | NR | a) associations between scores on decision formats (1 code of subscale 2; recommendation or 'single-option choice' format) and other codes (including total parental response score) per coder; b) range p, ≤.062 to <.01 (coder A), ≤.061 to <.01 (coder B) (pairwise, Bonferroni corrrection) | n/a | n/a | NR | NR | NR | NR |

| Instrument name (acronym) | First author | Year | Content validity^a^ | Structural validity^b^ | Internal consistency^c^ | Cross-cultural  adaptation | Cross-cultural  validity | Criterion validity^d^ | Convergent validity^e^ | Discriminative validity^f^ | Known‐groups validity^g^ |
| --- | --- | --- | --- | --- | --- | --- | --- | --- | --- | --- | --- |
| NR (Global Consultation Rating Scale (GCRS) adapted for pediatric subspecialties) | Coburn | 2020 | NR | NR | NR | n/a | n/a | NR | NR | NR | NR |
| NR (Goal communication in palliative care decision-making consultations) | Gramling | 2015 | NR | NR | NR | n/a | n/a | NR | NR | NR | NR |
| NR (Metaphor use) | Macagno | 2019 | NR | NR | NR | n/a | n/a | NR | NR | NR | NR |
| NR (number and type of problems discussed in primary care consultations) | Procter | 2014 | NR | NR | n/a | n/a | n/a | NR | NR | NR | NR |
| NR (Nurse Patient Touch) | Bottorff | 1994 | NR | NR | NR | n/a | n/a | NR | NR | NR | a) Hypothesis 1: Patterns of touch within each type of attending varies and these patterns are similar across two samples of nurse-patient interactions. Hypothesis 2: Patterns of attending and touch vary in response to different patient states; b) types of attending (Doing more vs. Doing for vs. Doing with vs. Doing tasks), patient states (uncomfortable vs. comfortable-eyes open vs. comfortable-eyes closed); c) social-emotional vs. instrumental touch varied by type of attending in sample 1 (N=56; Chi-square=24.47, *p*=.000) and sample 2 (N=60; Chi-square=25.35, *p*=.000) (hypotheses confirmed); Probabilities of touch events differed by patient state (*p*-value NR) (hypotheses confirmed) |
| Instrument name (acronym) | First author | Year | Content validity^a^ | Structural validity^b^ | Internal consistency^c^ | Cross-cultural  adaptation | Cross-cultural  validity | Criterion validity^d^ | Convergent validity^e^ | Discriminative validity^f^ | Known‐groups validity^g^ |
| NR (Patient active involvement in mental health interaction) | Bonfils | 2017 | NR | NR | a) α; b) .54 (preliminary analyses) | n/a | n/a | NR | a) yes, b) Patient Activation Measure-Mental Health, c) n.s. except for some items with negative associations (range partial correlations, -.14- -.25) (hypotheses not confirmed) | NR | NR |
| NR (physicians' compliance-gaining strategies) | Smith | 2004 | NR | NR | NR | n/a | n/a | NR | NR | NR | NR |
| NR (Planning talk within motivational interviewing) | Copeland | 2017 | NR | NR | NR | n/a | n/a | NR | NR | NR | NR |
| NR (Safety-netting communication behaviors in primary care encounters) | Edwards | 2019 | NR | NR | NR | n/a | n/a | NR | NR | NR | NR |
| NR (the process and content of general practice consultations involving the prescription of antibiotic agents) | Cockburn | 1987 | NR | NR | NR | n/a | n/a | NR | NR | NR | NR |
| NR (transparency in patient-provider communication) | Robins | 2011 | NR | NR | NR | n/a | n/a | NR | NR | NR | NR |
| NR (Verbal interpersonal clinical problem-solving skills) | Pridham | 1980 | NR | NR | NR | n/a | n/a | NR | NR | NR | a) differences in problem-solving categories based on differences in types of clinical encounters examined and clinicians involved; b) analysis of variance of encounter by phase showed differences for 6 of 7 (range *p*'s, .001-.26) in problem-solving categories between encounter types (hypotheses confirmed) |
| NR (Workflow task coding scheme) | Asan | 2015 | NR | NR | NR | n/a | n/a | NR | NR | NR | NR |
| Observing patient involvement in decision making (OPTION^12item^ revised) | Butow | 2010 | NR | NR | NR | n/a | n/a | NR | a) no; b) DAS-O, DSAT; c) r_P_=.730 (DAS-O), .594 (DSAT) (*p*'s<.01) (N=55) | NR | NR |
| Instrument name (acronym) | First author | Year | Content validity^a^ | Structural validity^b^ | Internal consistency^c^ | Cross-cultural  adaptation | Cross-cultural  validity | Criterion validity^d^ | Convergent validity^e^ | Discriminative validity^f^ | Known‐groups validity^g^ |
| Observing patient involvement in decision making (OPTION^12item^ revised) | Weiss | 2008 | NR | NR | NR | n/a | n/a | NR | a) yes; b) IDM; c) r_S_=.14, Bland-Altman test of mean difference: mean difference score=1.11 (CI, 0.66–1.56), Limits of Agreement, -3.94-6.16 (hypotheses confirmed) | NR | NR |
| Observing patient involvement in decision making (OPTION^12item^ revised) (German) modified | Keller | 2013 | NR | NR | a) α; b) .90 | Response scale modified, where 'high standard' (score 4) was changed to 'active involvement of patient is observed' | NR | NR | a) no; b) OPTION^12item^ (revised); c) point-biserial correlations per rater, .23, 43, .62, .83 | NR | a) NR; b) SDM present vs. absent as rated by 2 independent SDM experts who reached consensus; c) Area under the curve (AUC)=0.83 (*p*<.001) |
| Observing patient involvement in decision making (OPTION^12item^ revised) (Italian) | Goss | 2007 | NR | a) assumed; b) 235; c) 2 with Eigenvalue> 1; d) 64.5%; e) .46-.86; authors confirm 1-factor structure because of high proportion of explained variance by first factor (50.3%) and high correlation between the 2 factors (r=.53) | a) α; b) 1-factor solution, .82; 2-factor solution: factor 1, .93, factor 2, .85 | Independent translation by 2 native Italian speakers, compared. Consensus version checked for language fluency by teacher of Italian, then back-translated into English and compared to original version by native English speaker. Expert panel reached agreement on final version. | NR | NR | NR | NR | NR |
| Instrument name (acronym) | First author | Year | Content validity^a^ | Structural validity^b^ | Internal consistency^c^ | Cross-cultural  adaptation | Cross-cultural  validity | Criterion validity^d^ | Convergent validity^e^ | Discriminative validity^f^ | Known‐groups validity^g^ |
| Observing patient involvement in decision making (OPTION^12item^) | Elwyn | 2003 | NR | a) assumed; b) 186; c) forced 1-factor solution; d) 35.2%; e) items 2-4 and 6-12>0.36 (M=.64), item 1: -.10, item 5: .09 | a) α; b) .79 | n/a | n/a | NR | NR | NR | a) OPTION^12item^ score influenced by 1. patient age (negative), 2. sex of clinician (positive in favor of female), 3. qualification of clinician (positive), 4. whether there was equipoise regarding clinical topic (positive); b) patient age, clinician sex, clinician qualification, whether there was equipoise regarding clinical topic (y/n); c) age: r_P_=–.14 (hypotheses confirmed), sex/qualification: associations n.s. (hypotheses not confirmed), equipoise: encounters with equipoise (n=15, 8.1%) higher OPTION score (M=21.6) than encounters without equipoise (M=16.4, weighted t test, *p*<.01) (hypotheses confirmed) |
| Observing patient involvement (OPTION^5item^) | Barr | 2015 | NR | NR | NR | n/a | n/a | NR | a) yes; b) r_P_=.61 (95% Credibility Interval, .54-.68) (hypotheses confirmed) | NR | a) higher OPTION^5item^ scores with patient decision aid compared to usual care; b) consultations in usual care arm vs. patient decision aid arm; b) mean OPTION^5item^ higher in patient decision arms (11.9-35.2 points higher, independent Student's t-test; *p*'s<.001) (hypotheses confirmed) |

| Instrument name (acronym) | First author | Year | Content validity^a^ | Structural validity^b^ | Internal consistency^c^ | Cross-cultural  adaptation | Cross-cultural  validity | Criterion validity^d^ | Convergent validity^e^ | Discriminative validity^f^ | Known‐groups validity^g^ |
| --- | --- | --- | --- | --- | --- | --- | --- | --- | --- | --- | --- |
| Observing patient involvement (OPTION^5item^) | Elwyn | 2013 | NR | NR | NR | Items selected from OPTION^12item^instrument, based on analysis of SDM-models, experience of using OPTION^12item^, and response patterns to OPTION^12item^ in N=215 patient-family physician consultations audio recorded in Canada | NR | NR | NR | NR | NR |
| Observing patient involvement (OPTION^5item^) | Stubenrouch | 2016 | NR | NR | NR | n/a | n/a | NR | NR | NR | NR |
| Observing patient involvement (OPTION^5item^) | Vortel | 2016 | NR | NR | NR | n/a | n/a | NR | a) yes; b) OPTION^12item^ (revised); c) mean OPTION^5item^ =60.6 (SD=12.4) significantly higher than mean OPTION^12item^ =43.8 (SD=9.7; *p*<.001) (hypotheses confirmed) | NR | NR |

| Instrument name (acronym) | First author | Year | Content validity^a^ | Structural validity^b^ | Internal consistency^c^ | Cross-cultural  adaptation | Cross-cultural  validity | Criterion validity^d^ | Convergent validity^e^ | Discriminative validity^f^ | Known‐groups validity^g^ |
| --- | --- | --- | --- | --- | --- | --- | --- | --- | --- | --- | --- |
| Observing patient involvement (OPTION^5item^) (German) | Kölker | 2018 | NR | NR | NR | Independent forward translation by 2 bilingual translators, third translator combined the 2 translations, and consensus reached among the 3 translators. Final German version and translated user manual evaluated during rater training, which led to revisions to manual. | NR | NR | a) yes; b) OPTION^12item^ (revised); c) r_S_=.47 (*p*=.01) (hypotheses confirmed) | NR | NR |

| Instrument name (acronym) | First author | Year | Content validity^a^ | Structural validity^b^ | Internal consistency^c^ | Cross-cultural  adaptation | Cross-cultural  validity | Criterion validity^d^ | Convergent validity^e^ | Discriminative validity^f^ | Known‐groups validity^g^ |
| --- | --- | --- | --- | --- | --- | --- | --- | --- | --- | --- | --- |
| Observing patient involvement (OPTION^5item^) (simplified, Chinese) | Chen | 2020 | NR | NR | a) α; b) .746 (total scale) | Independent forward translation by 2 medical professionals, back translation of merged version by 2 college English teachers familiar with medical research, harmonization based on original, forward and backward translations, and feedback from original OPTION^5item^ developer, translation, evaluation and simplification of user manual, training of 2 raters who then independently rated 5 consultations to check understandability, interpretation, and cultural relevance of the translation, and review and calibration of simplified Chinese OPTION^5item^ through team discussion. | NR | a) OPTION^12item^; b) r_S_=.660 (*p*<.001) | NR | NR | NR |

| Instrument name (acronym) | First author | Year | Content validity^a^ | Structural validity^b^ | Internal consistency^c^ | Cross-cultural  adaptation | Cross-cultural  validity | Criterion validity^d^ | Convergent validity^e^ | Discriminative validity^f^ | Known‐groups validity^g^ |
| --- | --- | --- | --- | --- | --- | --- | --- | --- | --- | --- | --- |
| Observing patient involvement in decision making (OPTION^12item^ revised (German) | Hirsch | 2012 | NR | a) assumed; b) 40; c) 2; d) 64.5%; e) .46-.86 | a) α; b) .90 (full scale) | n/a (authors refer to other publication reporting 4-stage translation process) | n/a | NR | NR | NR | a) NR; b) 2 SDM-experts reporting that SDM had occurred (yes/no); c) point–biserial correlations with total scores of each rater: range r, .30-.51; sum score of all raters: AUC=0.82 (*p*<.001; 95% CI, .70–.90) |
| Observing patient involvement in decision making (OPTION^12item^ revised) | Elwyn | 2005 | NR | a) assumed; b) 186; c) 2 (scree plot); d) 28% for 1-factor solution; e) NR | a) α; b) .68 | n/a | n/a | NR | NR | NR | NR |
| Observing patient involvement in decision making (OPTION^12item^ revised) | Kasper | 2011 | NR | NR | NR | n/a | n/a | NR | a) yes; b) SDM-Q-9, Decisional Conflict Scale (DCS), Control Preferences Scale (CPS, aggregated to 3 scores); c) mean OPTION^12item^ (revised)-mean SDM-Q-9 scores, r_S_=-.01 (*p*=.93), 4 OPTION-SDM-Q-9 item pairs with identical content: range r_S_, -.06-.06; mean OPTION^12item^ (revised)-mean DCS scores, r_S_=.05 (*p*=.66), OPTION^12item^ (revised)-CPS: Kruskal-Wallis test NR, *p*=.87 (hypotheses not confirmed) | NR | NR |
| Observing patient involvement in decision making (OPTION^12item^ revised) | Stubenrouch | 2016 | NR | NR | NR | n/a | n/a | NR | a) no; b) OPTION^12item^ (revised); c) r_P_=.71 (*p*=.01) | NR | NR |

| Instrument name (acronym) | First author | Year | Content validity^a^ | Structural validity^b^ | Internal consistency^c^ | Cross-cultural  adaptation | Cross-cultural  validity | Criterion validity^d^ | Convergent validity^e^ | Discriminative validity^f^ | Known‐groups validity^g^ |
| --- | --- | --- | --- | --- | --- | --- | --- | --- | --- | --- | --- |
| ON-Code | Alby | 2021 | NR | NR | NR | n/a | n/a | NR | a) no; b) VR-CoDES; c) doctor communicative behavior, patient initiatives, interruptions, and accountability and expressions of trust unrelated to VR-CoDES indices (*p*'s>.10); Cues/concerns associated with more misalignments (*p*=.01), greater markers of uncertainty (*p*=.059), less markers of emotions in doctor talk (*p*=.083); Provider provides and Provider reduces related respectively to less misalignments (*p*=.009, *p*=.04), less markers of uncertainty (*p*=.068, *p*=.042), more markers of emotions in doctor talk (*p*=.064, *p*=.078) | NR | a) ONCode sensitive to differences in communication in oncological consultations with native vs. non-native patients *(not further specified)*; b) native vs. non-native (Italian) patients; c) scores on communication dimensions higher for non-natives vs. natives for: doctor communicative behavior, patient initiatives, misalignments, accountability and expressions of trust, markers of uncertainty (range *p,* .001-.063); Note: *p*-value<.010 considered significant. |
| OnePass coding system | McMaster | 2015 | NR | NR | NR | n/a | n/a | a) MITI; b) MITI Evaluative items-OnePass items: range r_P_, .221-.640, M=.634; MITI frequency scores-OnePass items: range r_S_, .038-.500, aggregate score: r_P_=.498, r_S_=.485 | NR | NR | NR |
| Paediatric Dental Triadic Interaction Coding Scheme (PaeD-TrICS) | Yuan | 2019 | NR | NR | n/a | n/a | n/a | NR | NR | NR | NR |
| Parental Engagement Scale (PES) | Kearney | 2011 | NR | NR | NR | n/a | n/a | NR | NR | NR | NR |
| Instrument name (acronym) | First author | Year | Content validity^a^ | Structural validity^b^ | Internal consistency^c^ | Cross-cultural  adaptation | Cross-cultural  validity | Criterion validity^d^ | Convergent validity^e^ | Discriminative validity^f^ | Known‐groups validity^g^ |
| Patient Involvement and Collaboration in Emergency care Teams (PIC-ET) tool | Dubois | 2023 | International expert panel (N=9) established content validity in 3 Delphi rounds through agreement on relevance and relative importance. | NR | NR | n/a | n/a | NR | NR | NR | NR |
| Patient-Centered Behaviour Coding Instrument (PCBI) | Zandbelt | 2005 | NR | a) yes; b) 323; c) 2; d) 31%; e) NR | a) α; b) .64 (Facilitating dimension), .50 (Inhibiting dimension) | n/a | n/a | a) global ratings of EuroCommunication Scale; b) range correlations, .28-.31 (visit level, N=323) (*p*'s<.001), between .48-.53 and -.84–-.83(physician-level, N= 30) (*p*'s<0.01), all as expected. | NR | NR | NR |
| Patient-Centered Communication Coding System (PCCCS) | Ledoux | 2013 | NR | NR | NR | n/a | n/a | NR | NR | NR | NR |
| Pediatric Emergency Discharge Interaction Coding Scheme (PEDICS) | Curran | 2017 | NR | NR | NR | n/a | n/a | NR | NR | NR | NR |
| Pediatric Oncology Prognostic Communication (POPC) codebook | Kaye | 2023 | NR | NR | NR | n/a | n/a | NR | NR | NR | NR |

| Instrument name (acronym) | First author | Year | Content validity^a^ | Structural validity^b^ | Internal consistency^c^ | Cross-cultural  adaptation | Cross-cultural  validity | Criterion validity^d^ | Convergent validity^e^ | Discriminative validity^f^ | Known‐groups validity^g^ |
| --- | --- | --- | --- | --- | --- | --- | --- | --- | --- | --- | --- |
| Perioperative Child–Adult Medical Procedure Interaction Scale (P-CAMPIS) | Caldwell-Andrews | 2005 | NR | NR | NR | n/a | n/a | NR | a) yes; b) modified Yale Preoperative Anxiety Scale; c) children who verbalized resistance, fear, or crying as coded with P-CAMPIS observed to be more anxious during induction of anesthesia (75.5±18.2 vs. 44.2±22.4; *p*<.001) (hypotheses confirmed) | a) yes; b) children who did vs. did not exhibit fear, resistance and/or crying based on coding scheme; c) children who verbalized resistance, fear, or crying scored as more anxious (*p*=.001) (hypotheses confirmed) | NR |
| Person Centered Behavior Inventory (PCBI) | Lann-Wolcott | 2011 | NR | NR | NR | n/a | n/a | NR | a) yes; b) Global Behavior Scale; c) r_P_=.67 (*p*<.01) (hypotheses confirmed) | a) yes; b) Task-Centered Behavioral Inventory (TCBI); c) r_P=-_.35 (*p*<.01) (hypotheses confirmed) | NR |
| Physician Recommendation Coding System (PhyReCS) | Scherr | 2017 | NR | n/a (1 item) | n/a | n/a | n/a | a) patients' perceived recommendations; b) 81% concordance between patients' perceived and coder perceived recommendations | NR | NR | a) physicians' coded recommendation scores higher for treatment patient received vs. nonchosen treatment options; b) treatment received vs. not chosen; c) for each treatment, relative treatment recommendation score higher for individuals who received that treatment vs. other 2 treatments (*p*'s<.01) (hypotheses confirmed) |
| Problem, Analysis or Assessment, and Solution (Pas® System) | Van Mil | 1997 | NR | NR | NR | n/a | n/a | NR | NR | NR | NR |
| Process of Interactional Sensitivity Coding in Healthcare (PISCH) | Sabee | 2015 | NR | NR | NR | n/a | n/a | NR | NR | NR | NR |
| Refined Cue Utilization and Engagement in Dementia (CUeD) | Liu | 2020 | NR | NR | NR | n/a | n/a | NR | NR | NR | NR |
| Instrument name (acronym) | First author | Year | Content validity^a^ | Structural validity^b^ | Internal consistency^c^ | Cross-cultural  adaptation | Cross-cultural  validity | Criterion validity^d^ | Convergent validity^e^ | Discriminative validity^f^ | Known‐groups validity^g^ |
| Resident-centered Assessment of Interactions with Staff and Engagement tool (RAISE) | Snow | 2018 | Expert panel assessed final RAISE using 3-item survey on 11-point scale, asking: (a) how well RAISE captures engagement/activity and interaction, (b) whether RAISE is missing important components, (c) confidence that RAISE ratings over time would reflect actual changes in constructs measured. Expert panel ratings: a) M=8.4, range, 6–10, Md=8; b) M=3, range, 0–8, Md=1; c) M=8.83, range, 7–10, Md=9 | NR | NR | n/a | n/a | NR | NR | NR | NR |
| Rochester Participatory Decision-Making Scale (RPAD) | Shields | 2005 | NR | NR | NR | n/a | n/a | a) patients’ and standardized patients’ perceptions of relationships with physicians; b) r=.32-.36 (standardized patients' perceptions), .06-.07 (real patients' perceptions) (*p*'s<.05) | a) yes; b) MPCC human coding; c) r_P_=.24 (MPCC full scale, *p*<.05), .18 (component 1, *p*<.01), .08 (component 2, n.s.), 0.19 (component 3, *p*<.01) (hypotheses confirmed) | NR | NR |

| Instrument name (acronym) | First author | Year | Content validity^a^ | Structural validity^b^ | Internal consistency^c^ | Cross-cultural  adaptation | Cross-cultural  validity | Criterion validity^d^ | Convergent validity^e^ | Discriminative validity^f^ | Known‐groups validity^g^ |
| --- | --- | --- | --- | --- | --- | --- | --- | --- | --- | --- | --- |
| Rochester Participatory Decision-Making Scale (RPAD) (Chinese) | Guo | 2020 | NR | a) assumed; b) 200 (assumed); c) 3; d) NR; e) .697-.752 (factor 1), .666-.756 (factor 2), .532-.731 (factor 3) | a) α, Spearman-Brown coefficient (half reliability) Guttman coefficient (half reliability); b) .604 (α), .646 (Spearman-Brown), .612 (Guttman) | After consent of original author, Brislin’s method was applied to forward translation (2 independent translators) and backward translation (1 translator, another person compared the back-translation to original version). Experts in clinical, nursing, and humanities ﬁeld analyzed content and range of Chinese version, and judged it to represent original scale well. | NR | NR | NR | NR | NR |

| Instrument name (acronym) | First author | Year | Content validity^a^ | Structural validity^b^ | Internal consistency^c^ | Cross-cultural  adaptation | Cross-cultural  validity | Criterion validity^d^ | Convergent validity^e^ | Discriminative validity^f^ | Known‐groups validity^g^ |
| --- | --- | --- | --- | --- | --- | --- | --- | --- | --- | --- | --- |
| Roter Interaction Analysis System (RIAS) * | Roter | 2002 | Unclear | Unclear | Unclear | n/a | n/a | Unclear | Unclear | Unclear | a) yes; b) oncologists vs. general practitioners, cancer vs. general practice patients; c) oncologists vs. general practitioners: gave more information and showed less counselling (instrument clusters; *p*<.001), more verbally attentive, expressed more concern (affective clusters) and higher rated on ‘interest:engagement’ and ‘friendliness:warmth’ (global scales) (n.s. in some GP samples); cancer vs. general practice patients: differences in asking questions (instrumental cluster), expressing concerns (affective cluster), rating on ‘anxiety:nervousness’ (global scales) n.s. (hypotheses not confirmed) |
| SDM-18 | Salyers | 2012 | NR | NR | NR | n/a | n/a | NR | NR | NR | NR |

| Instrument name (acronym) | First author | Year | Content validity^a^ | Structural validity^b^ | Internal consistency^c^ | Cross-cultural  adaptation | Cross-cultural  validity | Criterion validity^d^ | Convergent validity^e^ | Discriminative validity^f^ | Known‐groups validity^g^ |
| --- | --- | --- | --- | --- | --- | --- | --- | --- | --- | --- | --- |
| Segue framework | Makoul | 2001 | Checks with (resident/) physicians, students, standardized patients, instructors and researchers indicated that tasks were understood as intended. Coders applied framework to 500 videotaped primary care interactions to check if all behaviors are performed and relevant: 20/23 tasks were observed in majority of encounters. | NR | n/a | n/a | n/a | a) standardized patients' confidence in competence (1 item: 'would you choose this student to be your doctor?') on 5-point rating scale; b) r=.65, N=134, *p*<.001 | a) no; b) American Board of Internal Medicine 'patient satisfaction questionnaire'; c) r=.49 (*p*<.001) (N=169) | NR | NR |
| Siminoff Communication Content & Affect Program (SCCAP) | Siminoff | 2011 | NR | NR | NR | n/a | n/a | NR | NR | NR | a) NR; b) consent/refusal to donate; c) higher provider affect associated with higher likelihood of consent to donate (*p*<.001); higher disconfirmation associated with more refusal to donate (*p*<.01) |
| St Andrews Behavioural Interaction Coding Scheme (SABICS) | Zhou | 2012 | NR | NR | NR | n/a | n/a | NR | NR | NR | a) frequency of 11 nurse behaviors (controlling for duration of interactions) associated with successful application of varnish; b) (un)successful application of varnish; c) both Instruction and Praise positively associated (Odds Ratio resp. 1.32 and 2.04 (*p*<.05) (hypotheses confirmed) |
| Instrument name (acronym) | First author | Year | Content validity^a^ | Structural validity^b^ | Internal consistency^c^ | Cross-cultural  adaptation | Cross-cultural  validity | Criterion validity^d^ | Convergent validity^e^ | Discriminative validity^f^ | Known‐groups validity^g^ |
| State Space Grids | Gainforth | 2019 | NR | NR | NR | n/a | n/a | NR | NR | NR | NR |
| Taxonomy of Verbal Response Modes (VRM) | Stiles | 1979 | NR | a) yes; b) 52; c) 7; d) medical history (2 factors), 73.8%; physical examination (2 factors), 76.6%; conclusion (3 factors), 70.2%; e) NR | NR | n/a | n/a | NR | NR | NR | NR |
| Taxonomy of Verbal Response Modes (VRM) | Stiles | 1982 | NR | a) yes; b) 115; c) medical history segments (2 factors), physical examination (4 factors), conclusion (5 factors); d) 70.1% (medical history), 74.0% (physical examination), 69.6% (conclusion); e) NR | NR | n/a | n/a | NR | NR | NR | NR |

| Instrument name (acronym) | First author | Year | Content validity^a^ | Structural validity^b^ | Internal consistency^c^ | Cross-cultural  adaptation | Cross-cultural  validity | Criterion validity^d^ | Convergent validity^e^ | Discriminative validity^f^ | Known‐groups validity^g^ |
| --- | --- | --- | --- | --- | --- | --- | --- | --- | --- | --- | --- |
| Verona coding definitions of emotional sequences (VR-CoDES) | Eide | 2011 | NR | NR | NR | n/a | n/a | a- b) by conducting meta-interviews with patients, reflecting on their consultations. Interviews used a researcher-directed approach (RDA, N=6) or patient-directed approach (PDA, N=6). Codes assigned by researcher were (dis)confirmed, and any missed instances were identified. Sensitivity was .95 (RDA) and .99 (PDA); specificity .99 (RDA) and .70 (PDA). Through qualitative analysis 83% of researcher-identified cues and concerns were validated, 17% not confirmed or uncertain. | NR | NR | NR |
| Verona Coding Definitions of Emotional Sequences (VR-CoDES) | Vatne | 2010 | NR | NR | NR | n/a | n/a | NR | NR | NR | NR |
| Verona Coding Definitions of Emotional Sequences (VR-CoDES) | Wright | 2012 | NR | NR | n/a | n/a | n/a | NR | NR | NR | NR |
| Verona coding definitions of emotional sequences (VR-CoDES) | Zimmermann | 2011 | NR | NR | NR | n/a | n/a | NR | NR | NR | NR |
| Instrument name (acronym) | First author | Year | Content validity^a^ | Structural validity^b^ | Internal consistency^c^ | Cross-cultural  adaptation | Cross-cultural  validity | Criterion validity^d^ | Convergent validity^e^ | Discriminative validity^f^ | Known‐groups validity^g^ |
| Verona Coding Definitions of Emotional Sequences (VR-CoDES) - Chinese | Yin | 2019 | NR | NR | n/a | Translation and back-translation by 4 authors and one bilingual expert, validation of translation through involvement of original measure developers and expert focus group. | Translation was good and easily understood, but some coding examples do not fit into Chinese expression, more examples of patients’ expressions need to be gathered | a) researcher-directed approach (RDA) was used by inviting 2 simulated patients to confirm identified cues/concerns; b) simulated patients confirmed 541/549 coded emotions: 517/525 cues and 24/24 concerns. They identified 25 extra (uncoded) cues. Specificity: .99 (566/574), sensitivity: .96 (541/566). | NR | NR | NR |
| Verona Coding Definitions of Emotional Sequences for client cues and concerns (VR-CoDES- CC) | Vijfhuizen | 2017 | NR | NR | NR | Pilot study on 6 consultations performed to determine necessary modifications to original VR-CoDES. Based on discussion, new notations introduced to identify perspective and direction of cues and concerns, coding reminders created (codes unchanged). | NR | NR | NR | NR | NR |
| Instrument name (acronym) | First author | Year | Content validity^a^ | Structural validity^b^ | Internal consistency^c^ | Cross-cultural  adaptation | Cross-cultural  validity | Criterion validity^d^ | Convergent validity^e^ | Discriminative validity^f^ | Known‐groups validity^g^ |
| Verona Coding Definitions of Emotional Sequences for health provider responses (VR-CoDES-P) | Del Piccolo | 2011 | NR | NR | NR | n/a | n/a | NR | NR | NR | NR |
| Verona Coding Definitions of Emotional Sequences for health provider responses (VR-CoDES-P) | Wright | 2012 | NR | NR | n/a | n/a | n/a | NR | NR | NR | NR |
| Verona Coding Definitions of Emotional Sequences for health provider responses (VR-CoDES-P) - Chinese | Yin | 2019 | NR | NR | n/a | Translation and back-translation by 4 authors and one bilingual expert, validation of translation through involvement of original measure developers and expert focus group. | Translation was good and easily understood, but some coding examples do not fit into Chinese expression, more examples of patients’ expressions need to be gathered | NR | NR | NR | NR |

| Instrument name (acronym) | First author | Year | Content validity^a^ | Structural validity^b^ | Internal consistency^c^ | Cross-cultural  adaptation | Cross-cultural  validity | Criterion validity^d^ | Convergent validity^e^ | Discriminative validity^f^ | Known‐groups validity^g^ |
| --- | --- | --- | --- | --- | --- | --- | --- | --- | --- | --- | --- |
| Verona coding definitions of emotional sequences to code health providers’ responses (VR-CoDES-P) | Vijfhuizen | 2017 | NR | NR | NR | Pilot study on 6 consultations performed to determine necessary modifications to original VR-CoDES. Based on discussion of coding challenge, new notations introduced to identify perspective and direction of cues and concerns, and reminders created to assist with coding (codes unchanged). | n/a | NR | NR | NR | NR |
| Verona Medical Interview Classification System (VR-MICS) | Del Piccolo | 2004 | NR | a) yes; b) 238; c) 7; d) 62.5%; e) .35-.86 | NR | n/a | n/a | NR | NR | NR | NR |

| Instrument name (acronym) | First author | Year | Content validity^a^ | Structural validity^b^ | Internal consistency^c^ | Cross-cultural  adaptation | Cross-cultural  validity | Criterion validity^d^ | Convergent validity^e^ | Discriminative validity^f^ | Known‐groups validity^g^ |
| --- | --- | --- | --- | --- | --- | --- | --- | --- | --- | --- | --- |
| Verona medical interview classification system (VR-MICS) | Del Piccolo | 2005 | NR | NR | NR | n/a | Both samples were obtained in European countries with national health service; providers matched by gender, age, experience; patients matched by gender, GHQ-12 score, age, illness; Italy: 6 GPs, 30 consultations; UK: 6 GPs, 30 consultations. Range Dice similarity index, .43 -.94 (VR-MICS/D), .50-.95 (VR-MICS/P) | NR | NR | NR | NR |
| Vr-COPE | McDermott | 2021 | NR | NR | NR | n/a | n/a | NR | NR | NR | NR |

**Notes.**

^a^ Item pool relevance / comprehensiveness

^b^ a) reflective model; b) sample size; c) number of factors; d) percentage variance explained; e) range of factor loadings

^c^ a) type of scale; b) results

^d^ a) gold standard / comparator(s); b) results

^e^ a) clear hypotheses; b) comparator(s); c) results

^f^ a) clear hypotheses; b) comparator(s); c) results

^g^ a) hypotheses; b) subgroups; c) results

* The original publication could not be retrieved. Information was based on Roter et al. (2002) and Ong et al. (1998)

Abbreviations: #, number; α, Cronbach's alpha; CFI, comparative fit index; CI, Confidence interval; ICC, Intraclass correlation coefficient; NR, not reported; r_P_, Pearson's correlation coefficient; RMSEA, root mean square error of approximation; r_S_, Spearman's rho coefficient; SRMR, standardized root mean square residual
